# Metal–Ligand Cooperative Nitrile Activation Triggered by a Catalytic Amount of Weak Base

**DOI:** 10.1021/acs.organomet.6c00104

**Published:** 2026-09-01

**Authors:** Stanislav Melnikov, Arie J. H. Multem, Pieter N. Oostenbrug, Martin Lutz, Daniël L. J. Broere

**Affiliations:** † Organic Chemistry and Catalysis, Institute for Sustainable and Circular Chemistry, Faculty of Science, 8125 Utrecht University, Universiteitsweg 99, 3584 CG Utrecht, The Netherlands; ‡ Structural Biochemistry Bijvoet Centre for Biomolecular Research, Faculty of Science, Utrecht University, Universiteitsweg 99, 3584 CG Utrecht, The Netherlands

## Abstract

Nitriles are common functional groups in chemical building blocks, yet their direct conversion into diverse functional groups typically requires forcing conditions due to the thermodynamic stability of the C≡N bond. Activation of nitriles through metal–ligand cooperativity (MLC) has emerged as a powerful strategy and typically involves the use of strong bases to generate a reactive complex featuring a dearomatized pincer ligand. Herein, we report a mononuclear ruthenium platform supported by a 1,8-naphthyridine-based PNNP ligand that enables nitrile activation via MLC using only catalytic amounts of the weak base triethylamine. Combined experimental and computational mechanistic studies reveal a mechanism proceeding via an initial endergonic ligand deprotonation that is followed by nitrile coordination, intramolecular nucleophilic attack and reprotonation of the resulting ketimido nitrogen by the conjugate acid of the base. In addition, we show that bimetallic analogues undergo two concurrent metal–ligand cooperative activation events of two acetonitrile molecules to form diastereomeric bis-ketimine complexes.

## Graphical abstract

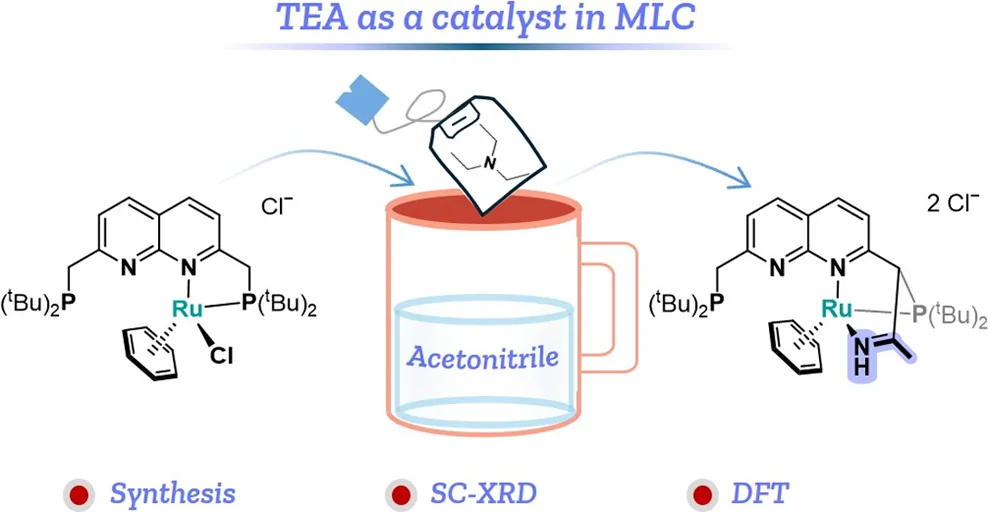

## Introduction

Nitrile functionalities are ubiquitous in biologically active molecules and play a crucial role in the activity of numerous pharmaceuticals.^1–3^ Beyond medicinal chemistry, nitriles are versatile and widely used functional groups in modern organic synthesis, serving as valuable precursors to amides, amines, carboxylic acids, ketones, and heterocycles.^4,5^ A wide range of strategies for nitrile functionalization has been established, many of which rely on homogeneous catalysis mediated by metal complexes.^6,7^ Within this context, pincer complexes have emerged as particularly powerful platforms due to their robustness, tunability, and ability to promote challenging bond-forming reactions. A defining feature that underpins the exceptional reactivity of many pincer systems is metal–ligand cooperativity (MLC), wherein both the metal center and the ligand actively participate in substrate activation and bond making or breaking events.^8^

Pioneering contributions to the subject of MLC-mediated nitrile activation from the groups of Milstein,^9,10^ and Otten^11,12^ utilize dearomatized pyridine-based pincer complexes featuring a highly nucleophilic methine linker (Figure 1A). This nucleophilic carbon is sufficiently reactive to attack the electrophilic carbon of a nitrile (C≡N) group, resulting in the formation of a metal-bound ketimido complex. Remarkably, this mode of nitrile activation was shown to be reversible, enabling downstream functionalization of the activated nitrile, involving deuteration,^13^ hydration toward amide products,^13^ and coupling reactions with aldehydes^14^ or α,β-unsaturated nitriles.^9,15,16^ These discoveries highlighted the unique potential of MLC-enabled pincer complexes to mediate C–C bond formation involving nitrile substrates.

**Figure 1:**
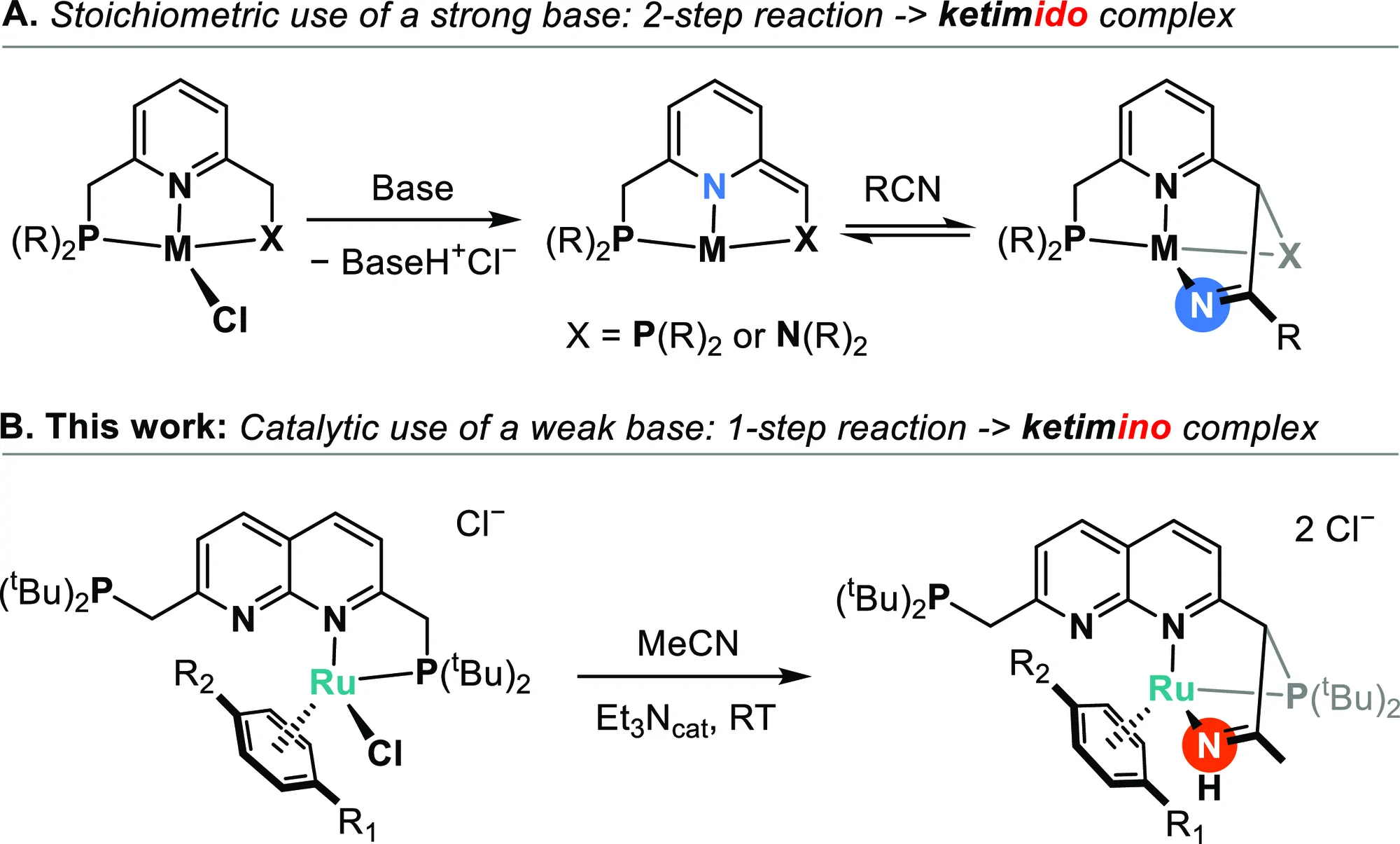
Previously reported MLC-driven activation of nitriles using pincer complexes (A) and this work using the 1,8-naphthyridine-based PNNP ligand (B).

An inherent mechanistic feature of MLC-based nitrile activation involving pyridine-based pincer ligands is the employment of stoichiometric quantities of strong bases, such as KO^*t*^Bu or KHMDS, to access the reactive dearomatized state. In this work, we examine ruthenium complexes supported by a 1,8-naphthyridine-based ligand framework and show that their reactivity toward nitriles differs markedly from that of related pyridine-based systems. The increased acidity of the methylene linkers in the naphthyridine scaffold enables ligand dearomatization to occur under significantly milder basic conditions, using weak organic bases such as triethylamine (Et_3_N). It reveals an alternative regime of metal–ligand cooperative nitrile activation that operates under catalytic base conditions. These observations highlight how changes in ligand architecture can fundamentally alter the balance between acidity, dearomatization, and substrate activation in systems that employ MLC.

## Results and Discussion

In previous work, we reported the synthesis of a ruthenium complex featuring a coordinated arene and a chelating pyridine-based PN ligand.^17^ Building on this, we explored the use of a closely related naphthyridine-based PNNP ligand which had been previously synthesized in our group^18,19^ and recently was used to construct heterobimetallic complexes.^20^ Analogous to the synthesis of PN–RuCl­(benzene)­PF_6_,^17^ the targeted ruthenium complexes bearing the PNNP ligand were prepared via the reaction of the PNNP ligand with 0.5 equiv of Ru_2_Cl_4_(benzene)_2_ or its more soluble analogue, Ru_2_Cl_4_(*p*-cymene)_2_, in DCM at room temperature (Scheme 1). The resulting complexes, **1** and **2**, were isolated in good yields of 77% and 89%, respectively. The ^1^H NMR of complex **1** (CD_2_Cl_2_) features four doublets in the range δ = 8.45–7.79 ppm, each integrating to 1H, which is characteristic of nonsymmetric mononuclear complexes supported by the PNNP ligand.^20^ In addition, a singlet integrating to 6H is observed at δ = 6.54 ppm, along with two doublets of doublets at δ = 4.29 and 4.03 ppm (1H each). These resonances are indicative of an η^6^-coordinated benzene ligand and diastereotopic methylene protons, respectively, closely resembling the spectroscopic features previously reported for PN–RuCl­(benzene)­PF_6_.^17^ Another doublet at δ = 3.52 ppm integrating to 2H suggests that the second methylene linker resides in a symmetric environment and that the second coordination pocket of the PNNP ligand is not involved in metal binding. Consistent with this assignment, the ^31^P NMR spectrum displays two singlets at δ = 86.4 and 38.2 ppm, with the latter close to the value of the free ligand, supporting the mononuclear nature of the complex.

**Scheme 1:**
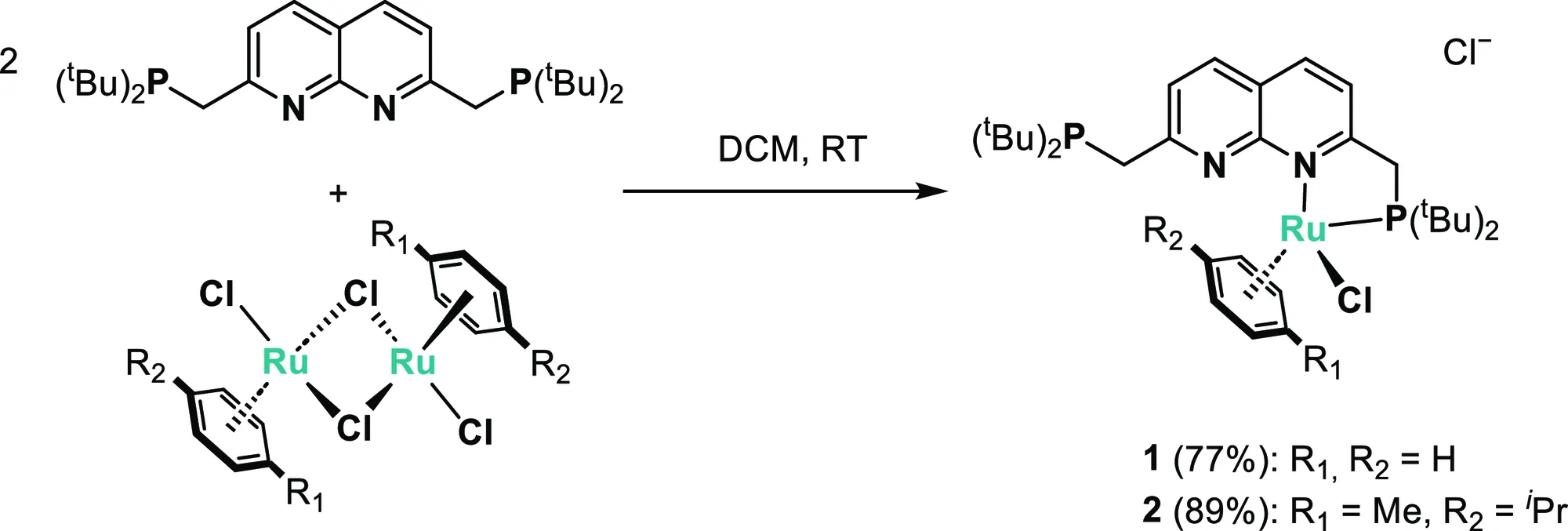
Synthesis of Complexes **1** and **2**

In contrast, the ^1^H NMR spectrum of complex **2** (CD_2_Cl_2_) is significantly broadened. Similar to complex **1**, four signals are observed between δ = 8.46 and 7.81 ppm; however, only two appear as sharp doublets. The resonances associated with the η^6^-coordinated *p*-cymene fragment are heavily broadened, suggesting the existence of a fluxional process. Notably, a doublet of doublets of doublets, corresponding to the methylene linker of the Ru-side, at δ = 3.45 ppm (2H), along with doublets at δ = 1.53, 1.20, and 1.16 ppm integrating as 9H each, remain sharp and well-defined. Similarly, the ^31^P NMR spectrum shows two closely spaced, broadened sets of singlets at δ = 83.7–85.2 and 36.7–37.4 ppm. The close similarity of these chemical shifts to those observed for complex **1** suggests that the reaction of the PNNP ligand with Ru_2_Cl_4_(*p*-cymene)_2_ affords an analogous mononuclear complex bearing a *p*-cymene ligand. The pronounced line broadening in the NMR spectra of complex **2** can be rationalized by a fluxional process involving rotation of the *p*-cymene ligand, giving rise to two rotamers in which either the methyl or the isopropyl substituent is oriented toward the chloride ligand. Consistent with this interpretation, the ^1^H NMR spectrum of complex **2** recorded at 45 °C shows significantly sharper resonances, while the ^31^P NMR spectrum reveals coalescence of the four signals into two (Figures S15–S18). The appearance of two sets of NMR signals for complex **2** at room temperature is presumably attributed to the restricted rotation of the coordinated *p*-cymene ligand, which gives rise to two distinct rotameric species in solution on the NMR time scale (supported by DFT calculations, see Figure S69). The observed room-temperature line broadening is most consistent with dynamic rotation of the coordinated *p*-cymene ligand. However, in the absence of low-temperature NMR data, contributions from other dynamic processes, such as transient PNNP-ligand hemilability or chloride exchange, cannot be excluded completely.

Single crystals suitable for X-ray diffraction analysis were obtained by slow evaporation of a saturated dichloromethane solution of **1-PF**_**6**_, prepared via a salt metathesis with KPF_6_. The molecular structure in the crystal is fully consistent with the structure proposed on the basis of solution NMR spectroscopy (Figure 2). The asymmetric unit consists of two independent molecules of the complex. The ruthenium center occupies one coordination pocket of the PNNP ligand, binding to one nitrogen and one phosphorus donor atom. The benzene ligand is coordinated in an η^6^-fashion, while a chloride ligand completes the coordination sphere of the metal.

**Figure 2:**
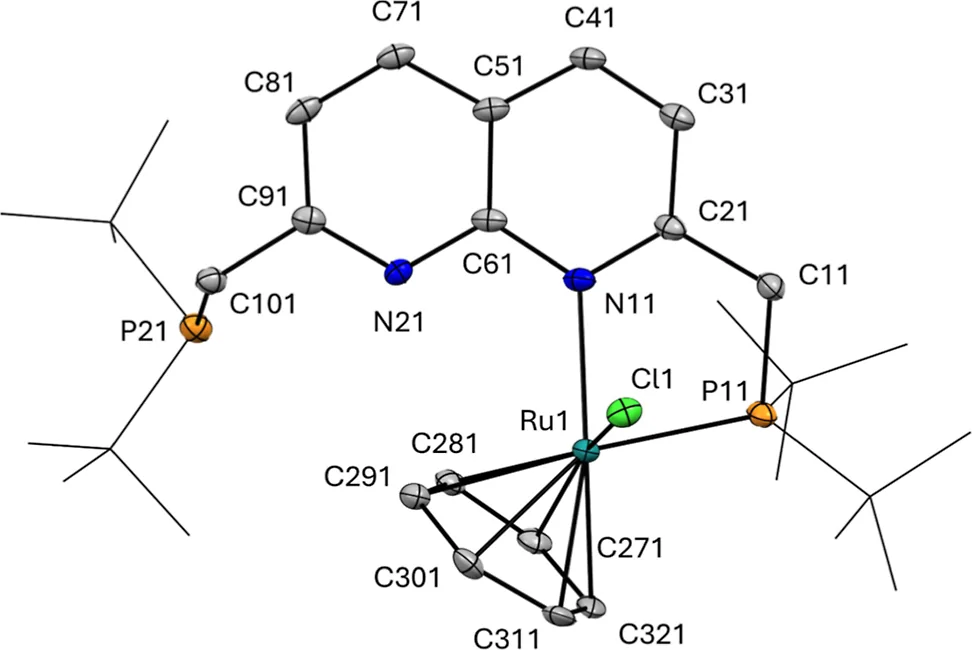
Molecular structure of complex **1-PF**_**6**_ obtained by single crystal X-ray structure determination (only the cation is shown). Only one independent molecule of the complex of the asymmetric unit is shown, cocrystallized DCM molecules are not shown. The hydrogen atoms and PF_6_ anions are omitted for clarity. The displacement ellipsoids are shown with 30% probability. Key bond lengths and angles: Ru–Arene^Centr^: 1.719(3)/1.722(3) Å, Ru–N^Py^: 2.183(5)/2.199(5) Å, Ru–P: 2.3559(18)/2.3456(18) Å, P–Ru–N^Py^: 78.28(15)/80.09(16)°, P–Ru–Arene^Centr^: 132.02(11)/132.12(12)°, N^Py^–Ru–Arene^Centr^: 135.07(19)/135.87(19)°.

The overall structure of complex **1-PF**_**6**_ closely resembles that of the previously reported PN–RuCl­(benzene)­PF_6_ complex.^17^ The C–C distances of the PNNP ligand (Table S1) are consistent with the neutral form with an aromatic backbone.^18,20^ The Ru–C^Benzene^ distances can be clustered into two groups based on their lengths (shorter distances with C­(32X), C­(31X), C­(28X) compared with slightly more elongated distances with C­(29X), C­(30X), C­(27X)), similarly to previously reported PN–RuCl­(benzene)­PF_6_ complex by our group.^17^ The longest distances are observed for C­(29X) and C­(30X) atoms, which are situated in the anticonfiguration against the coordinated phosphine. This can be explained by a *trans*-effect of the phosphine ligand.^21,22^

While working with impure batches of complex **2** in MeCN, we observed the rapid formation of new species (**4**, Scheme 2) with distinct NMR signatures. Based on its spectroscopic features (discussed below), this species was hypothesized to be the product of nitrile-activation via MLC. In attempts to investigate this reaction, we found that pure batches of the starting complex are fully stable in MeCN. Hence, we reasoned that the observed reactivity in the impure samples could be triggered by trace amounts of a weak base. To test this, we added stoichiometric amounts of Et_3_N to DCM solutions of complexes **1** or **2**. ^1^H NMR analysis of these mixtures showed no conversion of either complex, consistent with the relatively weak basicity of Et_3_N. In contrast, the addition of a catalytic amount of Et_3_N (10 mol %) to MeCN solutions of complexes **1** or **2** (Scheme 2) led to a gradual color change from dark orange to yellow, accompanied by a partial formation of yellow precipitate.

**Scheme 2:**
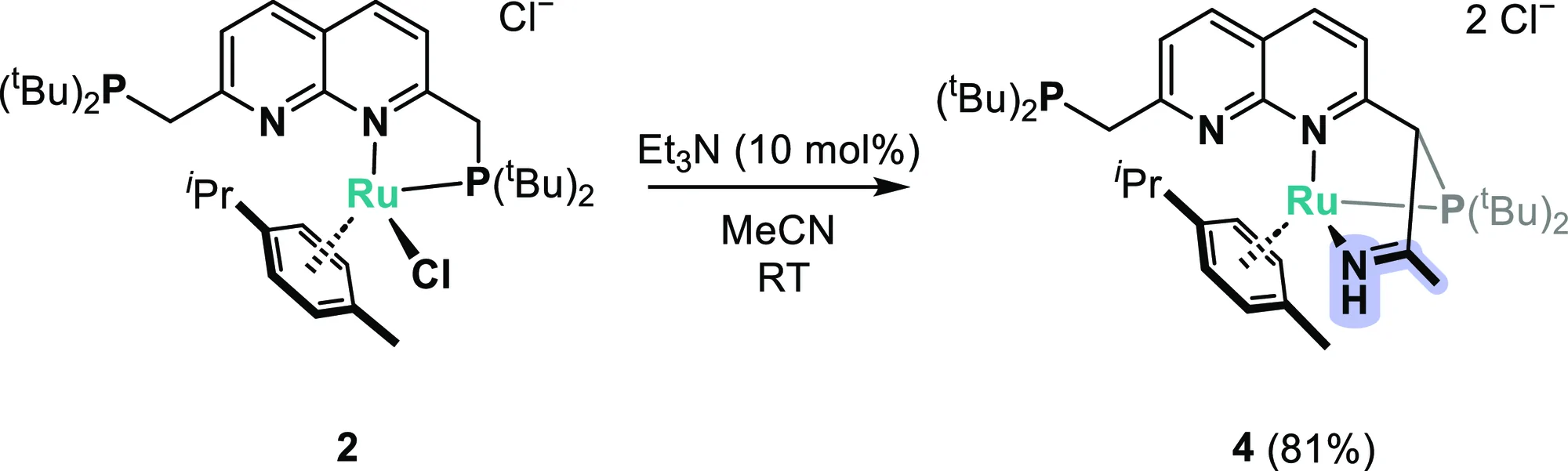
Reactions of Complex **2** with MeCN in the Presence of a Catalytic Amount of Et_3_N

Analysis of the reaction product by ^1^H NMR spectroscopy (in MeCN-*d*_3_) revealed a distinct broad singlet at δ = 14.40 ppm, indicative of MLC activation of acetonitrile and characteristic of an aldimine or ketimine C=NH functionality. This signal is significantly more deshielded compared to previous cases of MLC-type activation of acetonitrile where the NH signal was observed at δ ∼ 10.00 ppm.^9,12^ The spectrum retained features associated with the coordinated *p*-cymene ligand and additionally displayed a doublet at δ = 3.60 ppm, which was assigned to the methylene linker of the free pocket of the PNNP ligand based on ^1^H–^31^P HMBC correlations. A second doublet at δ = 6.26 ppm (integrating as 1H) exhibited clear ^1^H–^31^P coupling to the phosphorus atom coordinated to the ruthenium center. Notably, a singlet at δ = 2.70 ppm (integrating as 3H) was observed, consistent with the presence of α-protons of a ketimine moiety.

The ^31^P­{^1^H} NMR spectrum showed a singlet at δ = 39.0 ppm, comparable to that of the starting complex and attributable to the phosphorus atom in the noncoordinated ligand pocket.^23^ In contrast, the second phosphorus resonance was dramatically downfield shifted to δ = 169.4 ppm, relative to 85.17 ppm in the starting complex. Such pronounced deshielding of the phosphorus atoms is consistent with literature reports of nitrile-activated PNP-type complexes.^10^ Interestingly, we found that an analogous complex [PN–Ru­(benzene)­Cl]Cl bearing a pyridine-based PN ligand does not mediate MLC-driven activation of MeCN under these conditions.

^1^H NMR analysis of the products of an analogous reaction of complex **1** (Figures S24–S30) showed similar spectroscopic features that indicate formation of complex **3** as a result of MeCN activation by MLC. In particular the characteristic broad singlet at δ = 15.34 ppm and a singlet at δ = 6.85 ppm integrating to 6H, assigned to the ketimine C=NH proton and an η^6^-coordinated benzene, respectively, support the formation of a complex analogous to **4**.

Crystals of complex **4** were grown from a saturated MeCN solution. The solid-state structure (Figure 3) is fully consistent with the NMR spectroscopic data. The key bond lengths between the ligand and the Ru center are similar between complexes **1-PF**_**6**_ and **4** (Table 1). The activated acetonitrile fragment has a new C–C bond between the methylene linker carbon of the PNNP ligand and the former sp-carbon of the acetonitrile molecule. The nitrogen atom of the MLC-activated acetonitrile is coordinated to the ruthenium center, analogous to previously reported pincer complexes featuring MLC-activated nitrile substrates.^9^

**Figure 3:**
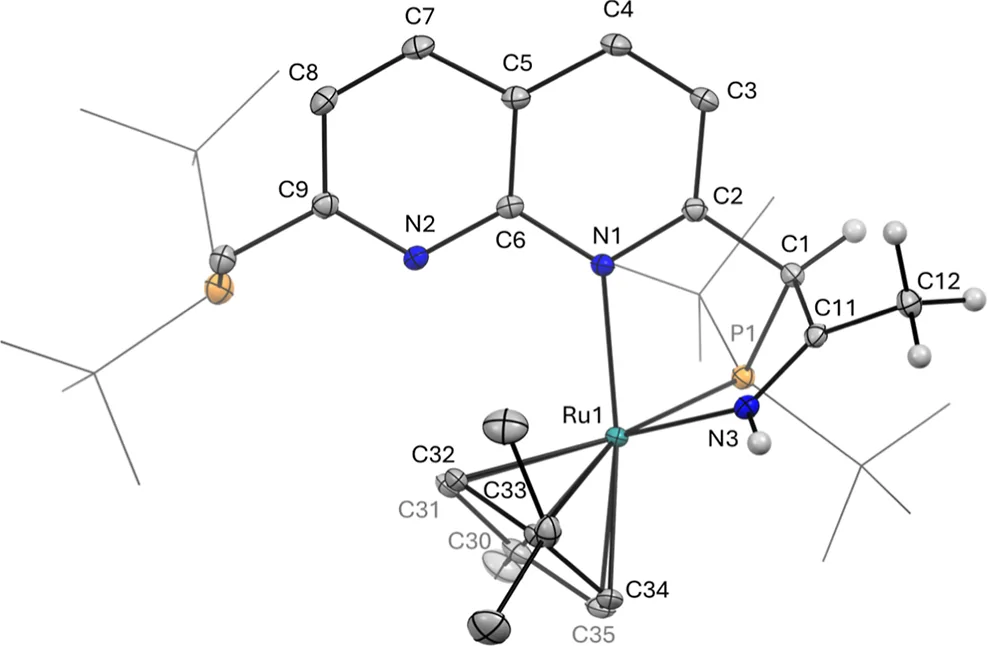
Molecular structure of complex **4** obtained by single crystal X-ray structure determination (only the cation is shown). Only selected hydrogen atoms are shown. The cocrystallized MeCN solvent molecules and two noncoordinated chloride anions are omitted for clarity. The displacement ellipsoids are shown with 30% probability. Key bond lengths and angles: Ru–Arene^Centr^: 1.7340(8) Å, Ru–N^Py^: 2.1543(14) Å, Ru–P: 2.3496(4) Å, P–Ru–N^Py^: 77.28(4)°, P–Ru–Arene^Centr^: 137.34(4)°, N^Py^–Ru–Arene^Centr^: 134.62(5)°, Ru–N^MeCN^: 2.0592(14) Å.

**Table 1:** Comparison of the Key Geometric Parameters of Complexes **1-PF**_**6**_ and **4** Derived from the X-ray Analysis

atoms	complex **1-PF**_**6**_	complex **4**
Ru–Arene^Centr^	1.719(3)/1.722(3) Å	1.7340(8) Å
Ru–N^Py^	2.183(5)/2.199(5) Å	2.1543(14) Å
Ru–P	2.3559(18)/2.3456(18) Å	2.3496(4) Å
P–Ru–N^Py^	78.28(15)/80.09(16)°	77.28(4)°
P–Ru–Arene^Centr^	132.02(11)/132.12(12)°	137.34(4)°
N^Py^–Ru–Arene^Centr^	135.07(19)/135.87(19)°	134.62(5)°
Ru–N^MeCN^	–	2.0592(14) Å

The ruthenium center occupies one coordination pocket of the PNNP ligand and additionally binds an η^6^-coordinated *p*-cymene ligand. Two chloride anions are present in the structure and engage in hydrogen-bonding interactions with the N3–H and C1–H protons, displaying N3···Cl and C1···Cl distances of 3.1666(16) and 3.4807(18) Å, respectively. The N3···Cl interaction is comparable to values previously reported for related systems.^24^ Notably, the short C1···Cl contact suggests the possible presence of a rare C–H···Cl hydrogen-bonding interaction.^25^ The existence of such an interaction may partially account for the unusually downfield-shifted ^1^H NMR resonance observed for this proton. The N3–C11 distance (Table S2) of the activated acetonitrile moiety is found to be 1.274(2) Å, which corresponds to significant loss of the triple bond character and is close to the values found for the similar ketimine complexes.^26^

Surprisingly, we did not observe the expected NH bands at ∼3300 cm^–1^ in the IR spectra of complex **4** (Figure S39). Based on the highly deshielded NH protons according to the ^1^H NMR as well as the results of X-ray crystal structure, we hypothesized that the strong hydrogen bonding with the chloride anion also persists in solution. Strong hydrogen bonding weakens the N–H bond, causing its stretching vibration to shift to lower frequencies, often around 3000 cm^–1^ or below.^27^

Overall, our experimental data suggest that, although direct stoichiometric reaction of the PNNP complexes with Et_3_N does not yield observable deprotonated species, an equilibrium between protonated and deprotonated states may still be accessible at room temperature. The low population of the deprotonated species likely renders it undetectable by standard NMR techniques, yet it is sufficient to facilitate subsequent nitrile activation. This behavior contrasts with previous reports, where strong bases were required to access the dearomatized form necessary for MLC-mediated nitrile activation. The only similar examples of acetonitrile activation using Et_3_N possibly acting as a catalyst are known for transition metal complexes, mainly iron complexes with Schiff bases,^28,29^ although the mechanism in those reactions has not been studied in detail. Formation of ketimine species has also been reported for CNC dicarbene pincer complexes, but only in the presence of strong bases such as phosphazenes, and no mechanistic studies have been conducted.^30^ Motivated by the surprising reactivity of our system with a weak base like Et_3_N, we sought to investigate the underlying reaction mechanism.

We hypothesized that the formation of **3** and **4** proceeds via an endergonic deprotonation of the PNNP ligand by Et_3_N, generating a nucleophilic methine group that attacks the electrophilic carbon of MeCN. Due to the relatively high acidity of Et_3_NH^+^, it can act as an acid to protonate the resulting ketimido moiety to form the ketimino complexes **3** or **4**. To probe this mechanism, two isotopic labeling experiments were performed (Figure 4): (A) the reaction of complex **2** in deuterated acetonitrile (MeCN-*d*_3_), and (B) the reaction of the deuterated analogue **2-D** in nondeuterated MeCN. Surprisingly, in the first experiment, the resulting product **4-D** ^**MeCN**^ showed deuteration not only of the methyl group of the activated acetonitrile molecule but also at the signal corresponding to the C=N*H* group according to both ^1^H and ^2^H NMR (∼90% deuteration). In contrast, the second experiment (B) produced complex **4-D** ^**PNNP**^ in which the C=NH resonance was not deuterated and only the methylene and the methine linkers remained deuterated. Consistent with the observed deuteration in the ^1^H and ^2^H NMR spectra, the IR spectra of both **4-D** ^**MeCN**^ and **4-D** ^**PNNP**^ exhibit multiple absorptions in the 2250–2050 cm^–1^ region, characteristic of C–D and N–D stretching vibrations.^27^ However, due to the many overlapping resonances in this region, we were unable to assign these bands (see Figure S68). These observations suggested that the proton of the ketimine group may not originate from the ligand itself, but rather from the α-protons of the acetonitrile. An alternative explanation would be that, after product formation, the NH proton undergoes rapid exchange with the α-protons of excess acetonitrile. While no exchange of the NH proton is observed upon dissolving **4** in MeCN-*d*_3_ by ^1^H NMR spectroscopy, the addition of Et_3_N results in gradual disappearance of the characteristic NH resonance.

**Figure 4:**
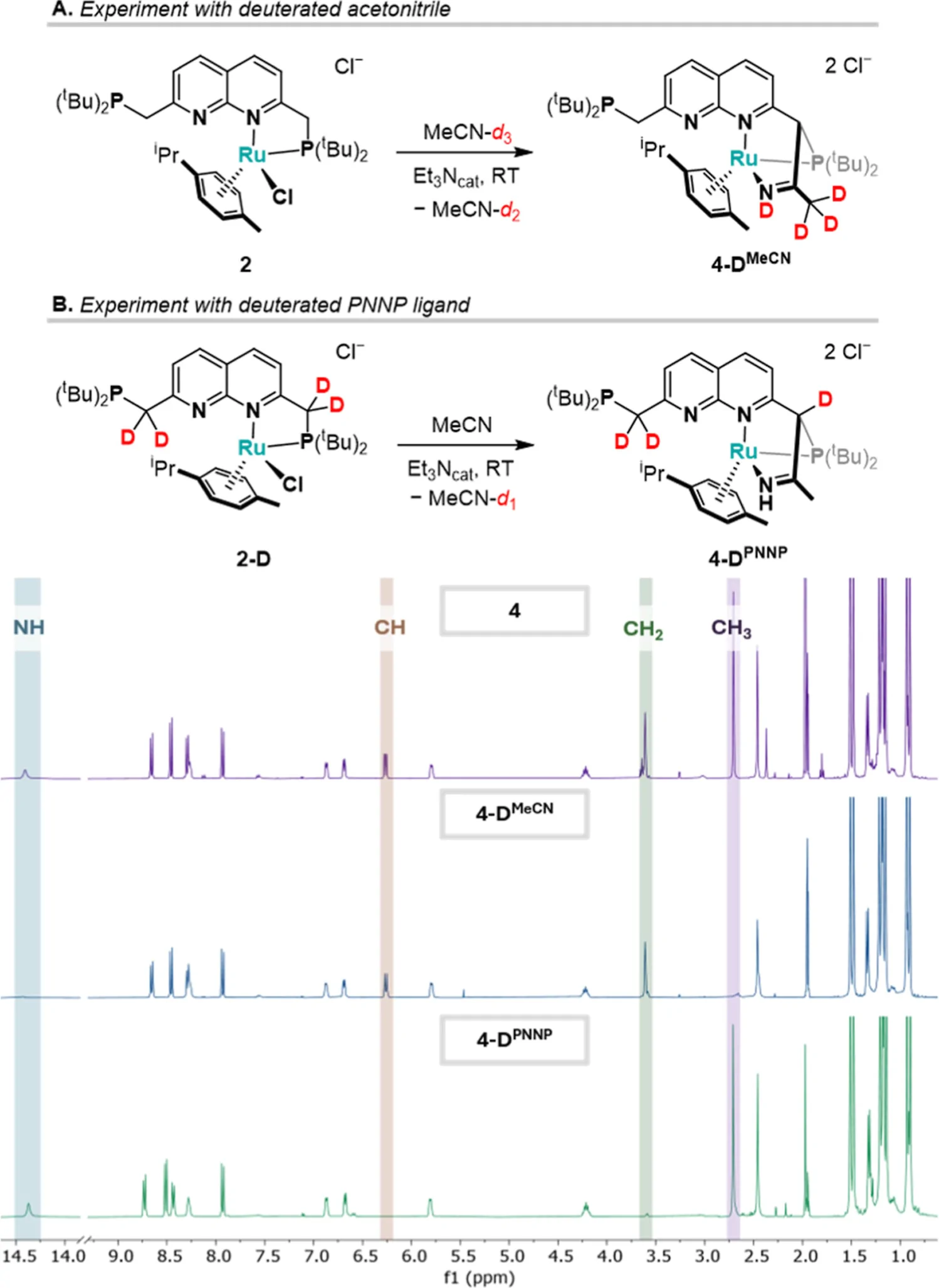
Deuteration experiments. (A) Experiment with deuterated acetonitrile. (B) Experiment with **2-D** (a deuterated analogue of complex **2**) with nondeuterated acetonitrile.

## Computational Investigations

To elucidate the mechanism of acetonitrile activation, density functional theory (DFT) calculations were performed at the BP86-D3BJ/def2-TZVPP//def2-SVP level with SMD (MeCN) implicit solvation at 298.15 K (Figure 5). For computational efficiency, the complex was truncated by removing one phosphine arm that does not participate in metal coordination. Concentration effects arising from the large excess of MeCN relative to the ruthenium complex were also taken into account by applying an approximate experimental concentration ratio of 2600:1 which gives −4.7 kcal mol^–1^.^31,32^ This concentration correction was applied to all the steps involving the MeCN molecule. The dashed line in Figure 5 represents the concentration-corrected reaction pathway. In contrast, the ratio of Et_3_N to the ruthenium complex is only approximately 1:10. As a result, the corresponding concentration correction to the Gibbs free energy is small and falls within the intrinsic error of DFT, and is therefore not considered significant. Hence, we propose that the reaction pathway begins with deprotonation of the PNN–RuCl­(*p*-cymene)­Cl complex (**A1**) by Et_3_N, yielding complex **A2** with a free-energy difference of +7.9 kcal mol^–1^ via transition state **A1-TS** (19.6 kcal mol^–1^), which can be easily overcome at RT. Subsequent chloride dissociation enables coordination of acetonitrile to the ruthenium center, forming intermediate **A3** at 11.3 kcal mol^–1^ relative to **A1**. Coordination of MeCN significantly enhances the electrophilicity of the nitrile carbon, thereby making it amenable for a nucleophilic attack. The rate-determining step of the equimolar free-energy profile involves intramolecular nucleophilic attack of the methine carbon at the coordinated acetonitrile. This step proceeds via transition state **A3-TS** with a barrier of 21.5 kcal mol^–1^ (with respect to **A1**) and leads to formation of intermediate **A4**, a ketimido species, which lies at 3.9 kcal mol^–1^. The relatively low barrier associated with this step indicates that C–C bond formation via metal–ligand cooperation is feasible under mild conditions, in line with experimental observations. Protonation of the ketimido nitrogen by Et_3_NH^+^ then furnishes the final ketimine product **A5** (−1.3 kcal mol^–1^) via transition state **A4-TS** (16.7 kcal mol^–1^). This step completes the formal activation of acetonitrile through metal–ligand cooperation with help of the catalytic amount of an external base.

**Figure 5:**
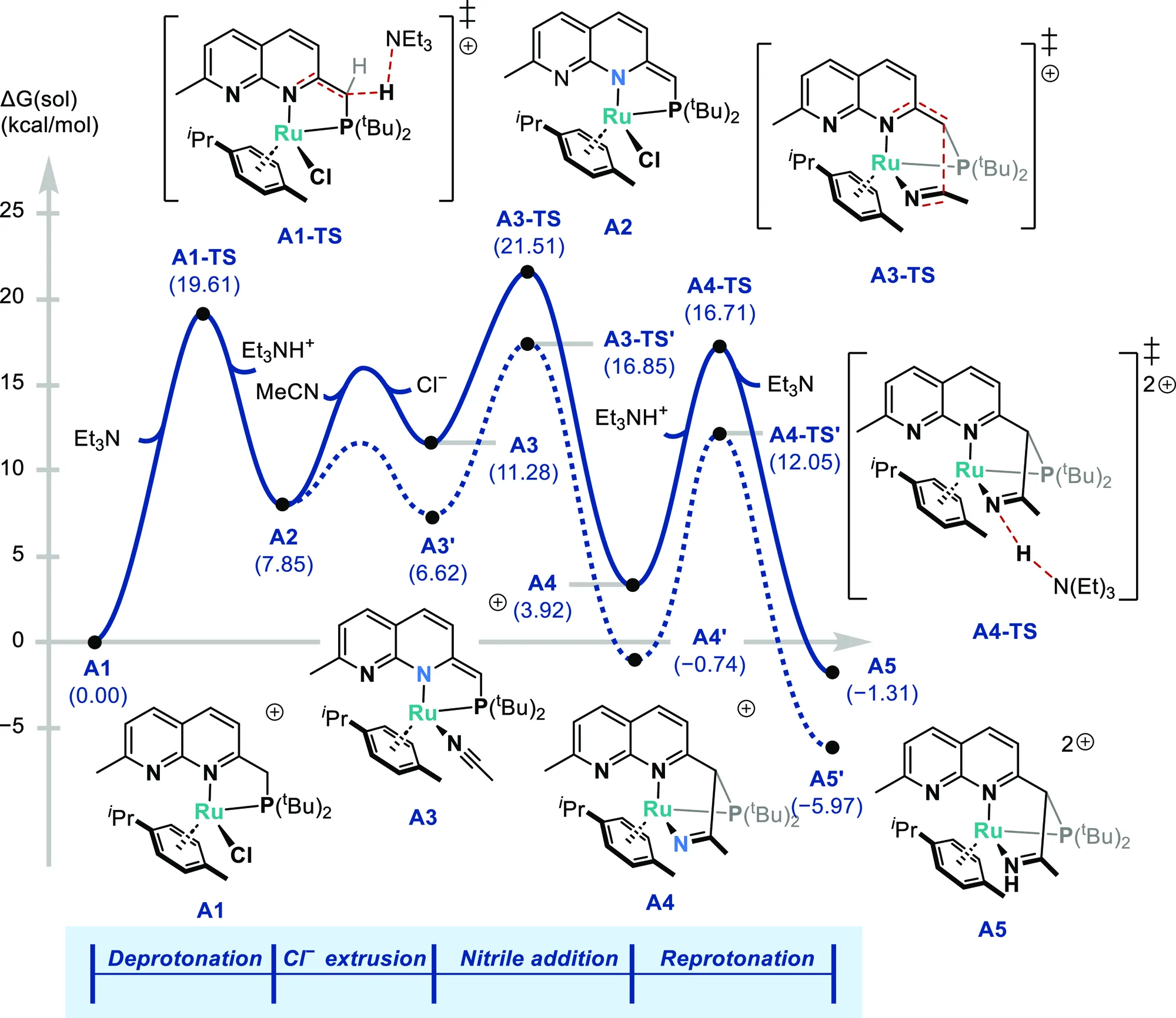
Proposed mechanism of the acetonitrile activation (computed at the BP86-D3BJ/def2-TZVPP//def2-SVP level with SMD (MeCN) implicit solvation at 298.15 K). The complex was truncated, removing one of the phosphine sides that does not participate in coordination to the metal. The dashed line incorporates the experimental concentration ratio between the complex and MeCN solvent (∼1:2600).^31,32^

Upon applying concentration corrections to the equimolar Δ*G*° values discussed above, the free energies of all species from **A3** onward are uniformly lowered by 4.7 kcal mol^–1^. As a consequence, transition state **A1-TS** becomes the overall rate-determining step of the reaction, and the final product **A5** is stabilized to −6.0 kcal mol^–1^ relative to starting complex **A1**. Overall, the computed free-energy barriers along this pathway are consistent with the observed reactivity at room temperature. These results support a mechanism in which acetonitrile activation proceeds through cooperative ligand participation, nucleophilic C–C bond formation, and reprotonation by triethylammonium present in solution. We also assessed a pathway where chloride anion in complex **A1** gets substituted by a MeCN molecule, followed by deprotonation (Figure S70). However, it was found that the deprotonation barrier is overall higher than 28 kcal mol^–1^ making it inconsistent with the observed experimental rates.

We further sought to address other possible mechanistic origins of the NH proton in the ketimine product **A5**, specifically how the α-protons of acetonitrile can ultimately appear on the ketimino nitrogen, as observed in the isotopic labeling experiments. In this context, Milstein and co-workers have previously proposed that ketimido species may reversibly associate with an additional acetonitrile molecule in solution via intramolecular C–H···N contacts.^10^ Consistent with this hypothesis, our calculations (see Figure S72 and the corresponding discussion in the Supporting Information) show that this is also feasible.

An alternative explanation for the observed deuteration of the NH fragment when the reaction is performed in MeCN-*d*_3_ involves direct deprotonation of coordinated acetonitrile from a hypothetical PNN–Ru­(MeCN-*d*_3_)­(*p*-cymene)^+^ complex. Using the isodesmic, 1,8-diazabicyclo[5.4.0]­undec-7-ene (DBU) referenced computational protocol^33^ that we have previously employed for related systems,^20^ the acidity of Ru-bound acetonitrile was estimated to correspond to a p*K*_a_ value of 29.9 (Figure S71). This value is similar to the calculated p*K*_a_ value of the methylene linkers in **A1**, and fully consistent with prior reports describing the facile deprotonation of acetonitrile upon coordination to ruthenium.^34^

We next sought to understand why the 1,8-naphthyridine-based PNNP ligand is more prone to this reaction than the corresponding pyridine-based PN ligand. Our computational data show that the rate-determining step of the overall process is the initial deprotonation of the ligand’s methylene linker. Given that charge is more effectively stabilized in larger π-conjugated systems,^35^ we computationally investigated how the acidity of the methylene moiety changes upon going from the larger 1,8-naphthyridine scaffold to the smaller pyridine analogue (Figure S71). The calculated p*K*_a_ values show that the methylene position in the [PNN–RuCl­(*p*-cymene)] cation is significantly more acidic (p*K*_a_ = 28.2) than in the analogous [PN–RuCl­(*p*-cymene)­Cl] cation (p*K*_a_ = 33.2), providing a straightforward rationale for the observed differences in reactivity between the two complexes.

## Double Activation of Acetonitrile

The PNNP “expanded pincer” ligand exhibits a structural and electronic flexibility that distinguishes it from conventional pincer ligands, such as the higher acidity of the methylene positions discussed above. Moreover, the 1,8-naphthyridine core enables sequential dearomatization at two sites, allowing the ligand to undergo double deprotonation.^18,19^ This feature raised the intriguing possibility that a single PNNP scaffold might engage in two independent metal–ligand cooperative events, thereby enabling the simultaneous activation of two nitrile substrates within the same coordination environment. To examine this hypothesis, we explored the reactivity of two ruthenium precursors, RuCl_2_(DMSO)_4_ and Ru_2_Cl_4_(benzene)_2_, with two equivalents of the PNNP ligand in acetonitrile. Initial experiments resulted exclusively in complex mixtures containing mono- and bi-nuclear species. However, we found that the addition of 2 equiv of KPF_6_ and heating the reaction mixture at 115 °C for 36 h led to the formation of a relatively clean mixture of only two species (Scheme 3), irrespective of the ruthenium precursor used. ^1^H NMR analysis of the resulting products in MeCN-*d*_3_ reveals two sets of resonances in an approximate 20:80 ratio. Each species exhibits a broadened singlet at δ = 10.63 or 10.74 ppm, which is characteristic of NH ketimine moieties formed upon nitrile activation via metal–ligand cooperation.^9,12^ These resonances are located at considerably lower frequencies compared to complexes **3** and **4** and are more consistent with the literature reports where similar NH protons are usually found at δ ∼ 10.00 ppm.^9,12^ We link this to the absence of the outer-sphere chloride anions in case of complex **5** which we hypothesize to interact with **3** and **4** via hydrogen bonding. In addition, both species display two doublets in the aromatic region as well as a distinct doublet at δ = 5.70 or 5.80 ppm. Importantly, all three signals integrating as 2H, strongly suggest that both complexes possess at least *C*_2_ or *C*_*s*_ symmetry and contain two equivalent activated nitrile fragments. This assignment is further corroborated by the ^31^P­{^1^H} NMR spectrum, which shows two singlets at δ = 150.0 and 155.0 ppm in the same 20:80 ratio. The observation of only a single phosphorus resonance per species is fully consistent with a symmetric coordination environment around the ruthenium center in both complexes. The IR spectra of the product mixture display a characteristic NH signal at 3303 cm^–1^ (Figure S48). This together with the found NH resonances at δ = 10.74 ppm in the ^1^H NMR is consistent with our hypothesis on the hydrogen bonding with the out-of-sphere chloride anions in compounds **3** and **4**.

**Scheme 3:**
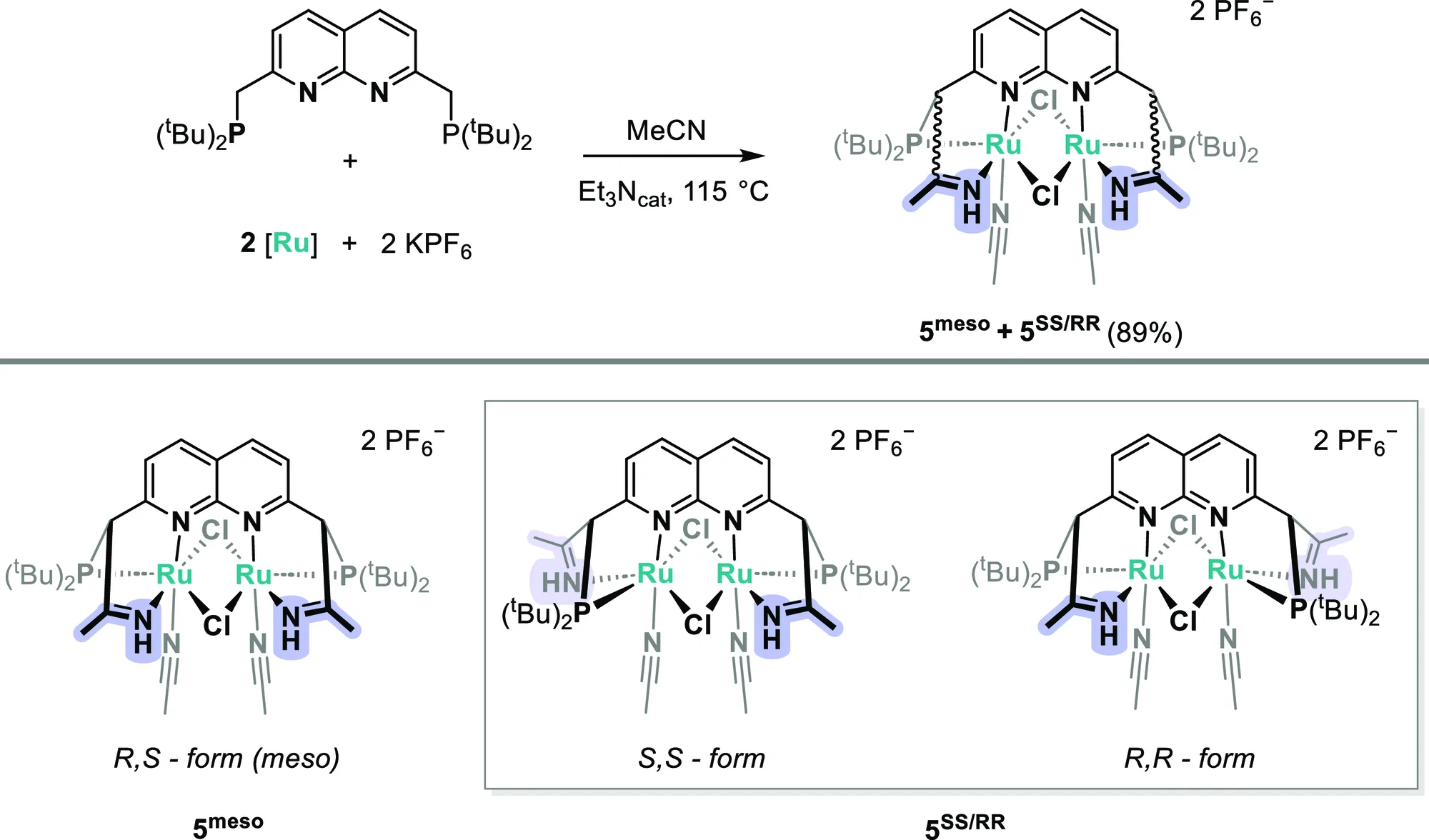
Activation of Two MeCN Molecules Using PNNP Ligand Giving a Mixture of Diastereomers **5** ^***meso***^, **5** ^***SS*/*RR***^

Taken together, these spectroscopic features suggest that the observed species arise from double MLC-induced nitrile activation within a single PNNP framework. The symmetric nature of the complexes and the presence of two NH ketimine functionalities per molecule as well as only slight deviations between the resonances point toward the formation of a mixture of all possible diastereomers, namely: **5** ^***SS***^, **5** ^***RR***^, and **5** ^***meso***^. In the enantiomeric pair, **5** ^***SS***^ and **5** ^***RR***^, the two activated acetonitrile units adopt an anti-arrangement relative to the 1,8-naphthyridine core, whereas in the *meso*-isomer both ketimine moieties are oriented on the same face of the PNNP ligand backbone. The crystals of the **5** ^***meso***^ complex were obtained from a saturated THF solution. Unfortunately, the resulting crystal structure does not meet publishable quality standards; nevertheless, the proposed connectivity inferred from the refined atomic positions is consistent with our structural hypothesis derived from spectroscopic analysis. The preliminary X-ray data indicate that both MeCN fragments are located on the same side of the complex and that each forms a new C–C bond between the methylene linker of the PNNP ligand and the corresponding ketimine moiety.

Density functional theory calculations performed at the BP86-D3BJ/def2-TZVPP//def2-SVP level with SMD (MeCN) implicit solvation at 298.15 K indicate that the *meso*-isomer is thermodynamically favored over the **5** ^***SS***^/**5** ^***RR***^ pair by 2.3 kcal mol^–1^ at RT or 2.1 kcal mol^–1^ at 115 °C (Figure S73). This slight energetic difference is consistent with the experimental observation of two species in solution rather than exclusive formation of a single diastereomer. Assuming a Boltzmann distribution at the reaction temperature of 115 °C, the experimentally observed 20:80 ratio corresponds to an estimated free-energy difference of approximately 1.1 kcal mol^–1^, which is slightly smaller than the calculated preference for the *meso*-isomer (ΔΔ*G* = 2.1 kcal mol^–1^). This difference may arise from the inherent limitations of the applied DFT and implicit-solvation methodology, including the treatment of dispersion and specific solvent effects. The calculations should therefore be regarded as qualitatively supporting the preference for the *meso*-isomer. The reasonable agreement between the experimentally derived and computationally predicted values supports the proposed structural assignments and underscores the subtle energetic balance governing the relative stability of these diastereomeric complexes.

## Conclusions

Nitrile activation by ruthenium complexes supported by a 1,8-naphthyridine-based PNNP ligand proceeds through a metal–ligand cooperative pathway that operates without strong-base-induced ligand deprotonation. The higher acidity of the PNNP methylene linkers allows weak bases such as triethylamine to access reactive dearomatized states in an equilibrium at room temperature. Combined experimental and computational data shows that despite the low steady state concentration of the deprotonated complex, acetonitrile activation is possible due to exergonic formation of a ketimine product. Finally, we demonstrate that a bimetallic RuRu complex enables the simultaneous activation of two nitrile molecules, revealing a new reactivity enabled by the naphthyridine architecture.

## Supplementary Material





## Data Availability

The data related to the work described in this paper is available in the Yoda data repository at DOI: 10.24416/UU01-XFAFF9.

## Notes

DFT coordinates (XYZ)
Experimental methods, synthesis and characterization of new compounds, and DFT calculations (PDF)
