# Supplementary material for: Metal–Ligand Cooperative Nitrile Activation Triggered by a Catalytic Amount of Weak Base
Source: Organometallics. 2026 Sep 1;45(17):2095–103. doi: 10.1021/acs.organomet.6c00104 (PMC13580183; doi:10.1021/acs.organomet.6c00104)
Supplement: Supplementary file 1 [file om6c00104_si_002.pdf]

Supplementary Materials

Metal–Ligand Cooperative Nitrile Activation Triggered by a Catalytic Amount of Weak Base

Stanislav Melnikov[1], Arie J. H. Multem[1], Pieter N. Oostenbrug[1], Martin Lutz[2], Daniël L.J. Broere*[1]

[1]Organic Chemistry and Catalysis, Institute for Sustainable and Circular Chemistry, Faculty of Science, Utrecht University, Universiteitsweg 99, 3584 CG Utrecht, The Netherlands

[2]Structural Biochemistry Bijvoet Centre for Biomolecular Research, Faculty of Science, Utrecht University, Universiteitsweg 99, 3584 CG Utrecht, The Netherlands

**Corresponding Author:* d.l.j.broere@uu.nl

Table of Contents

S1. General Considerations 3

S2. Synthesis and Characterization 4

S2.1. [PNNP-RuCl(benzene)] Cl (1) 4

S2.2. [PNNP-RuCl(benzene)] PF_6 (1-PF_6) 9

S2.3. [PNNP-RuCl(*p*-cymene)] Cl (2) 14

S2.4. [$PNNP^{MeCN}$-Ru(benzene)] 2Cl (3) 20

S2.5. [$PNNP^{MeCN}$-Ru(*p*-cymene)] 2Cl (4) 25

S2.6. [$PNNP^{2\text{-}MeCN}$-$Ru_2Cl_2(MeCN)_2$] 2PF_6 (5) 31

3. Isotopic Labelling Experiments 37

S3.1. PNNP-d_4 37

S3.2. [PNNP-RuCl(*p*-cymene)] Cl (2-D) 40

S3.3. [PNNP-d_3^{MeCN}-RuCl(*p*-cymene)] 2Cl (4-D^{PNNP}) 43

S3.4. [$PNNP^{MeCN\text{-}d4}$-RuCl(*p*-cymene)] 2Cl (4-D^{MeCN}) 46

S4. Computational Part 52

S5. X-ray crystal Structures 57

5.1 X-ray crystal structure determination of 1-PF_6 57

5.2 X-ray crystal structure determination of 4 58

5.2 Preliminary X-ray crystal structure analysis of 5^{meso} 59

S5. References 60

S1. General Considerations

All manipulations were performed under inert atmosphere using standard Schlenk techniques or inside of a N_2-filled M. Braun glovebox using dry solvents and reagents, unless stated otherwise. Glassware was dried at 130 °C in an oven or with a heat gun under a dynamic vacuum, unless noted otherwise. Hexane and DCM were collected from an M. Braun MB-SPS-800 solvent purification system and degassed and stored over 4 Å molecular sieves, except for DCM which was dried over 3 Å molecular sieves. MeCN was dried over 3 Å molecular sieves and subsequently filtered over activated alumina. THF was dried over Na/benzophenone ketyl (purple), or over Na dispersed on silica spheres, vacuum-transferred and degassed, subsequently followed by storage over 4 Å molecular sieves. Benzene (Scharlab, > 99%), pentane (technical, VWR chemicals), and MeCN (Sigma Aldrich) were degassed, then dried and stored over 4 Å molecular sieves. All non-deuterated solvents were degassed by bubbling N_2 (g) through the solvent for at least 30 min. The solvents (1.0 mL) were tested with a standard purple solution of sodium benzophenone ketyl in THF to confirm effective oxygen and water removal (max 1-2 drops for most solvents, max 4 drops for THF). All solvents were checked for water content by Karl-Fischer titration and all of them were well below 5 ppm. Deuterated solvents were obtained from Cambridge Isotope Laboratories, degassed by the standard freeze-pump-thaw procedure and stored over 4 Å molecular sieves (3 Å for CD_2Cl_2 and CD_3CN). All commercial reagents were used as received and were obtained from Sigma Aldrich, Acros, Strem. 2-methyl-1,8-naphthyridine was obtained from Betapharma. PNNP was prepared according to literature procedures.[1,2]

NMR measurements

NMR data was recorded on an Agilent MRF400 equipped with a OneNMR probe (with quartz liner) and Optima Tune system or an VNMRS-400 Varian 400 MHz (9.4 T) NMR system equipped with an OneNMR probe (with quartz liner) and Optima Tune system and a Performa IV PFG amplifier capable of generating a 65 G/cm gradient or a Jeol JNM-ECZL G 400 MHz (9.4 T) NMR system equipped with an autotunable ROYALPROBE HFX (with quartz liner) and a gradient amplifier capable of generating a 90 G/cm gradient. All chemical shifts are reported in the standard δ notation of parts per million, referenced to the residual solvent peak. All resonances in ^{1}H NMR and ^{13}C NMR spectra were referenced to residual solvent peaks (^{1}H NMR: 5.32 and 1.94 for CD_2Cl_2 and CD_3CN, respectively; ^{13}C NMR: 53.84 and 1.32 for CD_2Cl_2 and CD_3CN, respectively). The resonances in the ^{31}P NMR spectra are referenced using the absolute reference method from a correctly referenced ^{1}H NMR spectrum of the same sample. The assignment of peaks is based on relative integration, chemical shift, and 2D NMR analysis (COSY, HMBC, HMQC). For ^{1}H NMR spectra in non-deuterated solvents, solvent suppression is used (PRESAT or WET). All NMR experiments involving air-sensitive compounds were conducted in J. Young NMR tubes under a N_2 atmosphere. Peak multiplicity was quoted as s (singlet), d (doublet), t (triplet) and so on.

Other physical methods

ATR-FTIR spectra were recorded on a PerkinElmer Frontier FTIR spectrometer equipped with a PerkinElmer Universal Attenuated Total Reflectance (ATR) Sampling Accessory with a diamond/ZnSe plate and a $LiTaO_3$ mid-IR detector. IR-analysis of air-sensitive compounds was performed by dropcasting a solution (MeCN or DCM) onto the ATR crystal, which was covered by a continues N_2 flow.

S2. Synthesis and Characterization

S2.1. [PNNP-RuCl(benzene)] Cl (1)

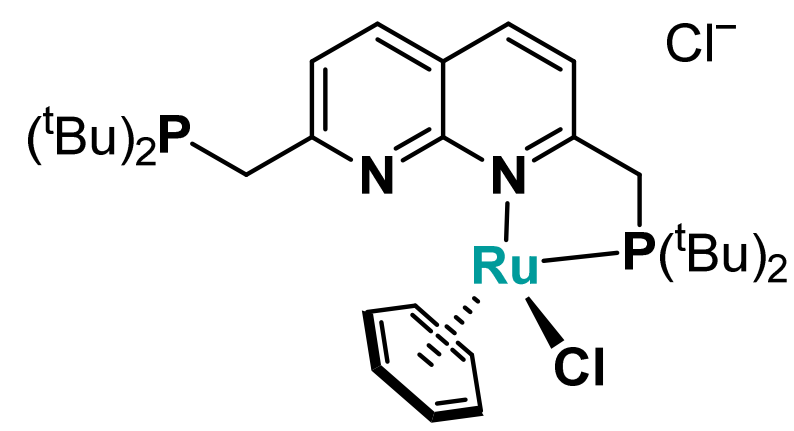

A 100 mL Schlenk tube was charged with ^{tBu}PNNP (223.2 mg, 0.50 mmol), and $[Ru_2Cl_4(C_6H_6)_2]$ (125.0 mg, 0.25 mmol). Next, benzene (10.0 mL) was added to give a dark brown suspension. The reaction mixture was kept stirring in a glovebox at RT for 18 h, during which the color of the reaction mixture became greenish. The mixture was filtered through a glass filter, and the residue was washed with benzene (3 x 5.0 mL). The product was extracted from the residue with 10.0 mL of DCM to give a dark brown solution. All the volatiles were removed under a dynamic vacuum giving 268.1 mg (77%) of orange powder.

^{1}H NMR (400 MHz, CD_2Cl_2, 298 K): δ = 8.45 (dd, $^{3}J_{H,H}$ = 8.2, $^{4}J_{H,H}$ = 1.0 Hz, 1H), 8.33 (d, $^{3}J_{H,H}$ = 8.3 Hz, 1H), 7.85 (dd, $^{3}J_{H,H}$ = 8.4, $^{4}J_{H,H}$ = 0.9 Hz, 1H), 7.79 (d, $^{3}J_{H,H}$ = 8.1 Hz, 1H), 6.54 (s, 6H), 4.29 (dd, $^{2}J_{H,H}$ = 16.9, $^{2}J_{H,P}$ = 8.6 Hz, 1H), 4.03 (dd, $^{2}J_{H,H}$ = 17.0, $^{2}J_{H,P}$ = 13.1 Hz, 1H), 3.52 (d, $^{2}J_{H,P}$ = 2.1 Hz, 2H), 1.64 (d, $^{3}J_{H,P}$ = 14.2 Hz, 9H), 1.25 (d, $^{3}J_{H,P}$ = 13.5 Hz, 9H), 1.23 (d, $^{3}J_{H,P}$ = 11.1 Hz, 9H), 1.15 (d, $^{3}J_{H,P}$ = 11.1 Hz, 9H).

^{13}C{^{1}H} NMR (100 MHz, CD_2Cl_2, 298 K): δ = 170.3 (s), 168.5 (d, $^{2}J_{C,P}$ = 11.4 Hz), 155.0 (s), 141.8 (s), 139.1 (s), 125.7 (d, $^{3}J_{C,P}$ = 7.6 Hz), 122.8 (d, $^{3}J_{C,P}$ = 8.4 Hz), 121.5 (s), 89.7 (d, $^{2}J_{C,P}$ = 2.7 Hz), 39.8 (d, $^{1}J_{C,P}$ = 16.1 Hz), 39.5 (d, $^{1}J_{C,P}$ = 18.0 Hz), 37.1 (d, $^{1}J_{C,P}$ = 23.0 Hz), 33.3 (d, $^{1}J_{C,P}$ = 28.9 Hz), 32.8 (d, $^{2}J_{C,P}$ = 4.8 Hz), 32.6 (d, $^{2}J_{C,P}$ = 4.1 Hz), 31.5 (d, $^{2}J_{C,P}$ = 2.3 Hz), 30.4 (d, $^{2}J_{C,P}$ = 2.6 Hz), 30.0 (s), 29.9 (s).

^{31}P{^{1}H} NMR (162 MHz, CD_2Cl_2, 298 K): δ = 86.4 (s, 1P), 38.2 (s, 1P).

The purity of **1** was confirmed by quantitative ^{1}H NMR analysis using 1,3,5-trimethoxybenzene as an internal standard, indicating high purity with only trace amounts of residual solvent and minor starting-material impurities.

ATR-IR (film, N_2 flow, 298 K): ν = 2935 (s), 2895 (s), 2862 (s), 1730 (m), 1633 (m), 1595 (m), 1538 (m), 1509 (m), 1465 (m), 1419 (m), 1393 (m), 1366 (m), 1294 (w), 1261 (w), 1218 (w), 1180 (m), 1138 (m), 1018 (w), 809 (w), 677 (w), 642 (w) cm^{-1}.

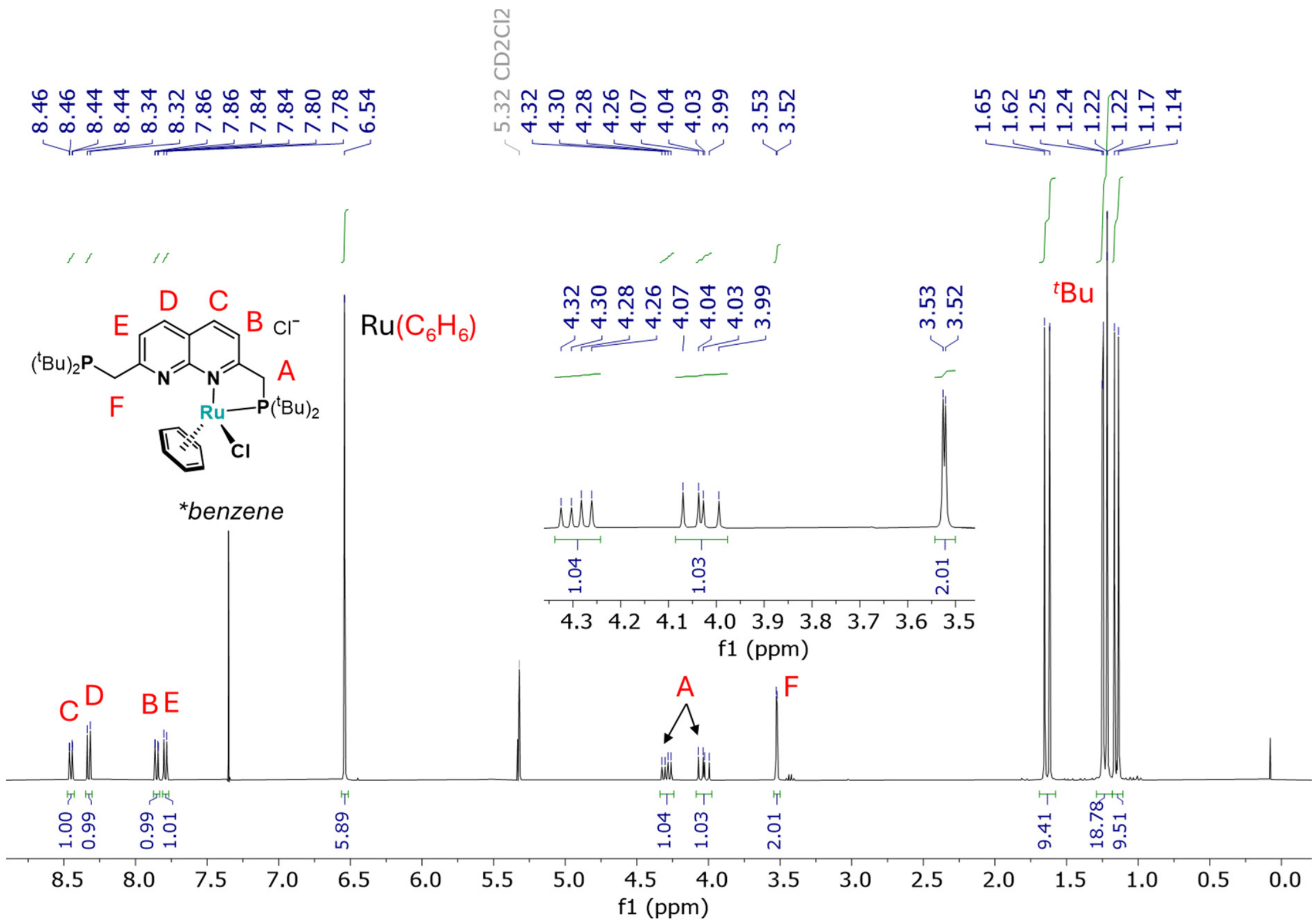

Figure S1. ^{1}H NMR spectrum of **1** in CD_2Cl_2 at 298 K.

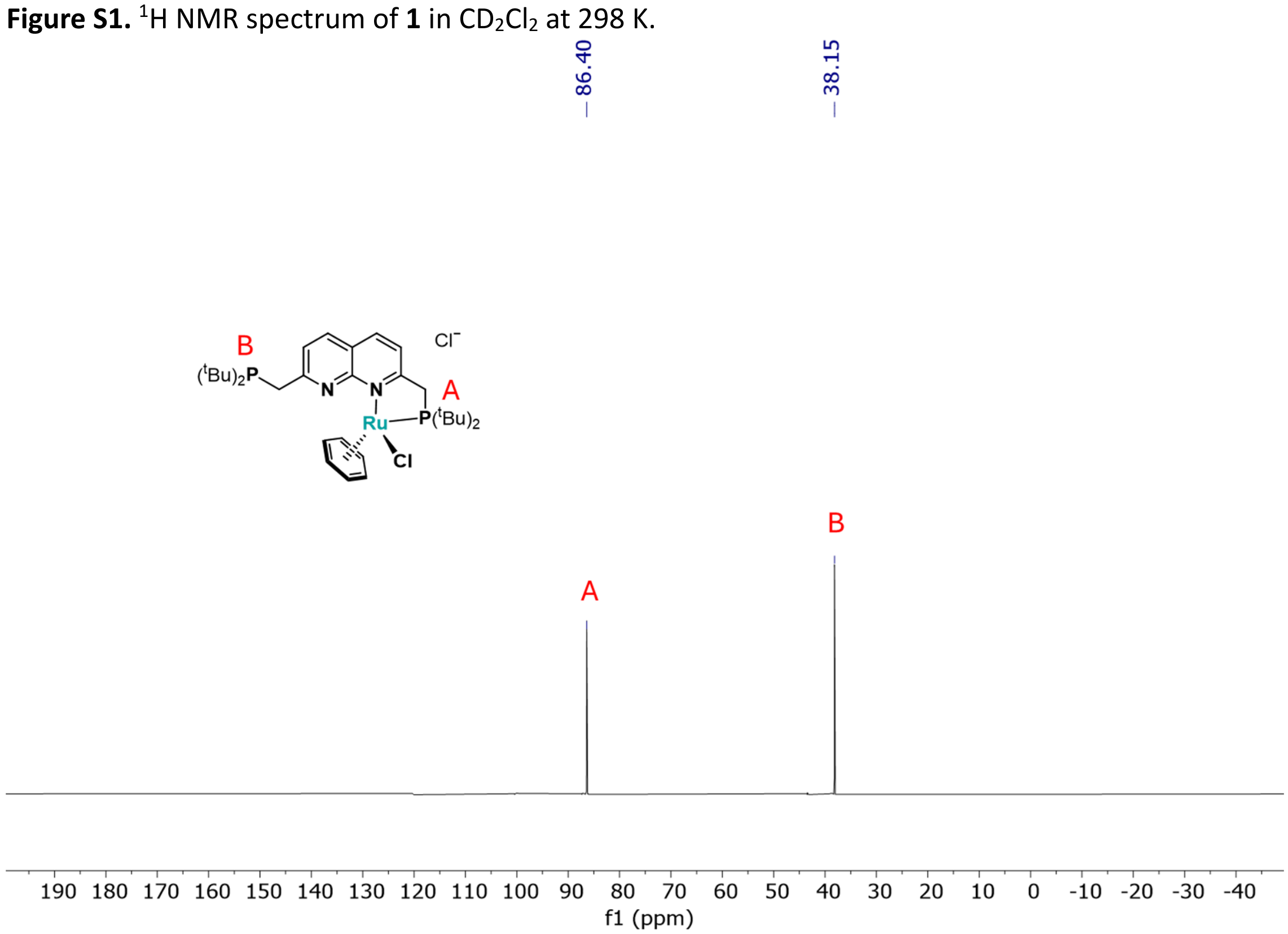

Figure S2. $^{31}P\{^{1}H\}$ NMR spectrum of **1** in CD_2Cl_2 at 298 K.

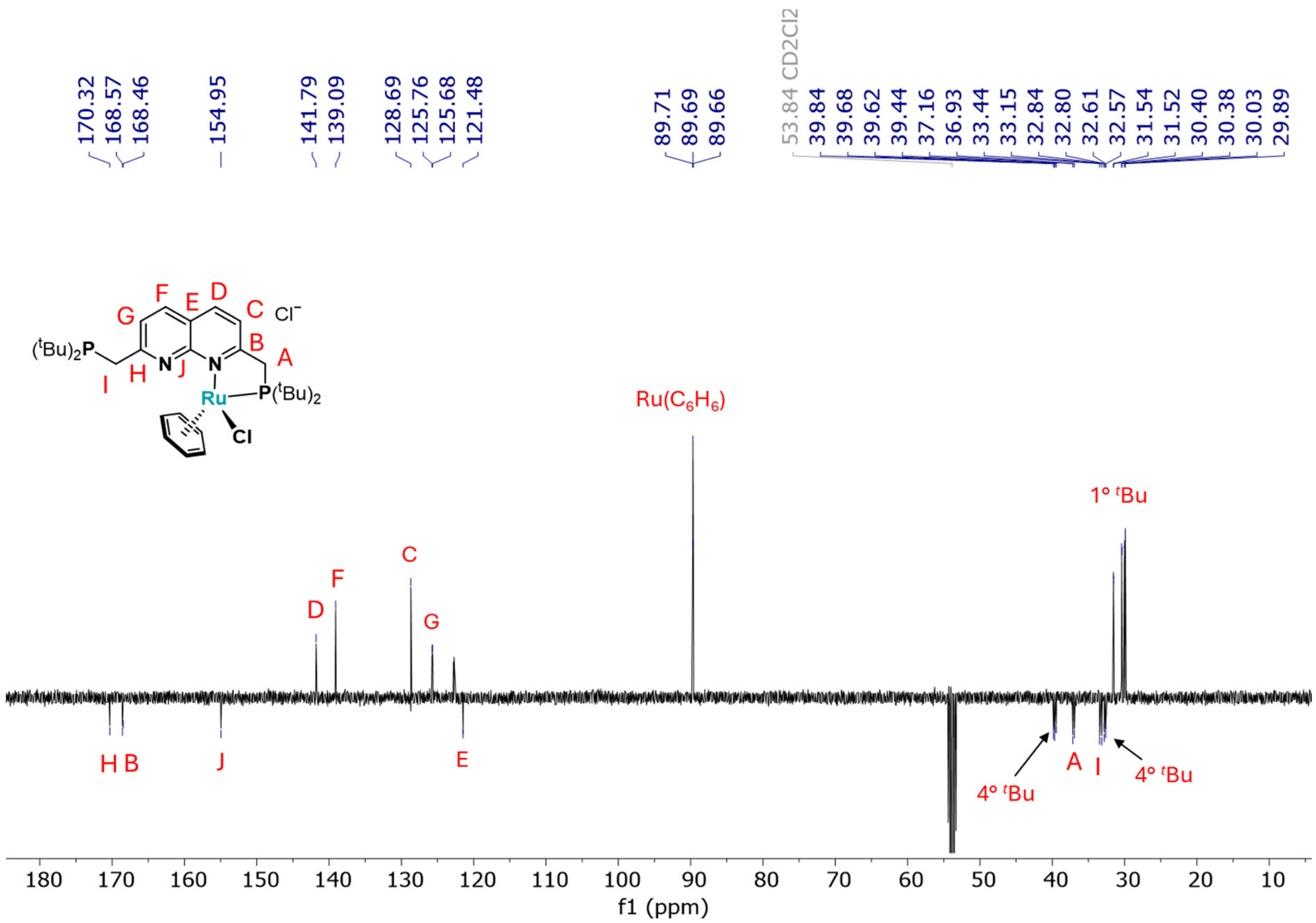

Figure S3. $^{13}C\{^{1}H\}$ APT NMR spectrum of **1** in CD_2Cl_2 at 298 K.

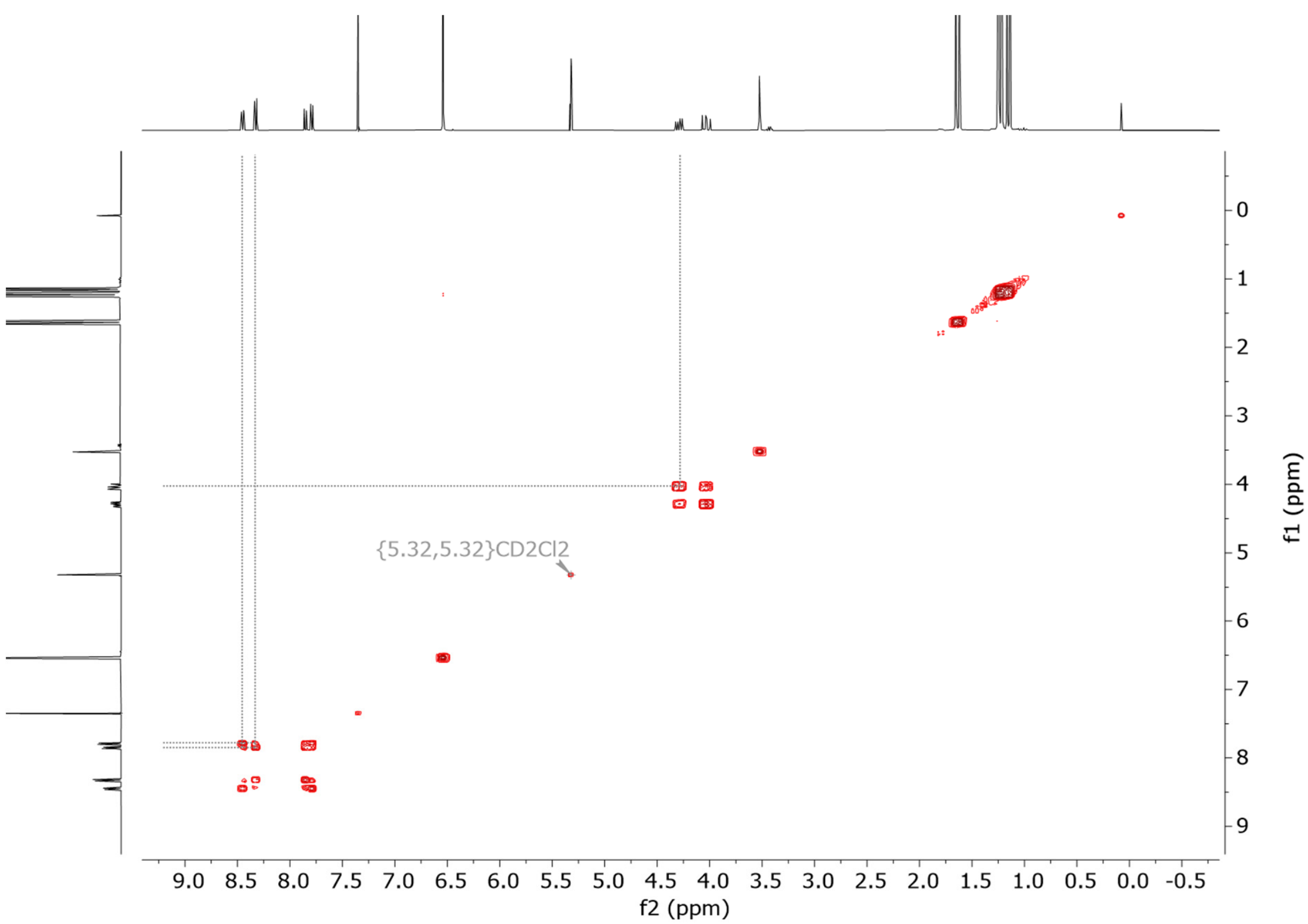

Figure S4. ^{1}H-^{1}H COSY NMR spectrum of **1** in CD_2Cl_2 at 298 K.

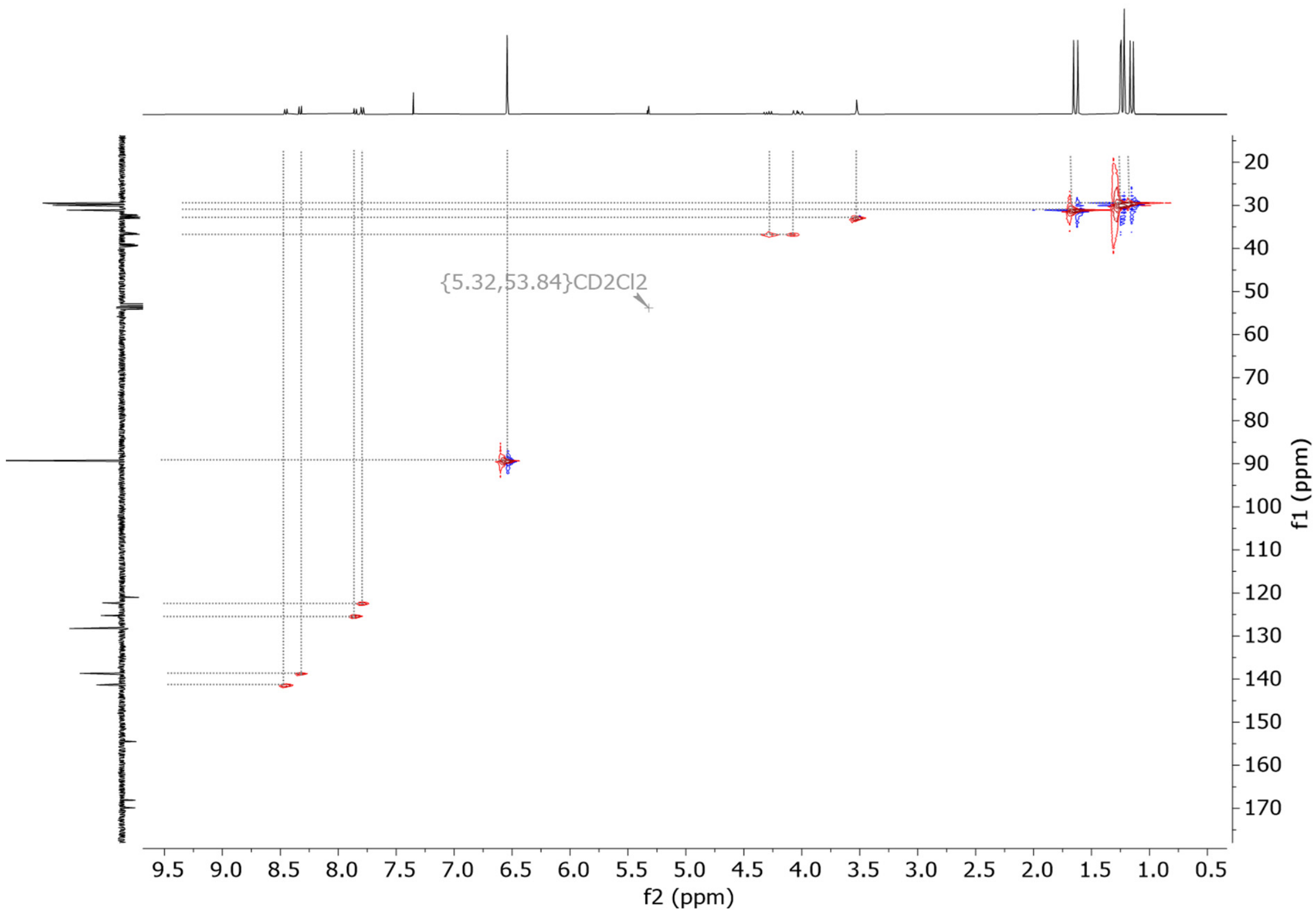

Figure S5. ^{1}H-^{13}C ASAP-HMQC NMR spectrum of **1** in CD_2Cl_2 at 298 K.

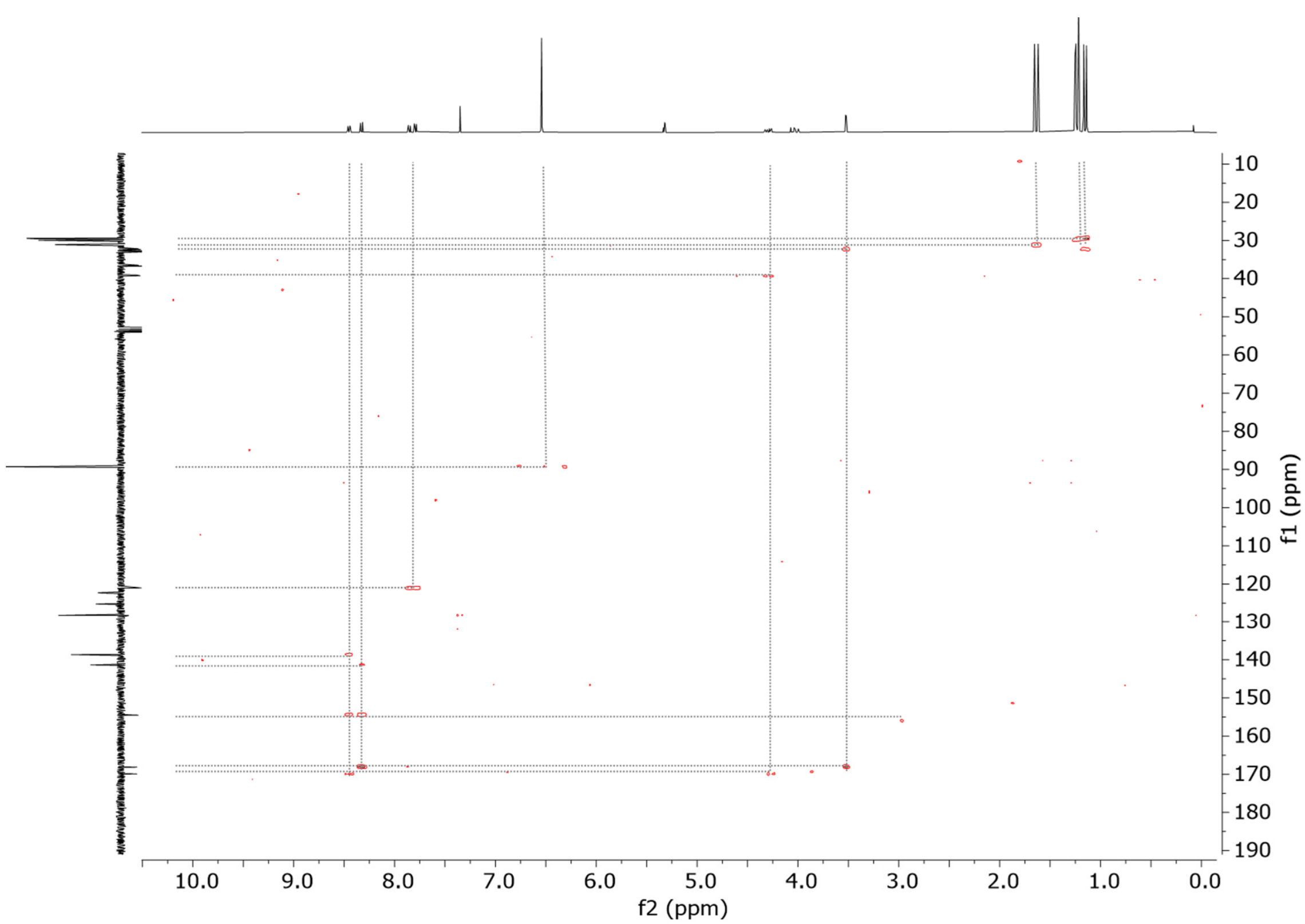

Figure S6. ^{1}H-^{13}C HMBC NMR spectrum of **1** in CD_2Cl_2 at 298 K.

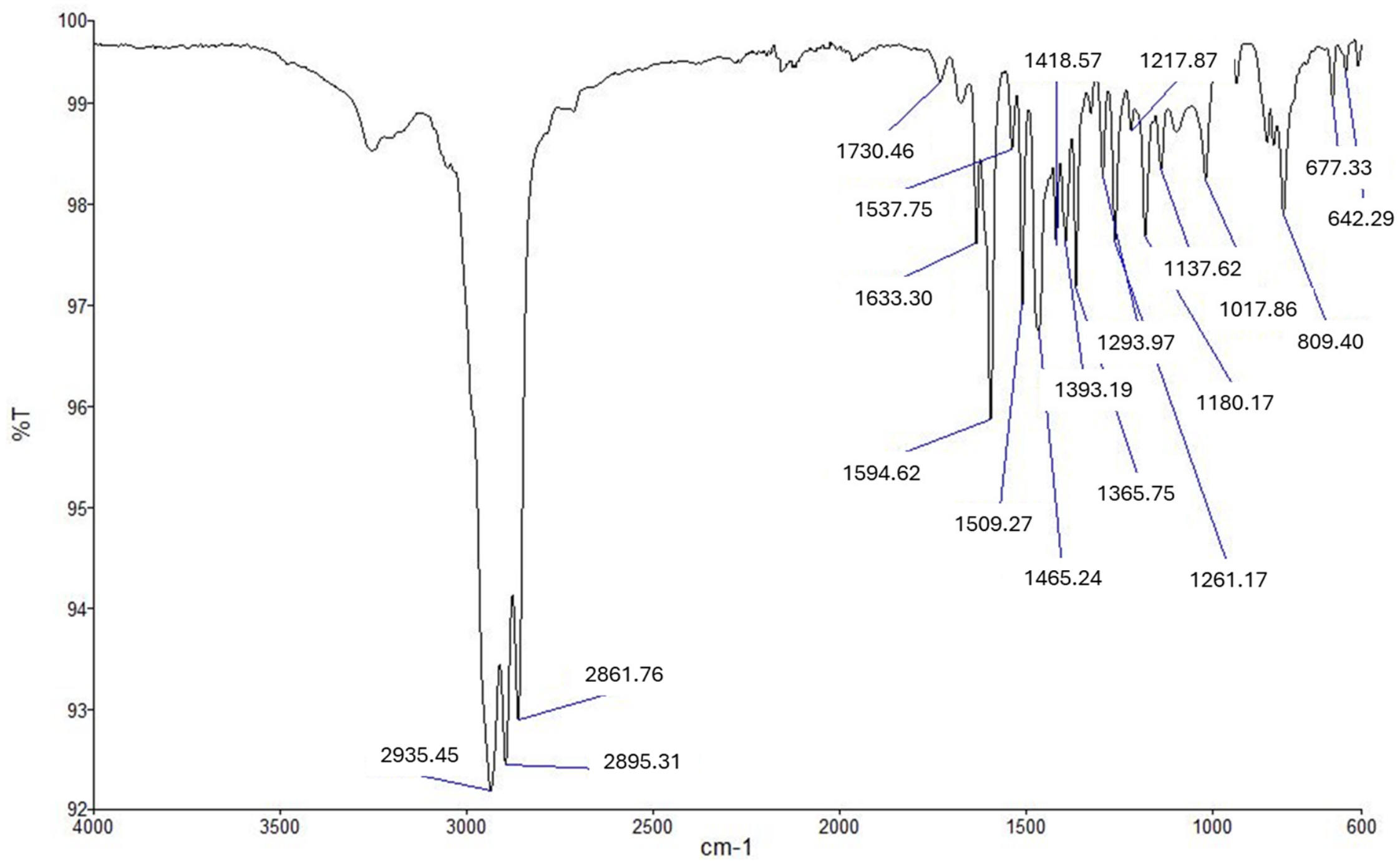

Figure S7. ATR-IR spectrum of **1** measured as a film under N_2 flow at 298 K.

S2.2. [PNNP-RuCl(benzene)] PF_6 (1-PF_6)

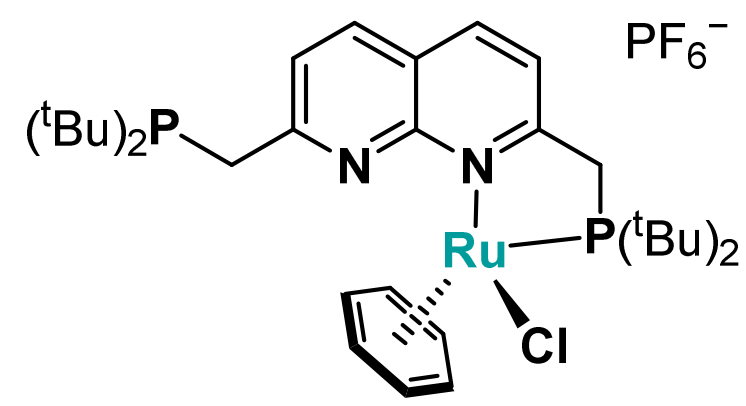

A 20 mL glass vial was charged with **1** (34.8 mg, 0.05 mmol), and DCM (5.0 mL) to give an orange solution. Next, KPF_6 (13.8 mg, 0.08 mmol, excess) was added, and the reaction mixture was stirred in a glovebox at room temperature for 18 h. The mixture was then filtered through a glass fiber filter to remove remaining KPF_6 and KCl, affording an orange solution. All volatiles were removed under dynamic vacuum yielding 37.1 mg (92%) of product as an orange powder. Crystals suitable for X-ray diffraction analysis were grown by vapor diffusion of Et_2O into a saturated solution of **1-PF_6** in DCM at room temperature.

^{1}H NMR (400 MHz, CD_2Cl_2, 298 K): δ = 8.30 (d, $^3J_{H,H}$ = 8.1 Hz, 1H), 8.22 (d, $^3J_{H,H}$ = 8.3 Hz, 1H), 7.87 (d, $^3J_{H,H}$ = 8.7 Hz, 1H), 7.57 (d, $^3J_{H,H}$ = 8.1 Hz, 1H), 6.49 (s, 6H), 4.33 (dd, $^3J_{H,H}$ = 16.9, $^2J_{H,P}$ = 8.6 Hz, 1H), 3.72 (dd, $^3J_{H,H}$ = 17.0, $^2J_{H,P}$ = 13.0 Hz, 1H), 3.53 (d, $^2J_{H,P}$ = 2.1 Hz, 2H), 1.63 (d, $^3J_{H,P}$ = 14.3 Hz, 9H), 1.24 (d, $^3J_{H,P}$ = 9.5 Hz, 9H), 1.21 (d, $^3J_{H,P}$ = 11.8 Hz, 9H), 1.16 (d, $^3J_{H,P}$ = 11.1 Hz, 9H).

^{13}C{^{1}H} NMR (100 MHz, CD_2Cl_2, 298 K): δ = 169.9, 168.9 (d, *J* = 11.5 Hz), 155.0, 141.7, 138.9, 125.9 (d, *J* = 7.7 Hz), 122.3 (d, *J* = 8.4 Hz), 121.4, 89.7, 39.8 (d, *J* = 6.5 Hz), 39.6 (d, *J* = 8.4 Hz), 37.0 (d, *J* = 22.9 Hz), 33.3 (d, *J* = 28.9 Hz), 32.9 (d, *J* = 4.3 Hz), 32.6 (d, *J* = 3.8 Hz), 31.4 (d, *J* = 2.3 Hz), 30.2 (d, *J* = 2.5 Hz), 30.0 (d, *J* = 1.5 Hz), 29.9 (d, *J* = 1.6 Hz).

^{31}P{^{1}H} NMR (162 MHz, CD_2Cl_2, 298 K): δ = 85.8 (s, 1P), 38.4 (s, 1P), –144.5 (hept, $^1J_{P,F}$ = 710.8 Hz, 1P).

^{19}F NMR (376 MHz, CD_2Cl_2, 298K): δ = –73.04 (d, $^1J_{F,P}$ = 710.7 Hz).

*The ^{1}H NMR spectrum of **1-PF_6** displays the same set of resonances as observed for its chloride analogue **1**. However, the signals are noticeably shifted, suggesting a potential tight ion-pair character for compound **1**. In contrast, the ^{13}C and ^{31}P NMR spectra show chemical shifts that are much more similar between **1-PF_6** and **1**.*

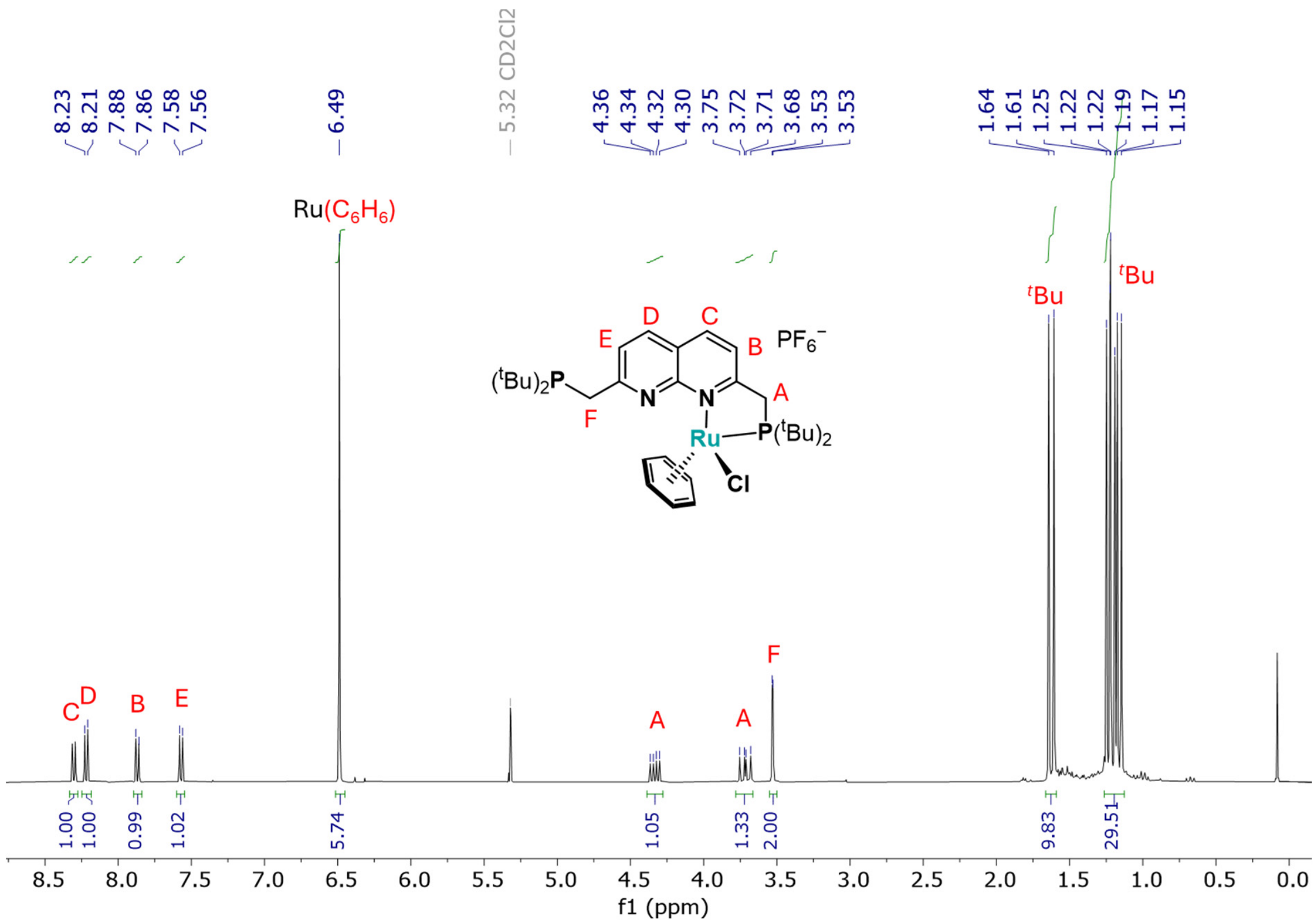

Figure S8. ^{1}H NMR spectrum of **1-PF$_6$** in CD_2Cl_2 at 298 K

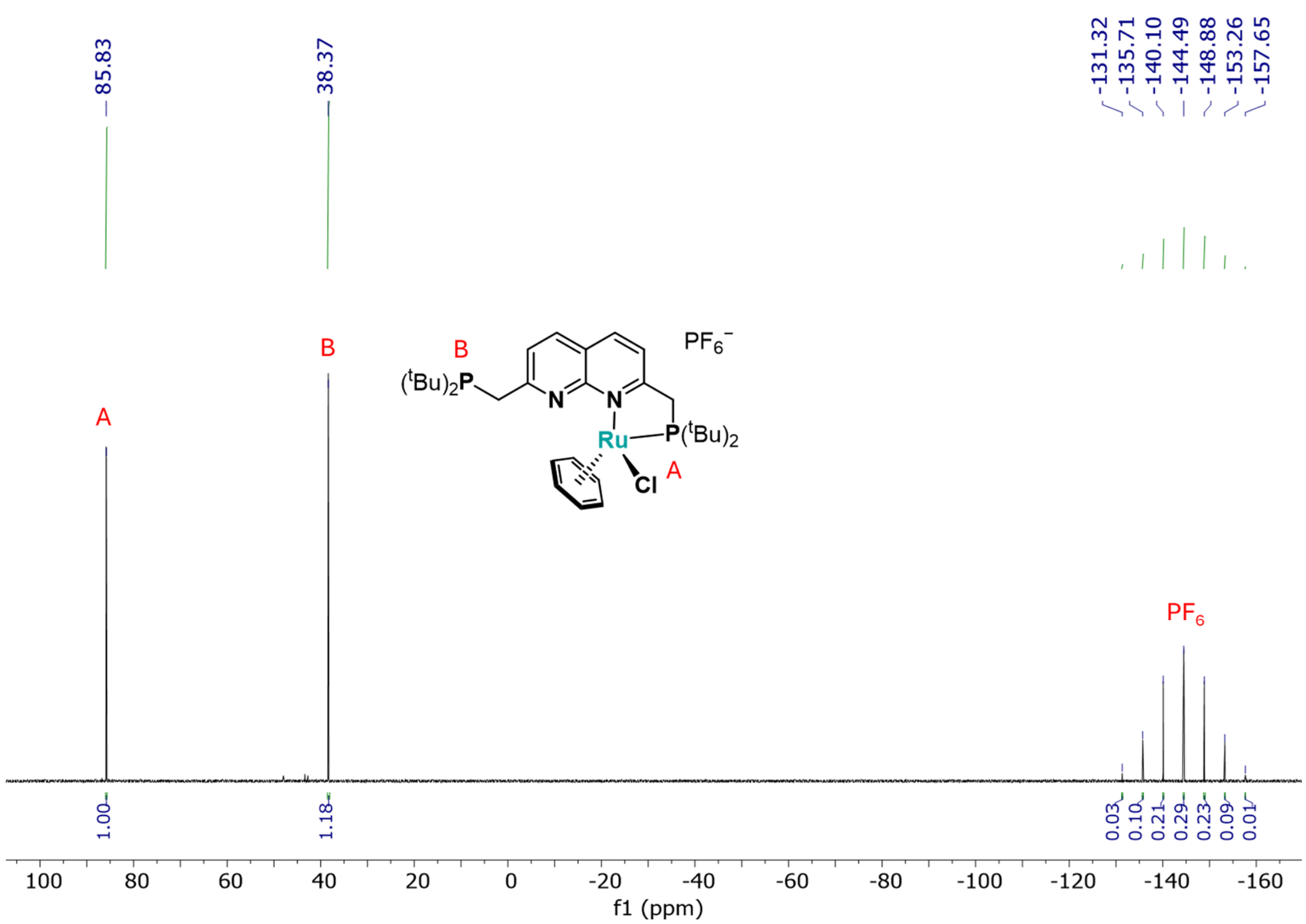

Figure S9. ^{31}P{^{1}H} NMR spectrum of **1-PF$_6$** in CD_2Cl_2 at 298 K

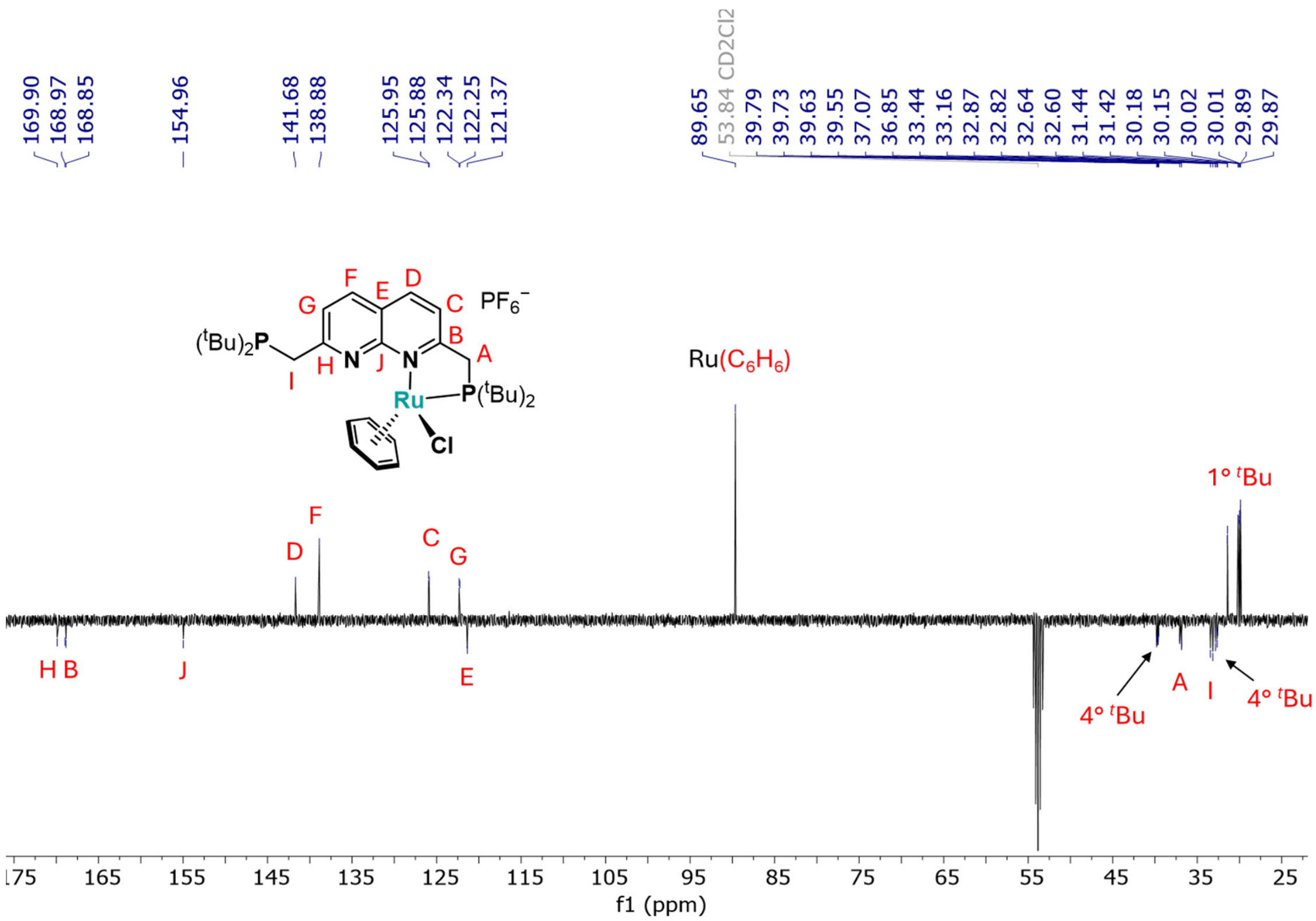

Figure S10. $^{13}C\{^{1}H\}$ APT NMR spectrum of **1-PF$_6$** in CD_2Cl_2 at 298 K

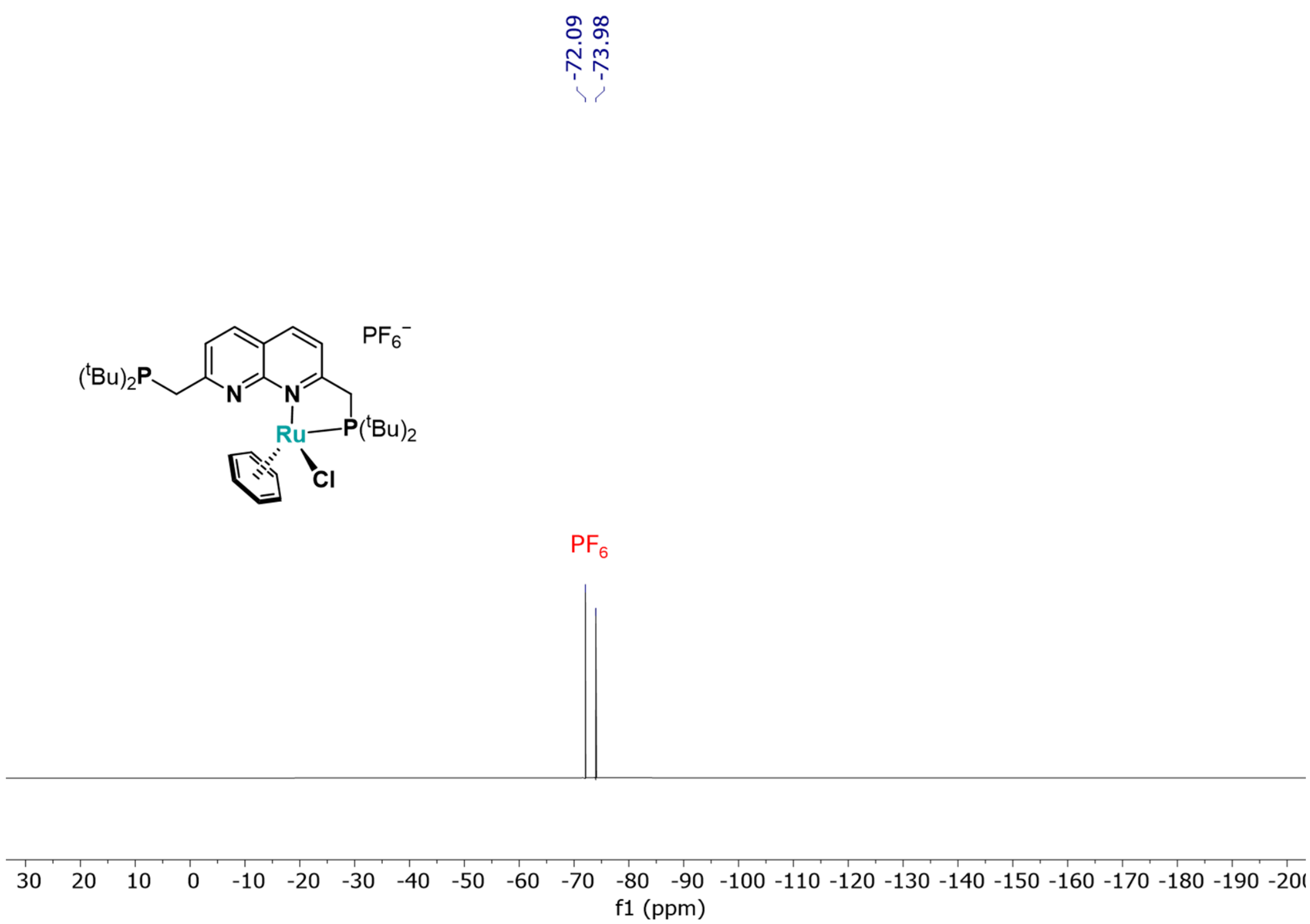

Figure S11. ^{19}F NMR spectrum of **1-PF$_6$** in CD_2Cl_2 at 298 K

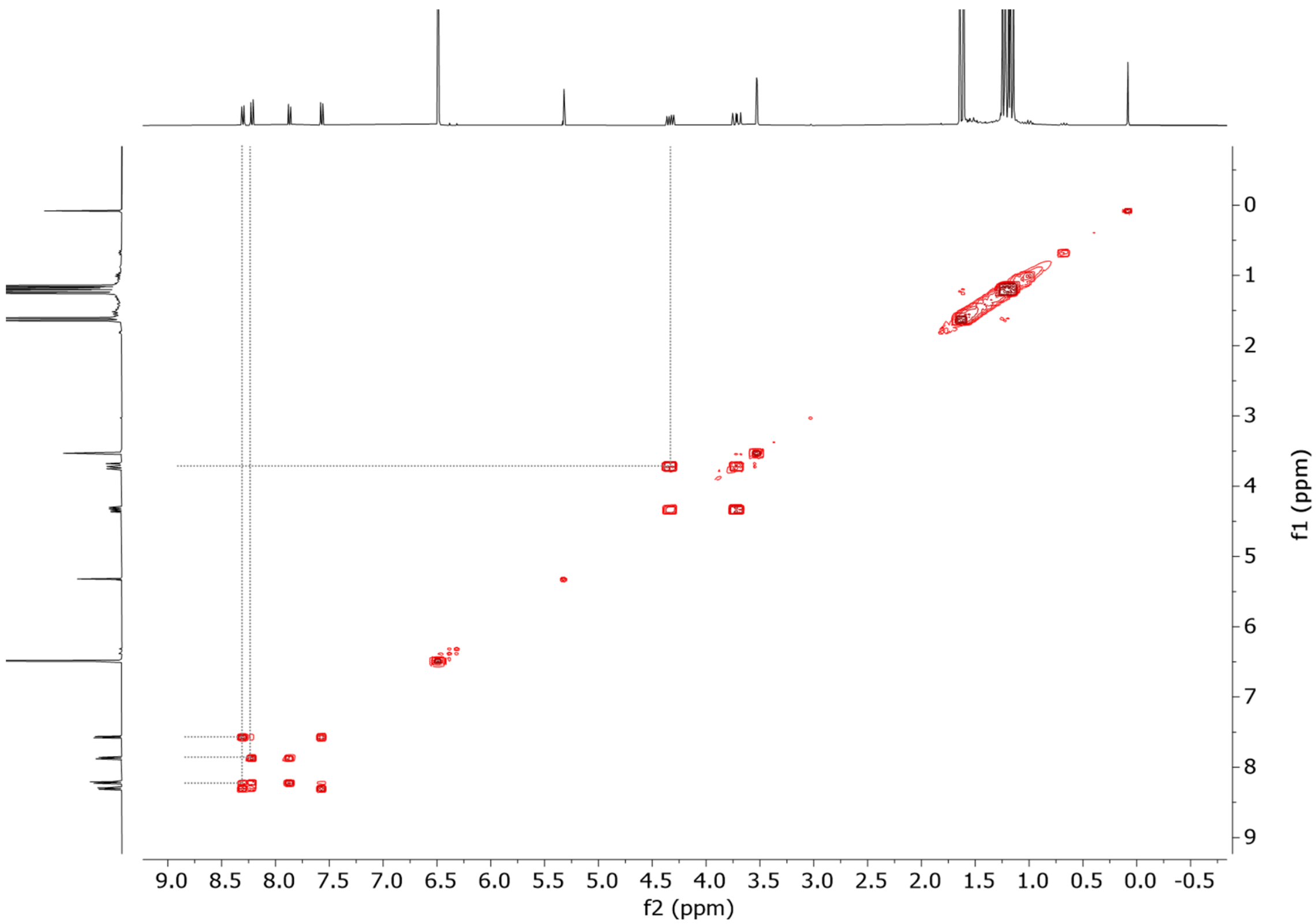

Figure S12. ^{1}H-^{1}H COSY NMR spectrum of **1-PF$_6$** in CD_2Cl_2 at 298 K

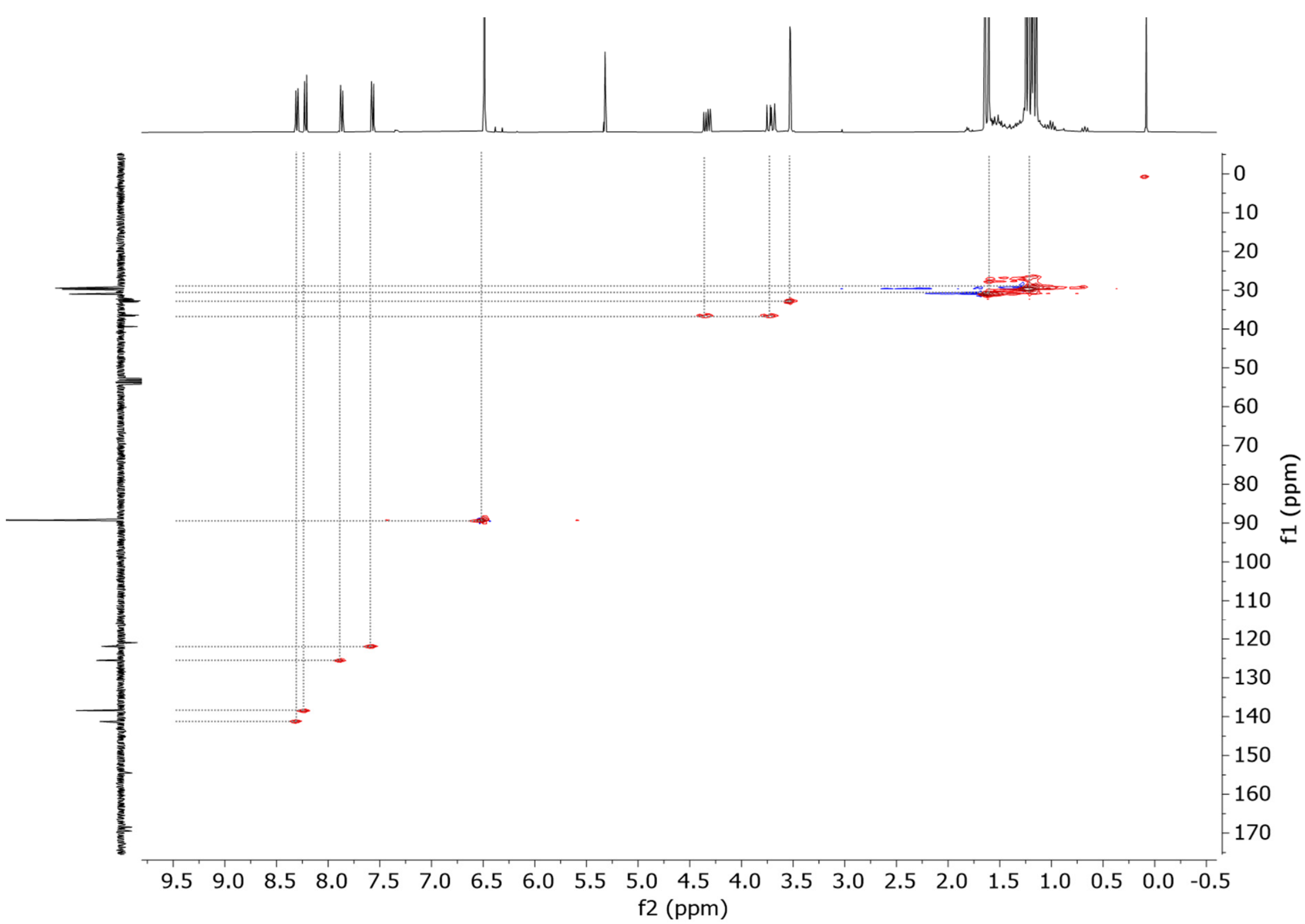

Figure S13. ^{1}H-^{13}C ASAP-HMQC NMR spectrum of **1-PF$_6$** in CD_2Cl_2 at 298 K

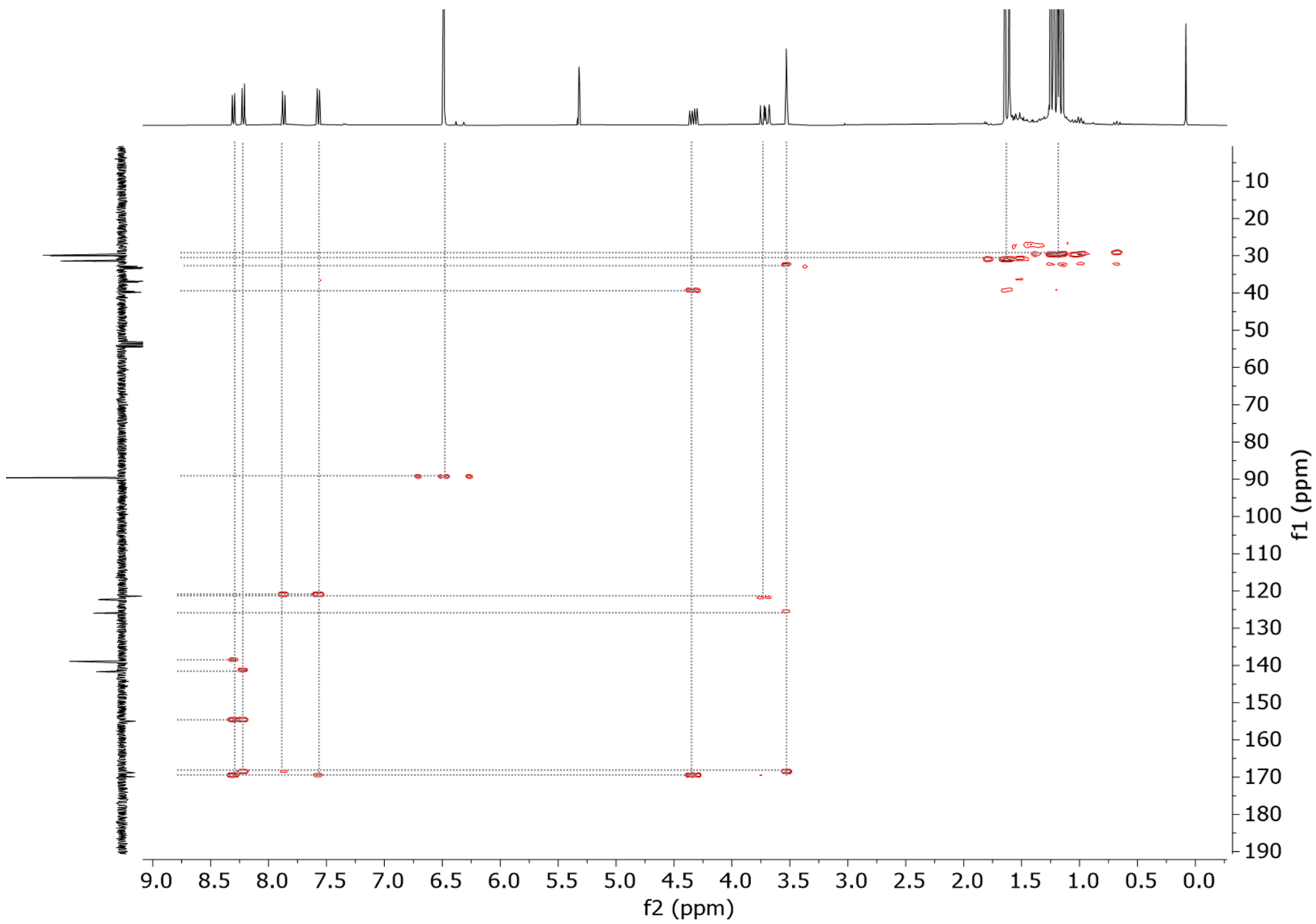

Figure S14. ^{1}H-^{13}C HMBC NMR spectrum of **1-PF₆** in CD_2Cl_2 at 298 K

S2.3. [PNNP-RuCl(*p*-cymene)] Cl (2)

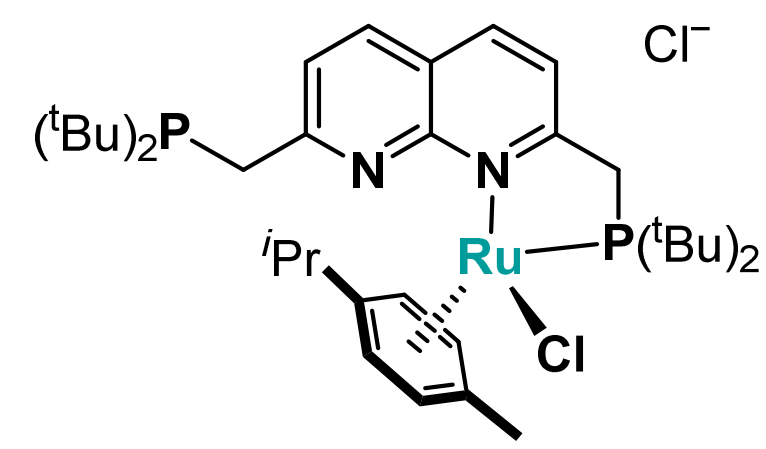

A 20 mL glass vial was charged with ^{tBu}PNNP (89.3 mg, 0.20 mmol), and [Ru_2Cl_4(*p*-cymene)$_2$] (61.2 mg, 0.10 mmol). Next, DCM (10.0 mL) was added to give a clear red solution. The reaction mixture was kept stirring in a glovebox at RT for 2 h, during which the color of the reaction mixture became dark red. The mixture was filtered through a glass filter to give a red solution. All the volatiles were removed under a dynamic vacuum giving 134.0 mg (89%) of red powder.

^{1}H NMR (400 MHz, CD_2Cl_2, 298 K): δ = 8.44 (d, $^3J_{H,H}$ = 8.2 Hz, 1H), 8.34 (d, $^3J_{H,H}$ = 8.3 Hz, 1H), 7.84 (d, $^3J_{H,H}$ = 6.2 Hz, 1H, broad), 7.79 (d, $^3J_{H,H}$ = 6.8 Hz, 1H, broad), 7.72 (s, 1H, broad), 7.00 (s, 1H, broad), 6.41 (s, 1H, broad), 5.16 (s, 1H, broad), 4.32 (s, 1H, broad), 4.09 – 3.91 (m, 1H, broad), 3.49 (ddd, $^2J_{H,H}$ = 29.5, $^2J_{H,P}$ = 14.2, $^4J_{H,H}$ = 2.2 Hz, 2H), 2.90 – 2.72 (m, 1H, broad), 2.07 (s, 3H, broad), 1.56 (d, $^3J_{H,P}$ = 13.9 Hz, 9H), 1.34 (s, 3H, broad), 1.28 (s, 12H, broad, overlapped ^{i}Pr and ^{t}Bu groups), 1.23 (d, $^3J_{H,P}$ = 11.2 Hz, 9H), 1.19 (d, $^3J_{H,P}$ = 11.1 Hz, 9H).

*The spectroscopic features are analogous to complex **1**. However, due to the fluxional process in solution, the signals mainly associated with the p-cymene are broadened. The spectra obtained at 45 °C shows the significant sharpening of the signals.*

^{13}C{^{1}H} NMR (100 MHz, CD_2Cl_2, 298 K): δ = 154.6 (s), 141.1 (s), 138.6 (s), 128.8 (s), 126.1 (s), 125.0 (d, $^3J_{C,P}$ = 8.4 Hz), 122.3 (d, $^3J_{C,P}$ = 8.5 Hz), 121.1 (s), 32.7 (d, $^1J_{C,P}$ = 29.1 Hz), 32.4 (d, $^1J_{C,P}$ = 23.1 Hz), 32.1 (d, $^1J_{C,P}$ = 22.8 Hz), 30.9 (d, $^2J_{C,P}$ = 2.6 Hz), 30.5 (d, $^2J_{C,P}$ = 3.1 Hz), 29.6 (d, $^2J_{C,P}$ = 5.0 Hz), 29.5 (d, $^2J_{C,P}$ = 5.0 Hz).

Due to the fluxional behavior of the complex in solution, several ^{13}C resonances are broadened and of low intensity in the direct ^{13}C{^{1}H} NMR spectrum. Consequently, several carbon signals were identified only through ^{1}H–^{13}C correlation experiments (see HMQC and HMBC spectra). Despite these efforts, some quaternary ^{13}C resonances could not be unambiguously observed or assigned.

^{31}P{^{1}H} NMR (162 MHz, CD_2Cl_2, 298 K): δ = 85.2 (s, 0.7P, broad), 83.7 (s, 0.3P, broad), 37.7 (s, 0.7P, broad), 37.0 (s, 0.3P, broad).

Due to the fluxional process in solution, four ^{31}P resonances are observed at RT. The spectra obtained at 45 °C shows the coalescence of the signals into two singlets.

Anal. Calcd. For $C_{36}H_{58}Cl_2N_2P_2Ru$: C, 57.44; H, 7.77; N, 3.72. **Found:** C, 55.29; H, 8.09; N, 3.63. The observed deviations in the found values are consistent with the presence of residual CH_2Cl_2 in the sample (also detected in the ^{1}H NMR spectrum, ~0.1 equiv; Figure S15) and uptake of water during sample handling. For comparison, inclusion of 1 equiv of H_2O and 0.1 equiv of CH_2Cl_2 gives calculated values in closer agreement with the experimental data*:* **Anal. Calcd. For $C_{36}H_{58}Cl_2N_2P_2Ru$ + 1 H_2O + 0.1 CH_2Cl_2:** C, 55.64; H, 7.79; N, 3.59.

Regardless, the purity of **2** was confirmed by quantitative ^{1}H NMR analysis using 1,3,5-trimethoxybenzene as an internal standard, showing high purity with only trace amounts of residual solvent and minor starting-material impurities.

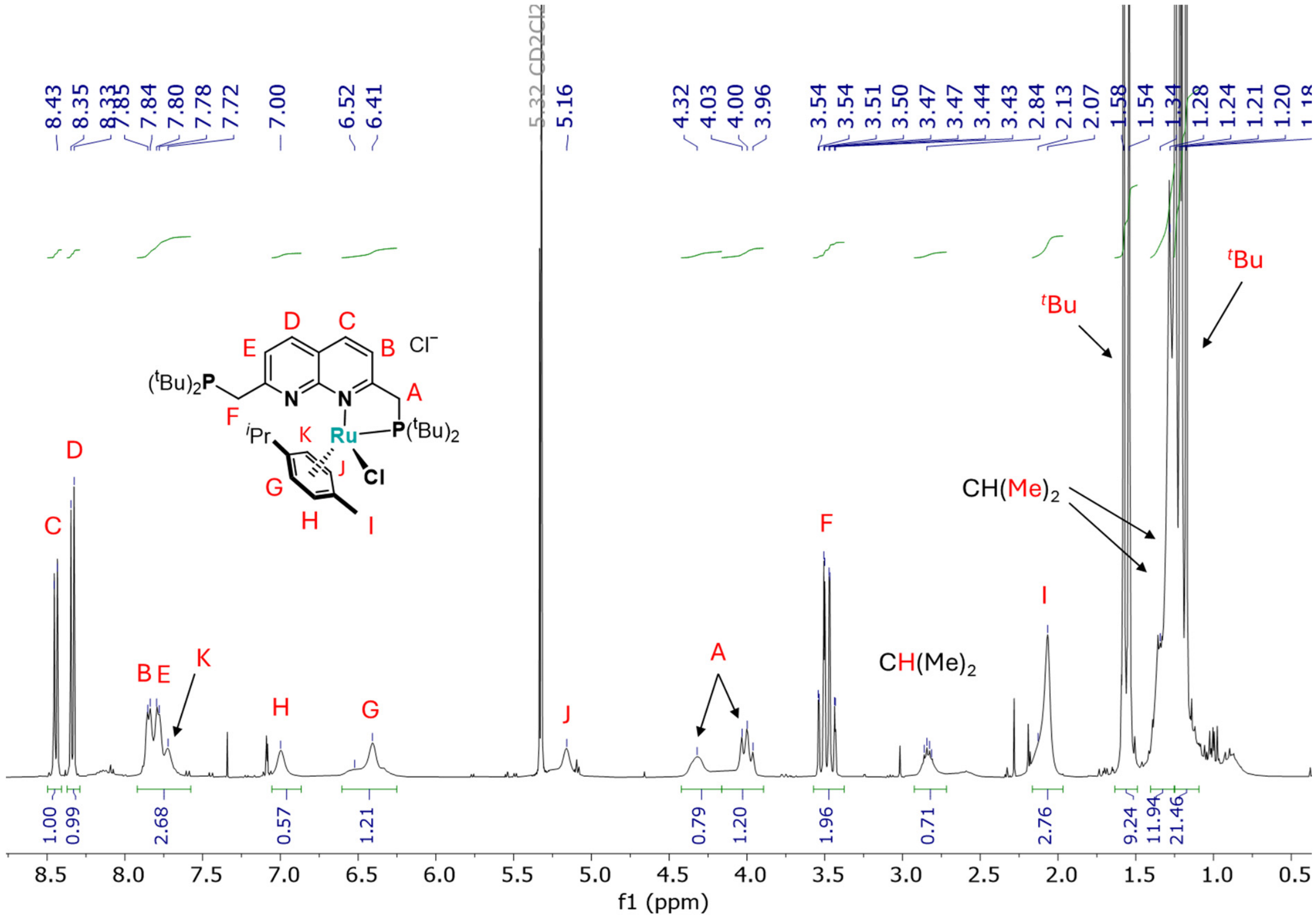

Figure S15. ^{1}H NMR spectrum of **2** in CD_2Cl_2 at 298 K.

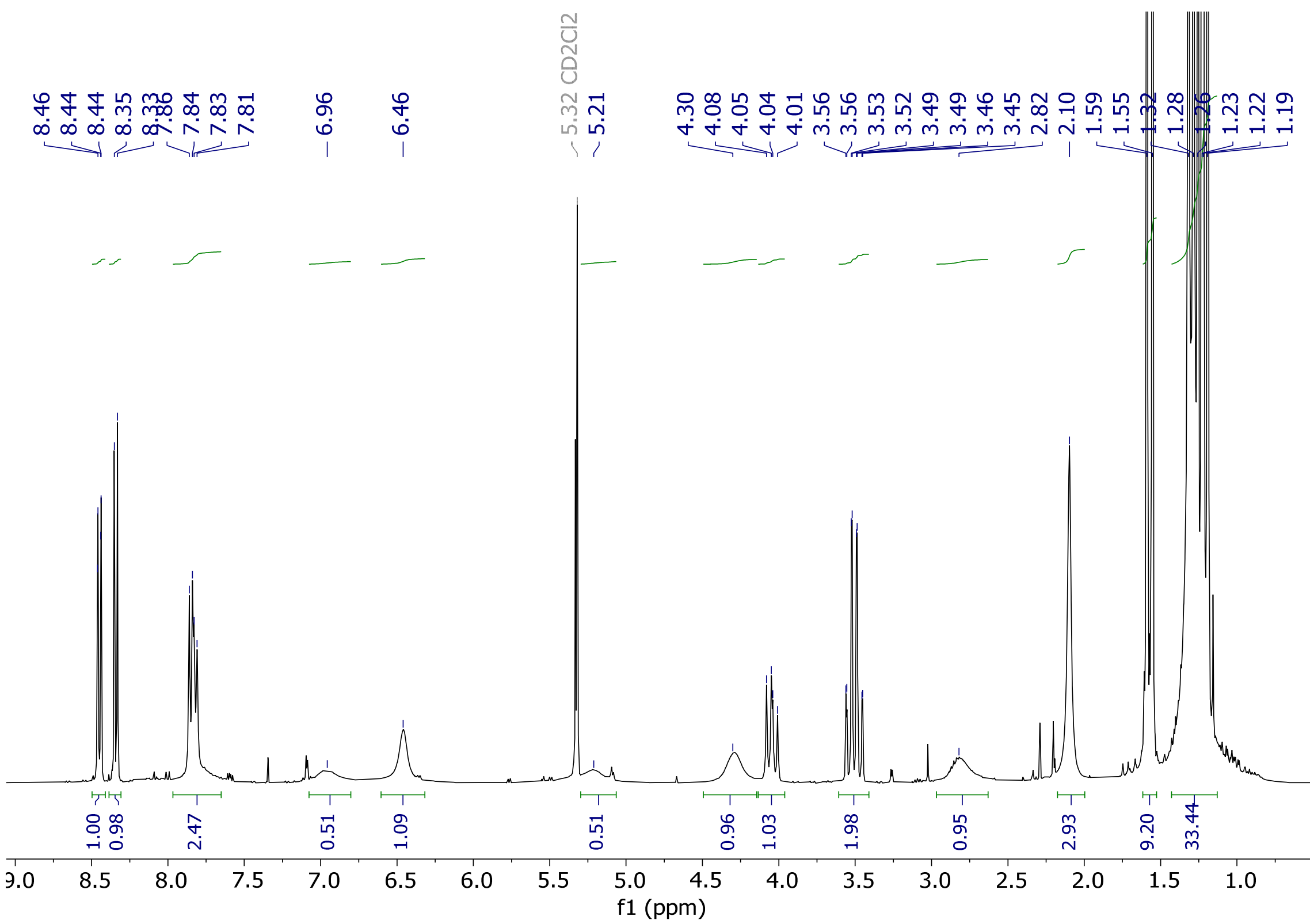

Figure S16. ^{1}H NMR spectrum of **2** in CD_2Cl_2 at 318 K.

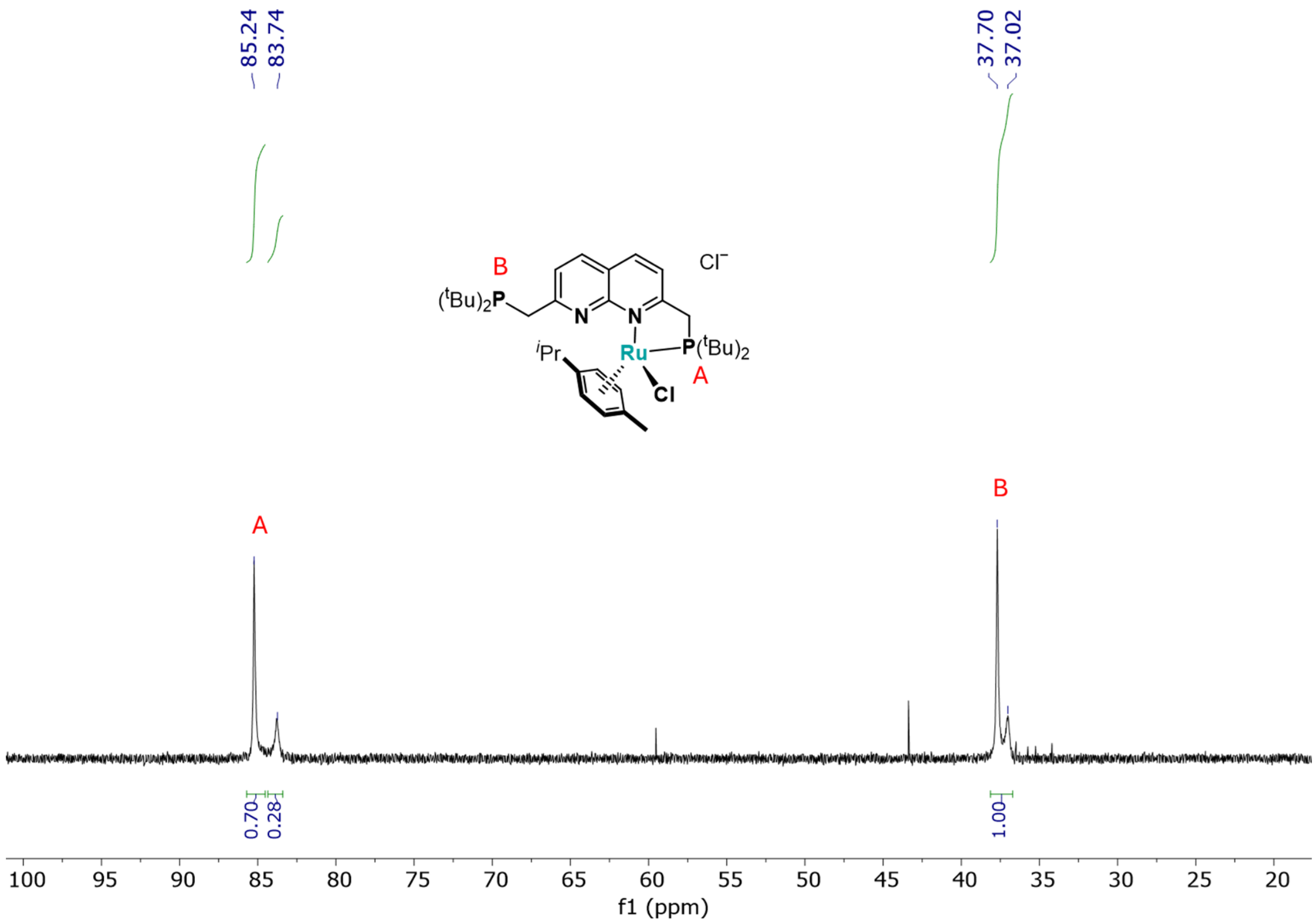

Figure S17. $^{31}P\{^1H\}$ NMR spectrum of **2** in CD_2Cl_2 at 298 K.

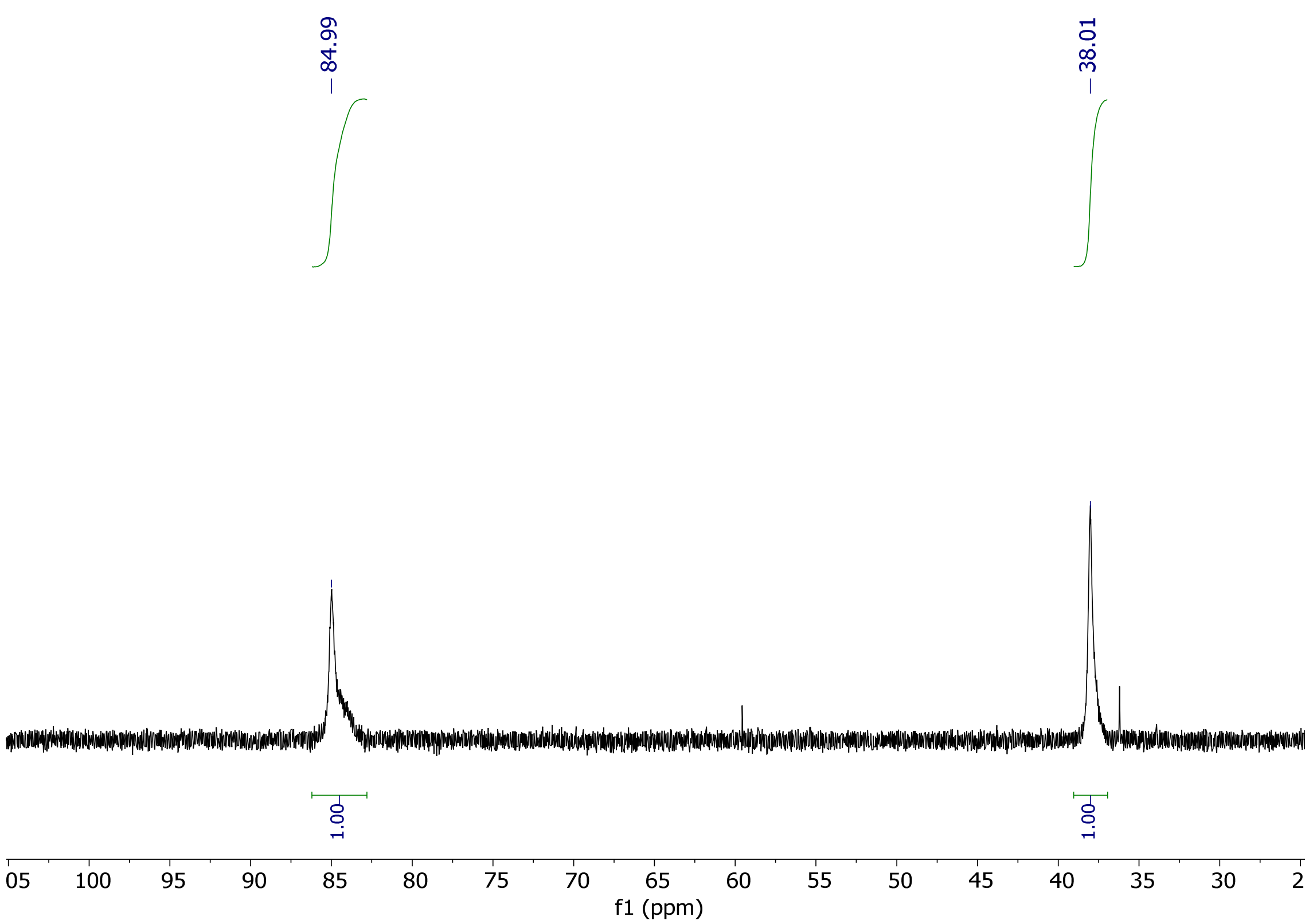

Figure S18. $^{31}P\{^1H\}$ NMR spectrum of **2** in CD_2Cl_2 at 318 K.

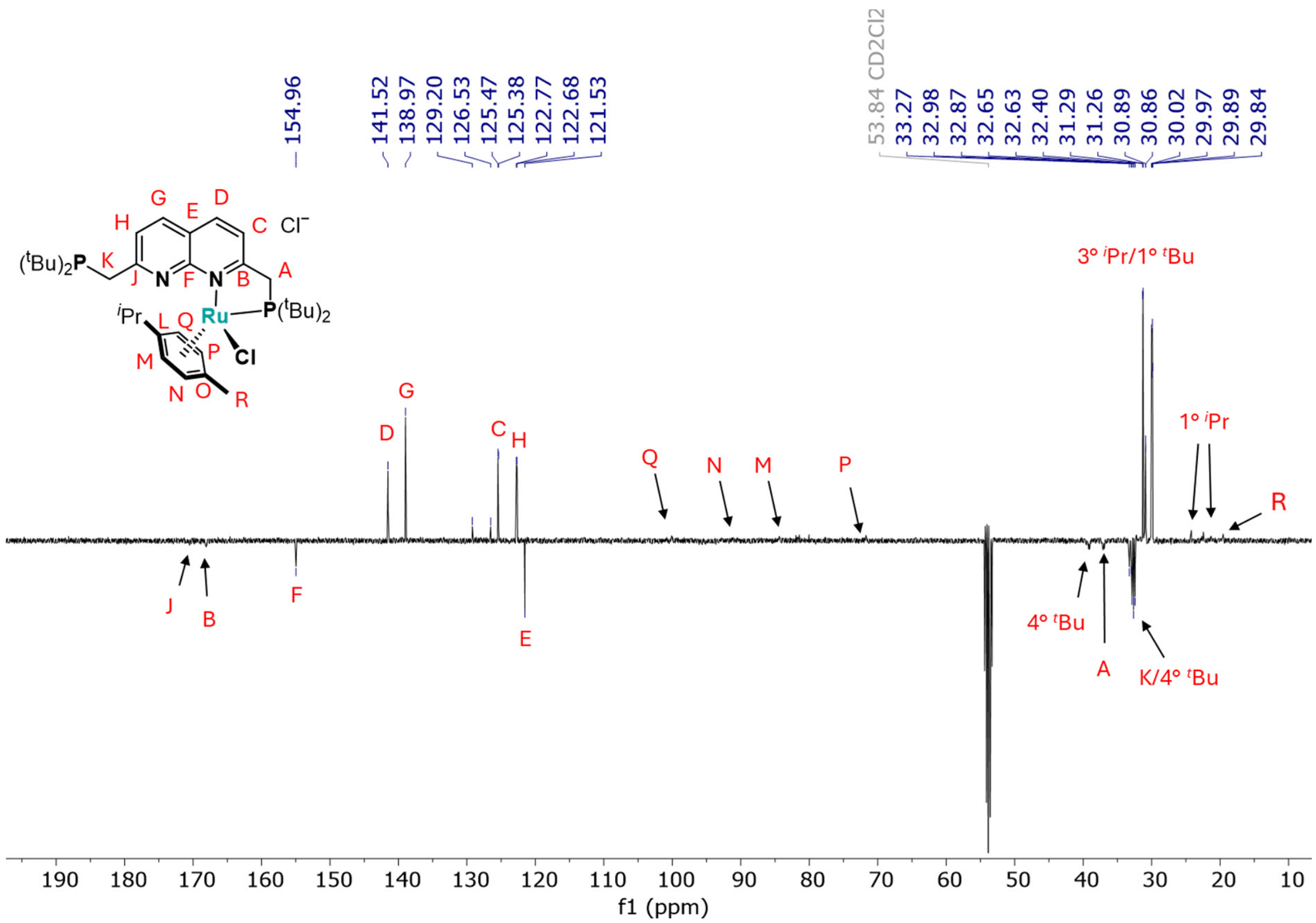

Figure S19. $^{13}C\{^1H\}$ APT NMR spectrum of **2** in CD_2Cl_2 at 298 . Due to fluxionality carbons K. J, B, Q, N, M, P, R, 1° ^{i}Pr were identified based on the 2D NMR and hence their positions are only approximate.

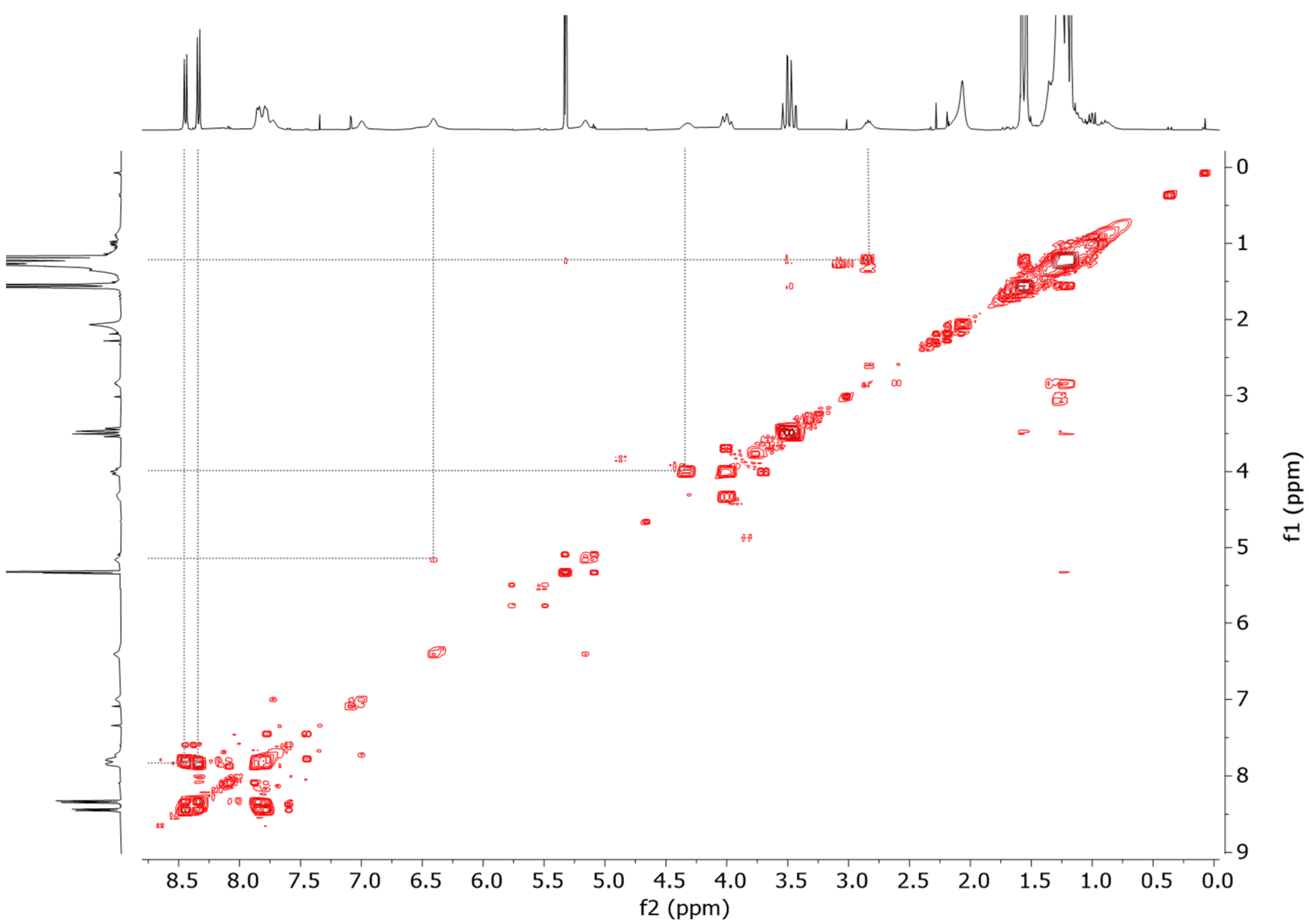

Figure S20. ^1H-^1H COSY NMR spectrum of **2** in CD_2Cl_2 at 298 K.

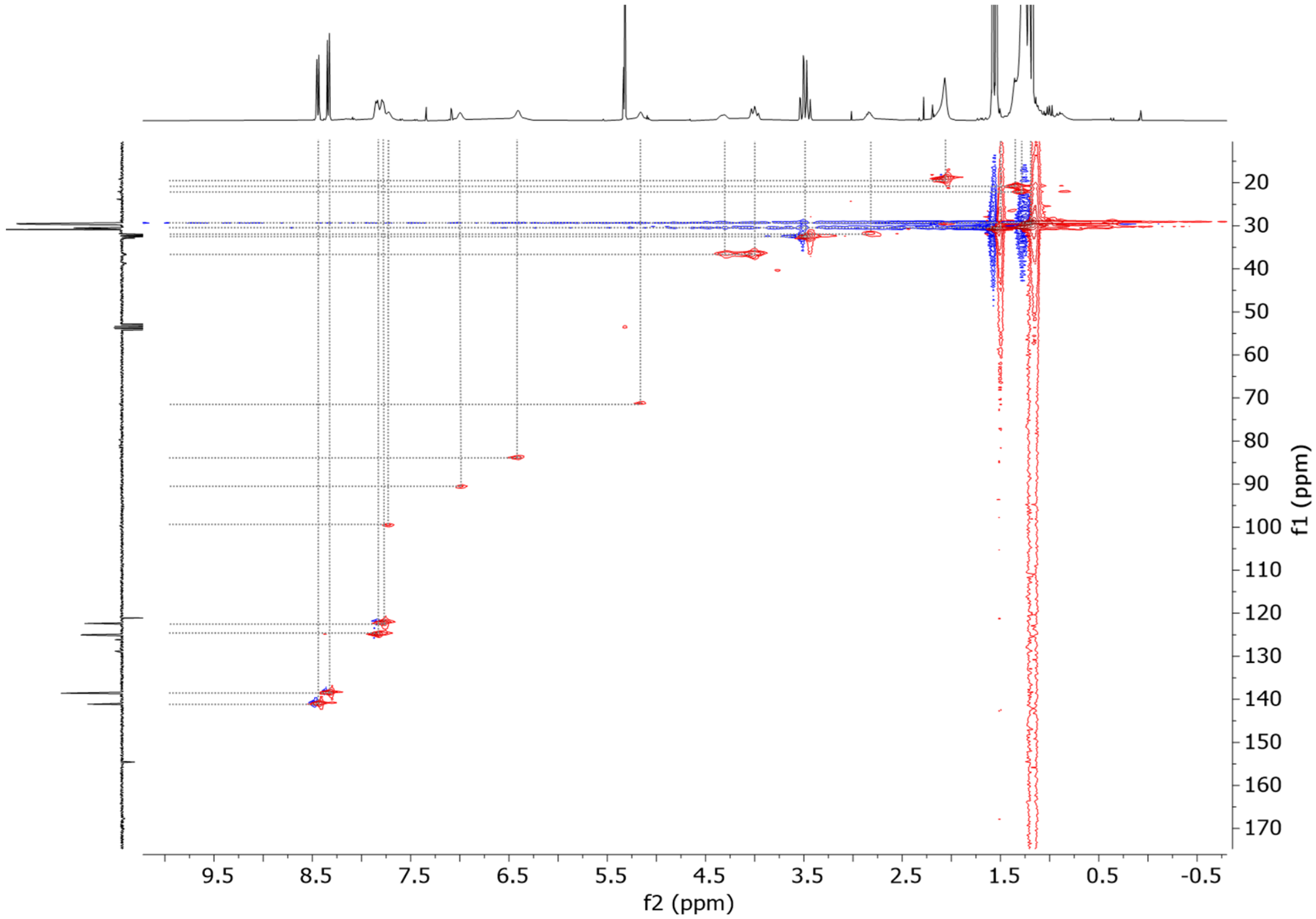

Figure S21. ^{1}H-^{13}C ASAP-HMQC NMR spectrum of **2** in CD_2Cl_2 at 298 K.

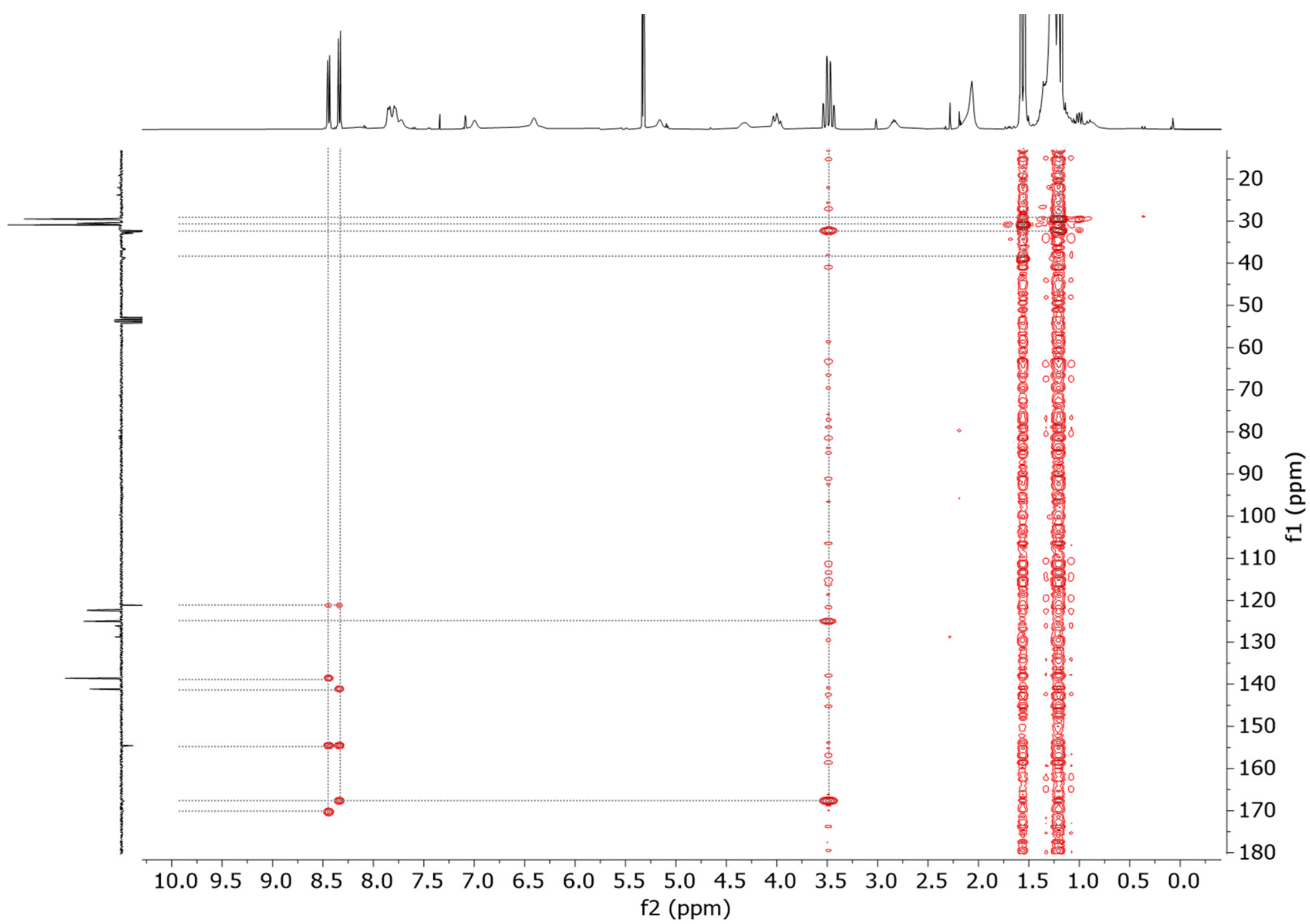

Figure S22. ^{1}H-^{13}C HMBC NMR spectrum of **2** in CD_2Cl_2 at 298 K.

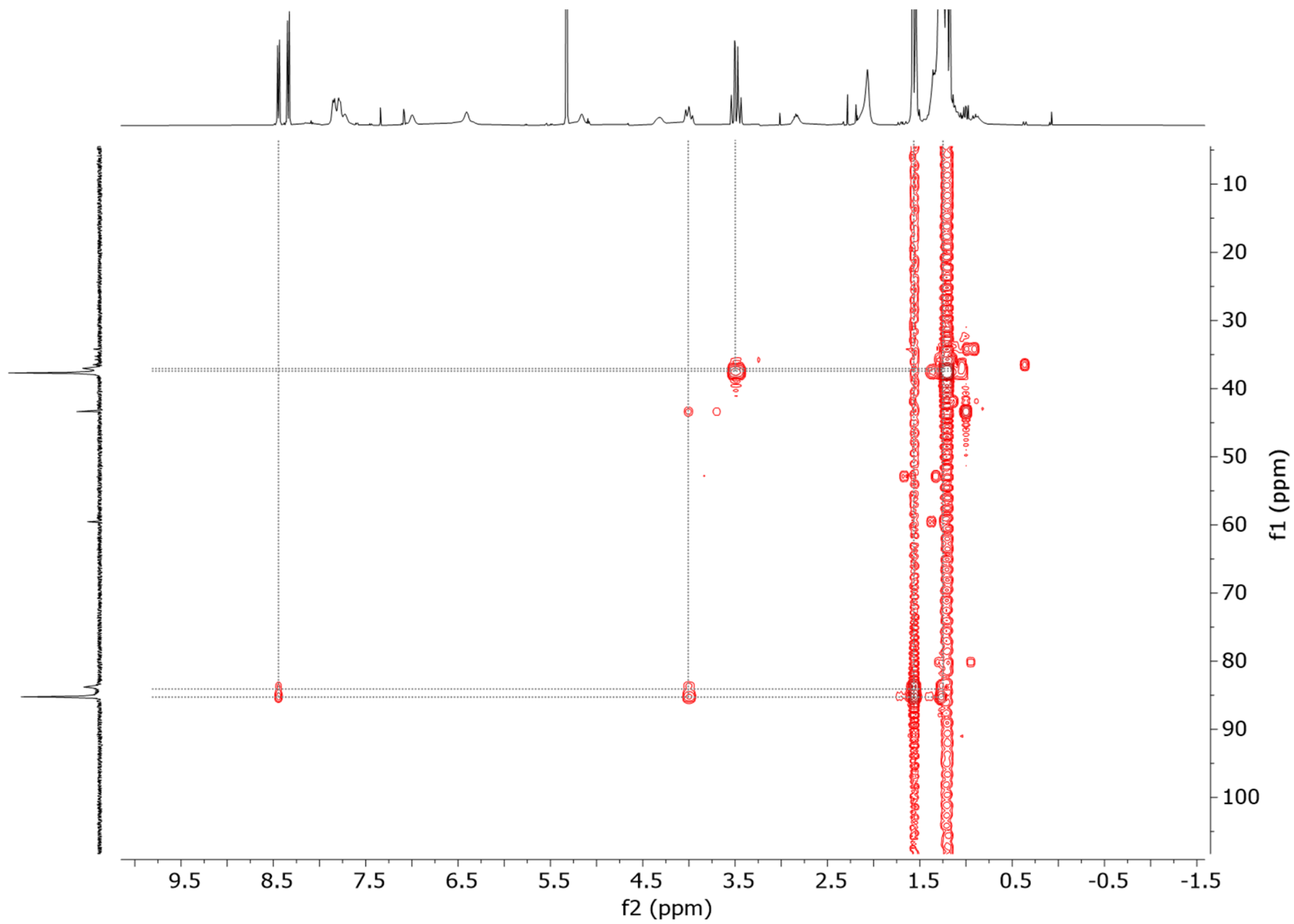

Figure S23. ^{1}H-^{31}P HMBC NMR spectrum of **2** in CD_2Cl_2 at 298 K.

S2.4. [PNNP^{MeCN}-Ru(benzene)] 2Cl (3)

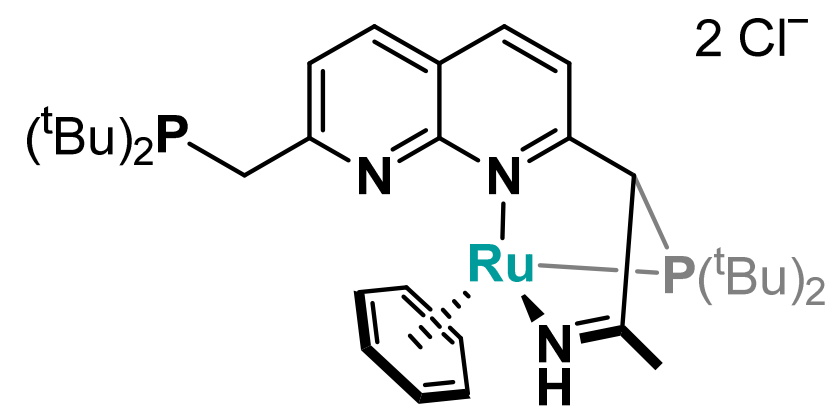

A 20 mL glass vial was charged with **1** (69.67 mg, 0.10 mmol), followed by addition of MeCN (10.0 mL) to give an orange solution. Next, a catalytic amount of Et_3N was added (10%, 1.5 µL). The reaction mixture was kept stirring in a glovebox at RT for 2 h, and the color of the reaction mixture became red with a substantial amount of yellow precipitate formed.* The mixture was filtered through a glass frit to give a yellow residue. It was washed with THF (5.0 mL) and dried under dynamic vacuum to give the product as a yellow powder (67.9 mg, 92%).

* When performing the reaction at a smaller scale, precipitation does not always occur. In that case, removal of all volatiles and identical washing the residue with THF also affords a pure product.

^{1}H NMR (400 MHz, CD_3CN, 298 K): δ = 15.34 (s, 1H, broad), 8.58 (d, $^3J_{H,H}$ = 8.1 Hz, 1H), 8.39 (d, $^3J_{H,H}$ = 8.3 Hz, 1H), 8.10 (d, $^3J_{H,H}$ = 8.1 Hz, 1H), 7.93 (d, $^3J_{H,H}$ = 8.3 Hz, 1H), 6.85 (s, 6H), 5.95 (dd, $^1J_{H,P}$ = 8.6, $^4J_{H,H}$ = 2.1 Hz, 1H), 3.56 (dd, $^1J_{H,P}$ = 3.8, $^4J_{H,H}$ = 2.5 Hz, 34H), 2.55 (d, $^4J_{H,H}$ = 1.2 Hz, 3H), 1.48 (d, $^3J_{H,P}$ = 13.7 Hz, 9H), 1.19 (d, $^3J_{H,P}$ = 11.0 Hz, 9H), 1.15 (d, $^3J_{H,P}$ = 11.0 Hz, 9H), 0.94 (d, $^3J_{H,P}$ = 13.9 Hz, 9H).

^{13}C{^{1}H} NMR (100 MHz, CD_3CN, 298 K): δ = 185.5 (d, $^2J_{C,P}$ = 5.6 Hz), 170.9 (d, $^2J_{C,P}$ = 10.6 Hz), 163.4 (s), 156.4 (s), 144.0 (s), 140.1 (s), 127.6 (d, $^3J_{C,P}$ = 6.4 Hz), 123.2 (d, $^3J_{C,P}$ = 4.9 Hz), 121.9 (s), 92.5 – 91.6 (m), 63.0 (d, $^1J_{C,P}$ = 16.0 Hz), 41.1 (d, $^1J_{C,P}$ = 9.7 Hz), 40.0 (d, $^1J_{C,P}$ = 7.6 Hz), 33.9 (d, $^1J_{C,P}$ = 27.8 Hz), 31.7 (d, $^2J_{C,P}$ = 2.1 Hz), 31.0 (d, $^2J_{C,P}$ = 2.7 Hz), 30.2 (d, $^2J_{C,P}$ = 5.4 Hz), 30.0 (d, $^2J_{C,P}$ = 5.3 Hz), 24.3 (d, $^3J_{C,P}$ = 2.7 Hz).

^{31}P{^{1}H} NMR (162 MHz, CD_3CN, 298 K): δ = 172.3 (s, 1P), 40.1 (s, 1P).

The purity of **3** was confirmed by quantitative ^{1}H NMR analysis using 1,3,5-trimethoxybenzene as an internal standard, showing high purity with only trace amounts of residual THF and free PNNP ligand.

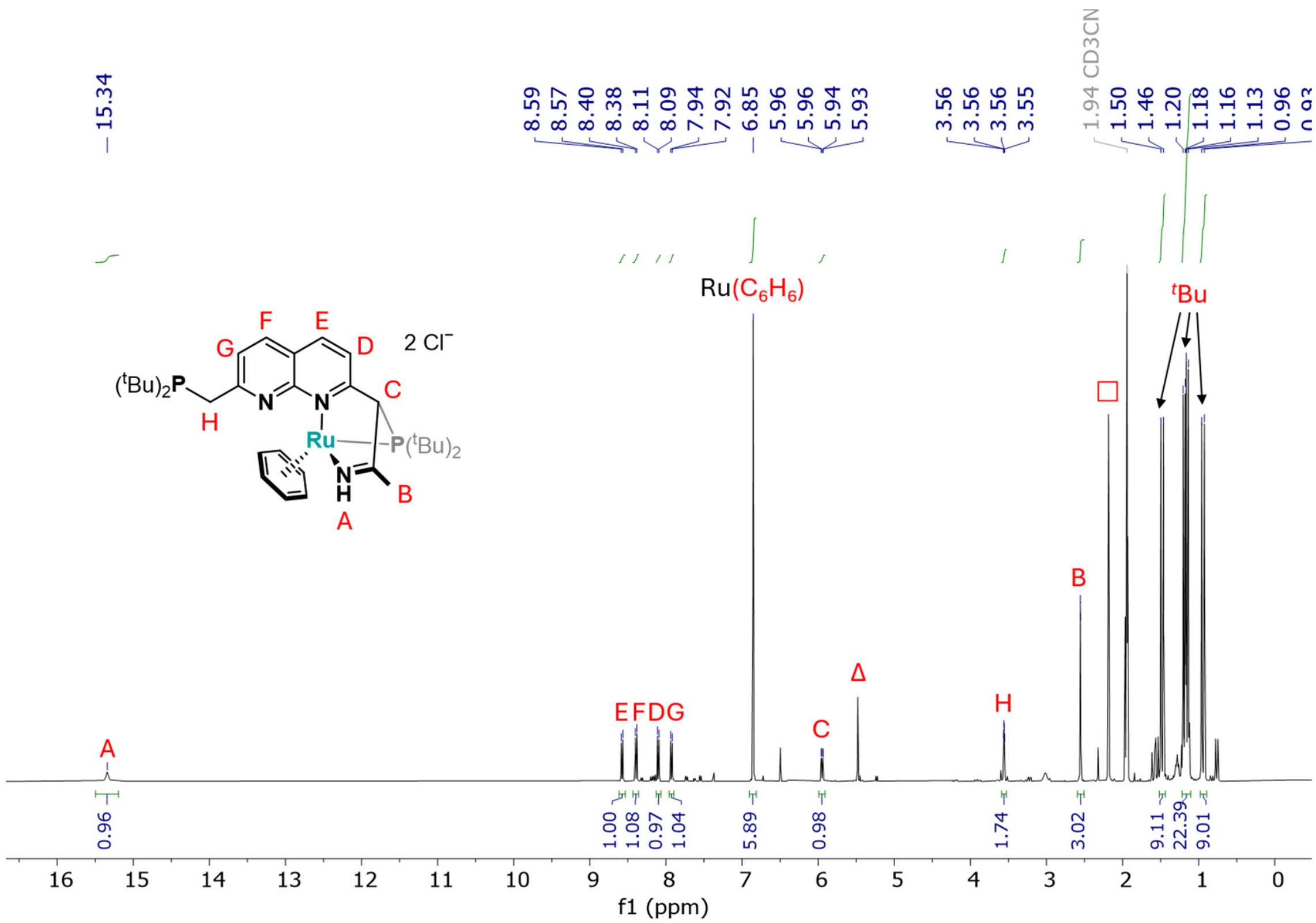

Figure S24. ^{1}H NMR spectrum of **3** in CD_3CN at 298 K. The triangle and the square presumably denote minor species featuring a Ru(η^6-benzene) core with coordinated MeCN ligands.

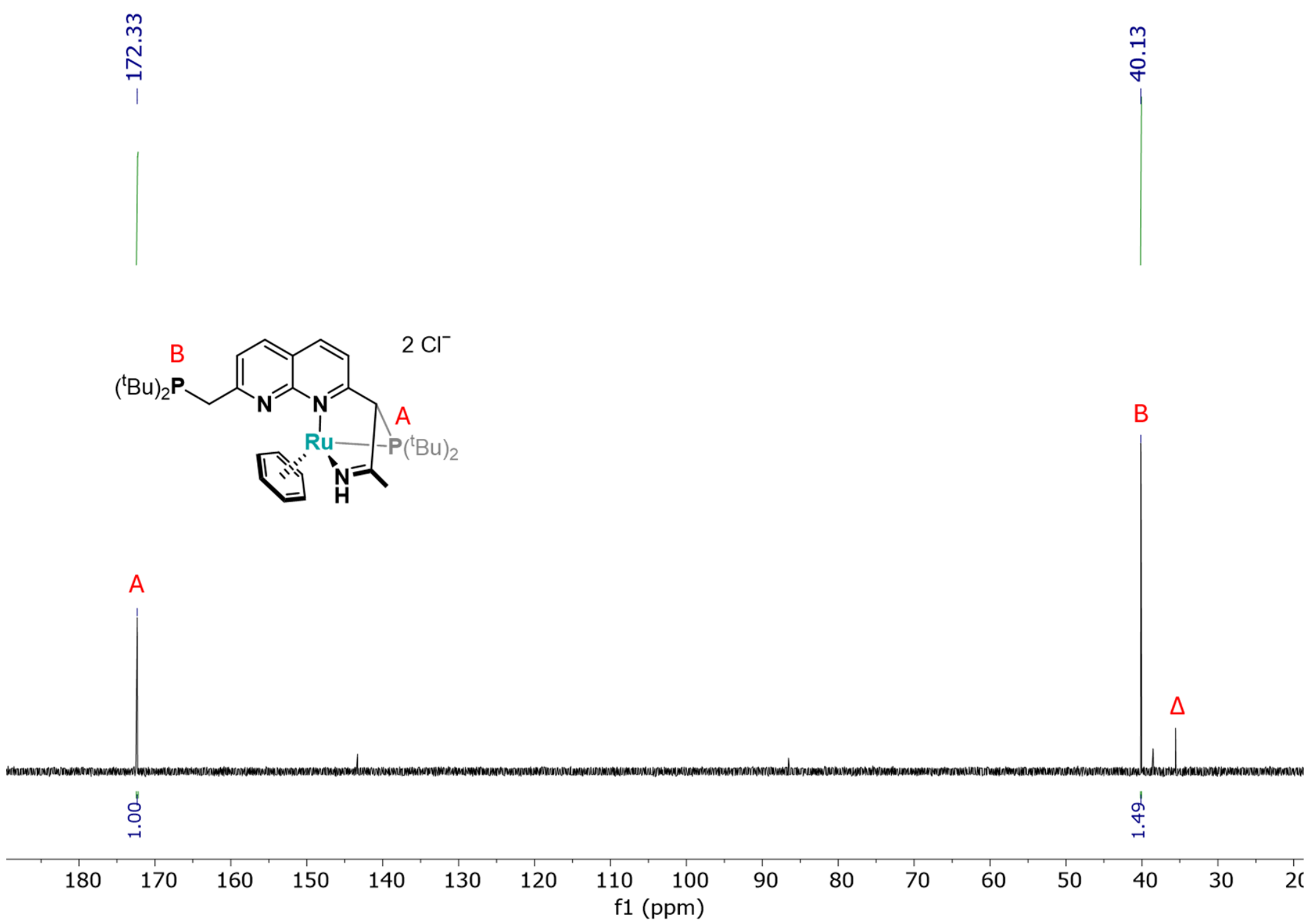

Figure S25. $^{31}P\{^{1}H\}$ NMR spectrum of **3** in CD_3CN at 298 K. The triangle denotes minor impurity of PNNP ligand.

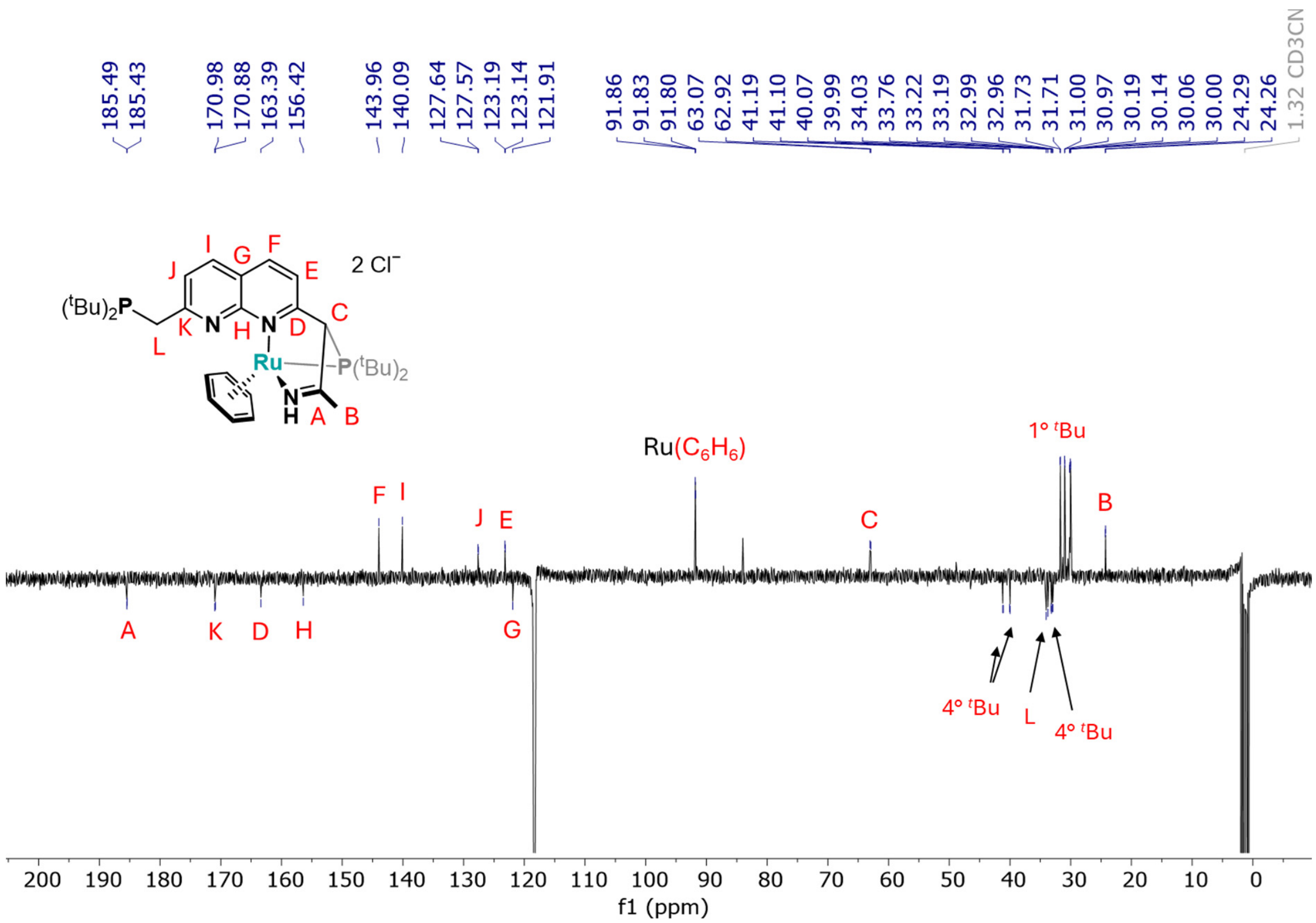

Figure S26. $^{13}C\{^{1}H\}$ APT NMR spectrum of **3** in CD_3CN at 298 K.

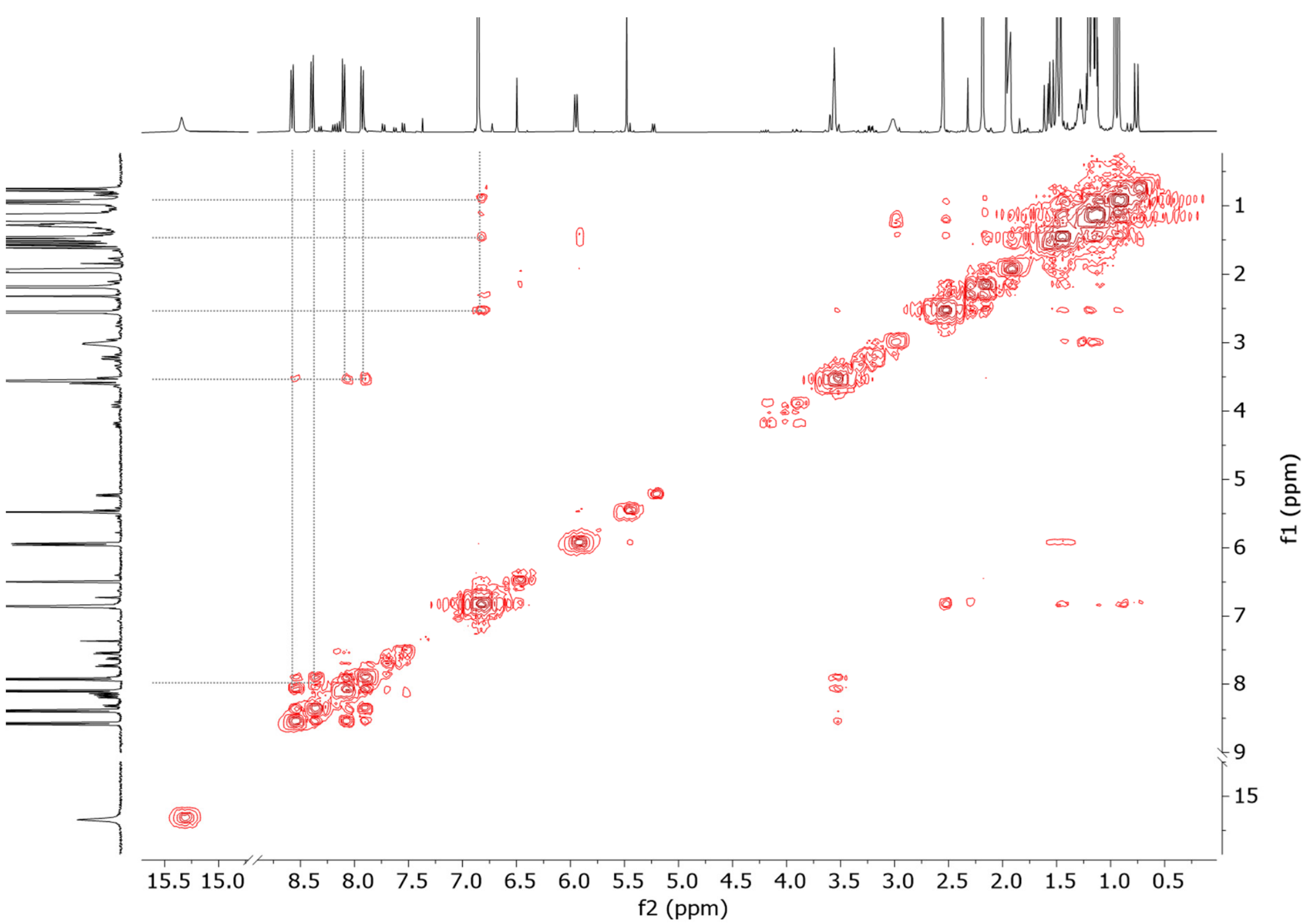

Figure S27. ^{1}H-^{1}H COSY NMR spectrum of **3** in CD_3CN at 298 K.

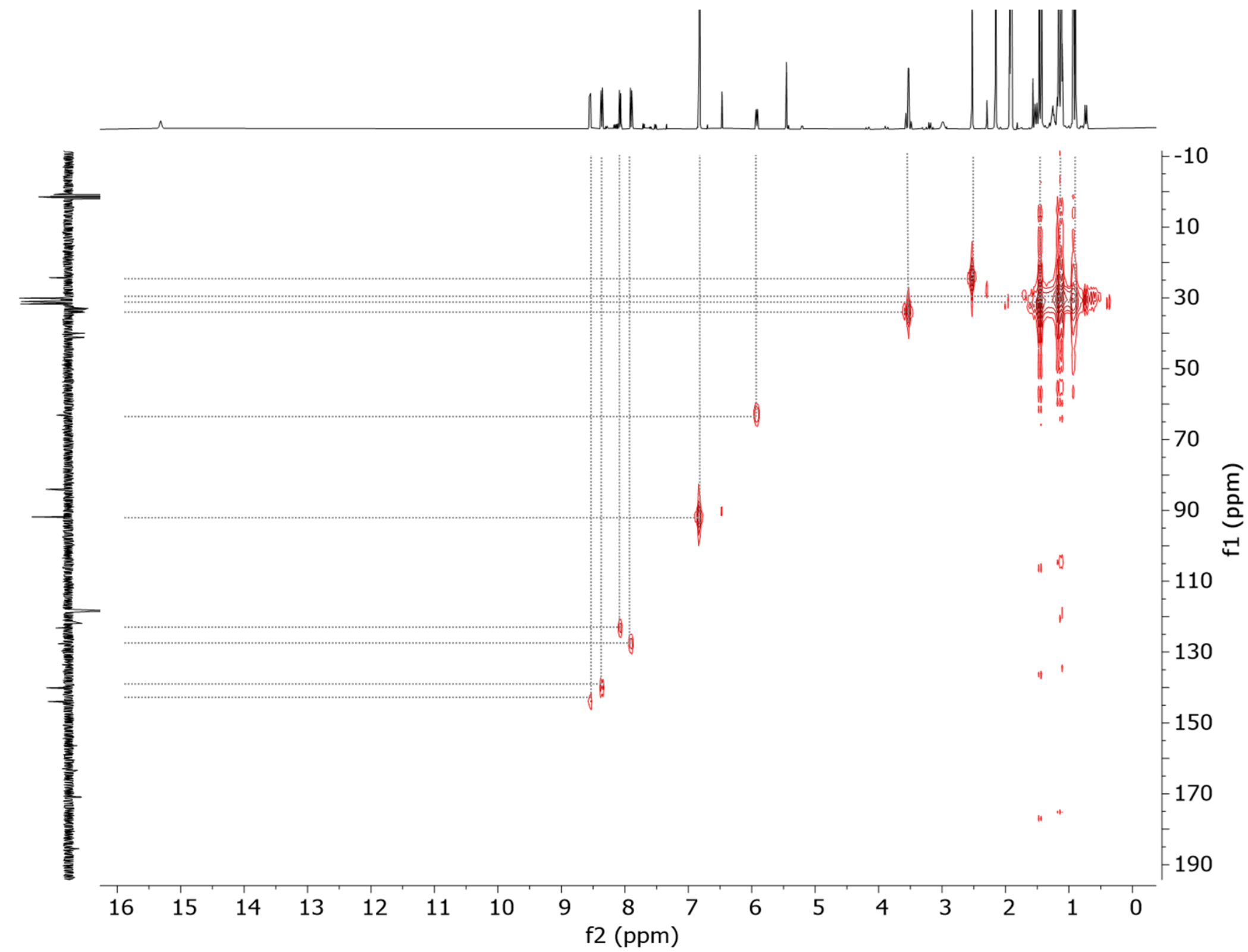

Figure S28. ^{1}H-^{13}C ASAP-HMQC NMR spectrum of **3** in CD_3CN at 298 K.

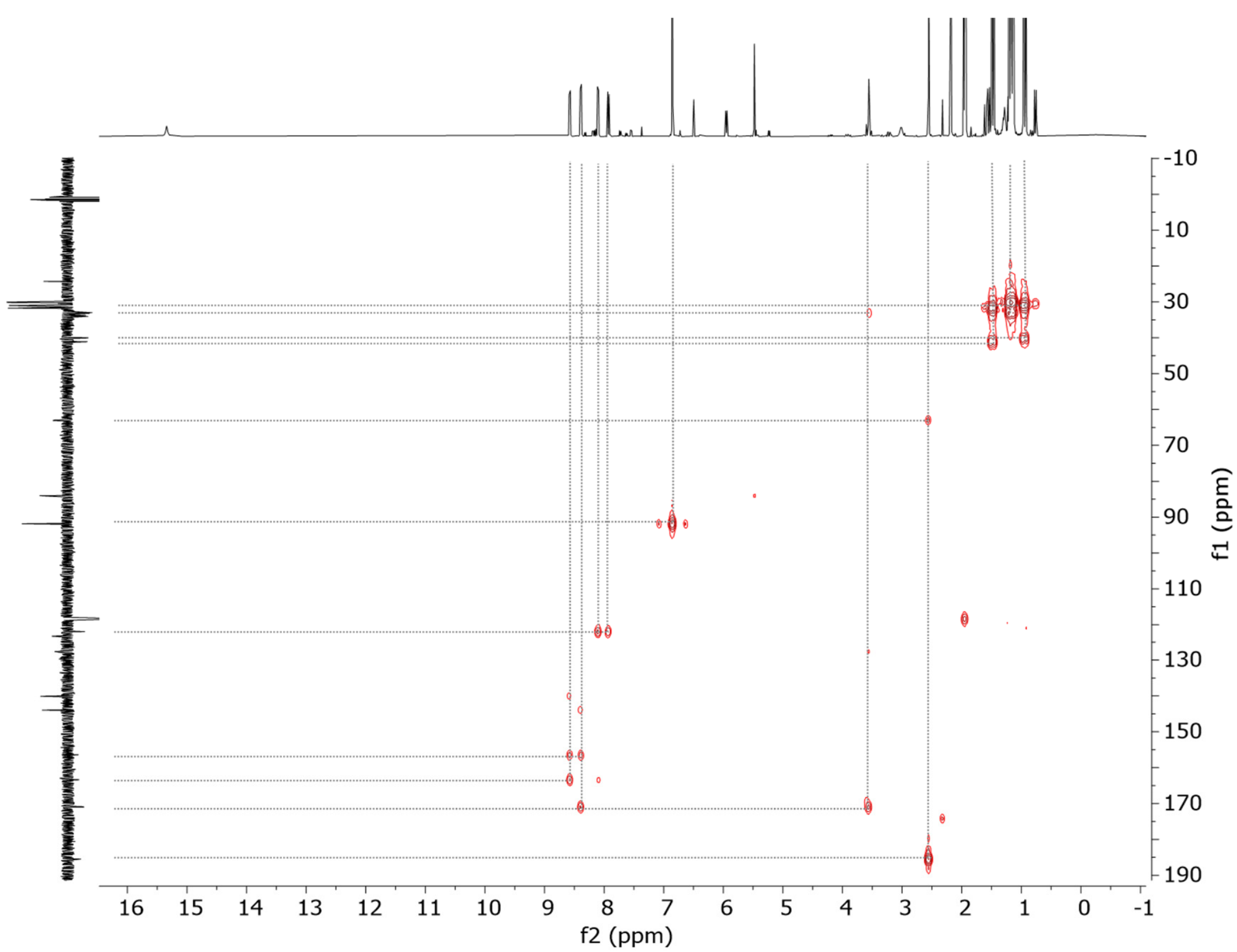

Figure S29. ^{1}H-^{13}C HMBC NMR spectrum of **3** in CD_3CN at 298 K.

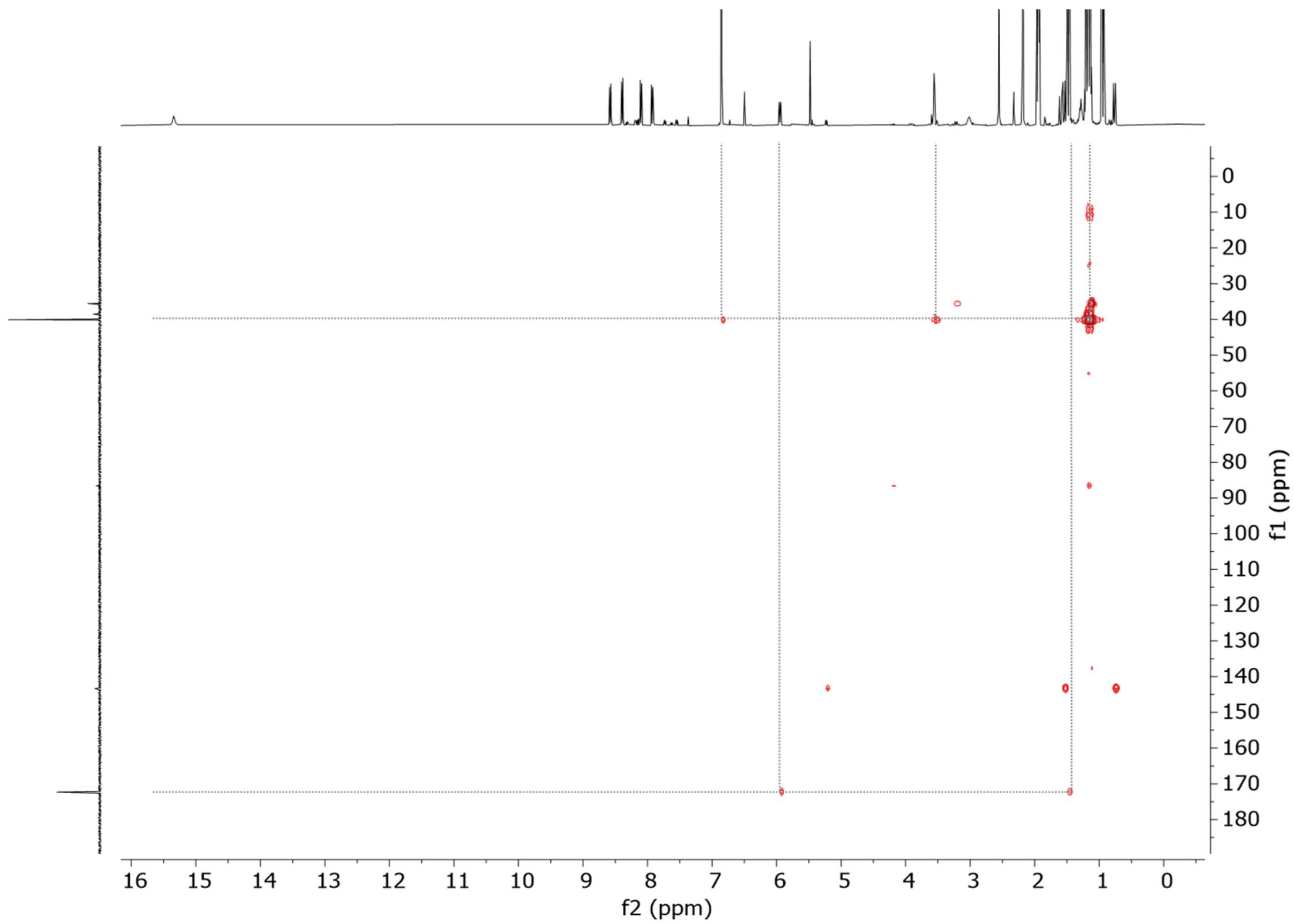

Figure S30. ^{1}H-^{31}P HMBC NMR spectrum of **3** in CD_3CN at 298 K.

S2.5. [PNNP^{MeCN}-Ru(*p*-cymene)] 2Cl (4)

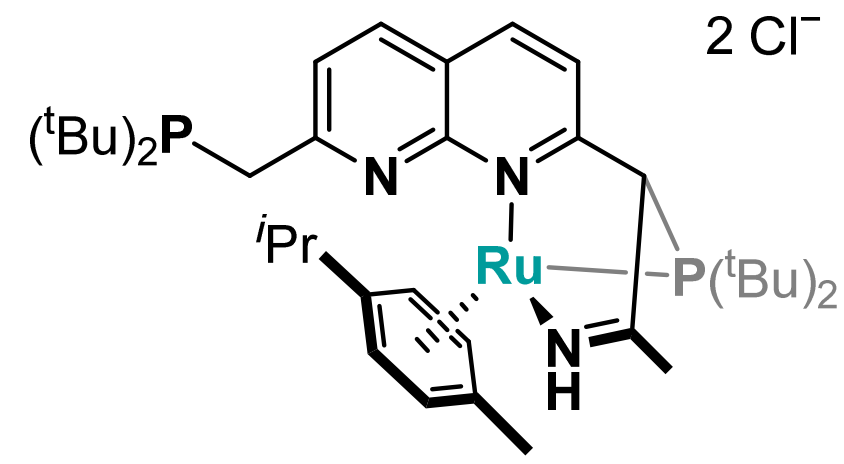

A 20 mL glass vial was charged with **2** (75.3 mg, 0.10 mmol), followed by addition of MeCN (10.0 mL) to give an orange solution. Next, a catalytic amount of Et_3N was added (10%, 1.5 µL). The reaction mixture was kept stirring in a glovebox at RT for 2 h, and the color of the reaction mixture became red with a substantial amount of yellow precipitate formed.* The mixture was filtered through a glass frit to give a yellow residue. It was washed with THF (5.0 mL) and dried under dynamic vacuum to give the product as a yellow powder (64.9 mg, 81%).

* When performing the reaction at a smaller scale, precipitation does not always occur. In that case, removal of all volatiles and identical washing the residue with THF also affords a pure product.

Note: *This compound can also be prepared via the direct reaction of the PNNP ligand with [Ru_2Cl_4(p-cymene)$_2$] in MeCN in the presence of a catalytic amount of Et_3N.*

^{1}H NMR (400 MHz, CD_3CN, 298 K): δ = 14.40 (s, 1H, broad), 8.65 (d, $^3J_{H,H}$ = 8.1 Hz, 1H), 8.45 (d, $^3J_{H,H}$ = 8.3 Hz, 1H), 8.28 (d, $^3J_{H,H}$ = 8.2 Hz, 1H), 8.28 – 8.24 (m, 1H, broad), 7.92 (d, $^3J_{H,H}$ = 9.1 Hz, 1H), 6.86 (d, $^3J_{H,H}$ =6.2 Hz, 1H), 6.68 (d, $^3J_{H,H}$ = 8.2 Hz, 1H), 6.26 (dd, $^1J_{H,P}$ = 8.6, $^4J_{H,H}$ = 2.2 Hz, 1H), 5.78 (d, $^3J_{H,H}$ = 5.2 Hz, 1H, broad), 4.21 (hept, $^3J_{H,H}$ = 6.6 Hz, 1H), 3.60 (d, $^1J_{H,P}$ = 2.6 Hz, 2H), 2.70 (d, $^4J_{H,H}$ = 1.4 Hz, 3H), 2.45 (s, 3H), 1.49 (d, $^3J_{H,P}$ = 13.5 Hz, 9H), 1.33 (d, $^3J_{H,H}$ = 6.9 Hz, 3H), 1.20 (d, $^3J_{H,P}$ = 11.1 Hz, 9H), 1.16 (d, $^3J_{H,P}$ = 11.1 Hz, 9H), 0.92 (d, $^3J_{H,H}$ = 7.1 Hz, 3H), 0.89 (d, $^3J_{H,P}$ = 13.5 Hz, 9H).

^{13}C{^{1}H} NMR (100 MHz, CD_3CN, 298 K): δ = 186.4 (s), 170.7 (d, $^2J_{C,P}$ = 10.4 Hz), 164.0 (s), 156.6 (s), 143.9 (s), 140.1 (s), 127.5 (d, $^3J_{C,P}$ = 5.7 Hz), 123.5 (d, $^3J_{C,P}$ = 4.9 Hz), 122.1 (s), 119.8 (d, $^2J_{C,P}$ = 7.8 Hz), 108.0 (s), 99.5 (d, $^2J_{C,P}$ = 6.5 Hz), 89.9 (d, $^2J_{C,P}$ = 8.4 Hz), 88.8 (s), 80.3 (s), 62.9 (d, $^1J_{C,P}$ = 16.4 Hz), 41.0 (d, $^1J_{C,P}$ = 6.9 Hz), 39.8 (d, $^1J_{C,P}$ = 7.2 Hz), 34.0 (d, $^1J_{C,P}$ = 27.6 Hz), 33.2 (d, $^1J_{C,P}$ = 13.4 Hz), 33.0 (d, $^1J_{C,P}$ = 12.5 Hz), 31.9 (d, $^2J_{C,P}$ = 2.8 Hz), 31.2 (d, $^2J_{C,P}$ = 2.3 Hz), 31.1 (s), 30.1 (d, $^1J_{C,P}$ = 13.3 Hz), 30.1 (d, $^1J_{C,P}$ = 13.3 Hz), 25.6 (s), 24.4 (d, $^3J_{C,P}$ = 2.4 Hz), 22.0 (s), 21.1 (s).

^{31}P{^{1}H} NMR (162 MHz, CD_3CN, 298 K): δ = 169.4 (s, 1P), 39.0 (s, 1P).

Anal. Calcd. For $C_{38}H_{61}Cl_2N_3P_2Ru$: C, 57.49; H, 7.75; N, 5.29. **Found:** C, 54.86; H, 8.11; N, 5.38. *The observed deviations are consistent with the uptake of water during sample handling. For comparison, inclusion of 2 equiv of H_2O gives calculated values that are in closer agreement with the experimental data:* **Anal. Calcd. For $C_{38}H_{61}Cl_2N_3P_2Ru$ + 2 H_2O:** C, 55.00; H, 7.90; N, 5.06.

Regardless, the purity of **4** was confirmed by quantitative ^{1}H NMR analysis using 1,3,5-trimethoxybenzene as an internal standard, indicating high purity with only trace amounts of residual THF.

ATR-IR (film, N_2 flow, 298 K): ν = 2937 (s), 2897 (s), 2864 (s), 2757 (w), 2592 (w), 2255 (w), 2218 (w), 2097 (w), 1610 (m), 1593 (m), 1539 (m), 1504 (m), 1472 (m), 1441 (m), 1396 (m), 1367 (m), 1289 (w), 1255 (w), 1226 (w), 1173 (m), 1057 (w), 1024 (w), 933 (w), 910 (w), 872 (w), 841 (w), 806 (w), 696 (w), 658 (w), 638 (w), 620 (w) cm^{-1}.

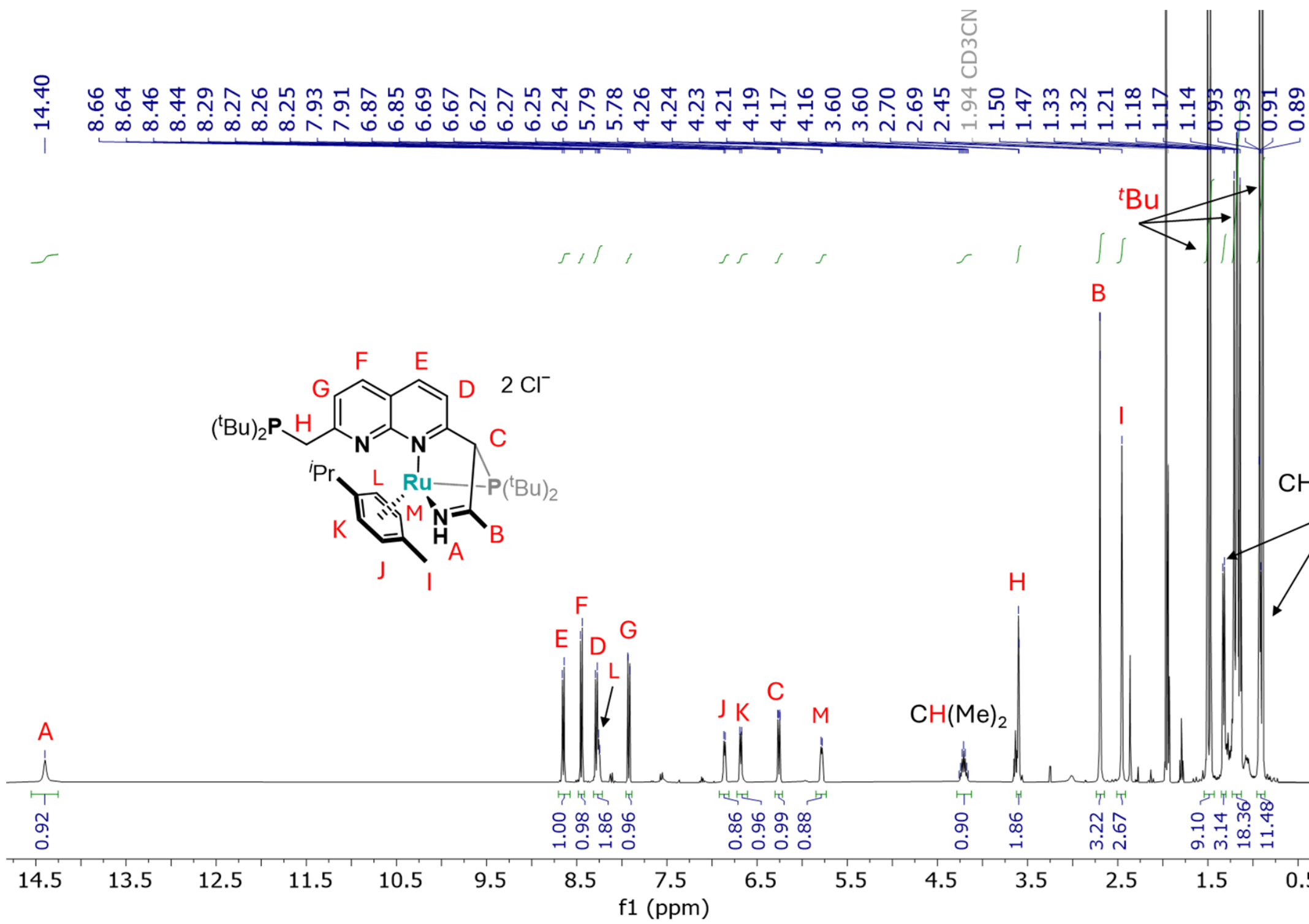

Figure S31. ^{1}H NMR spectrum of **4** in CD_3CN at 298 K.

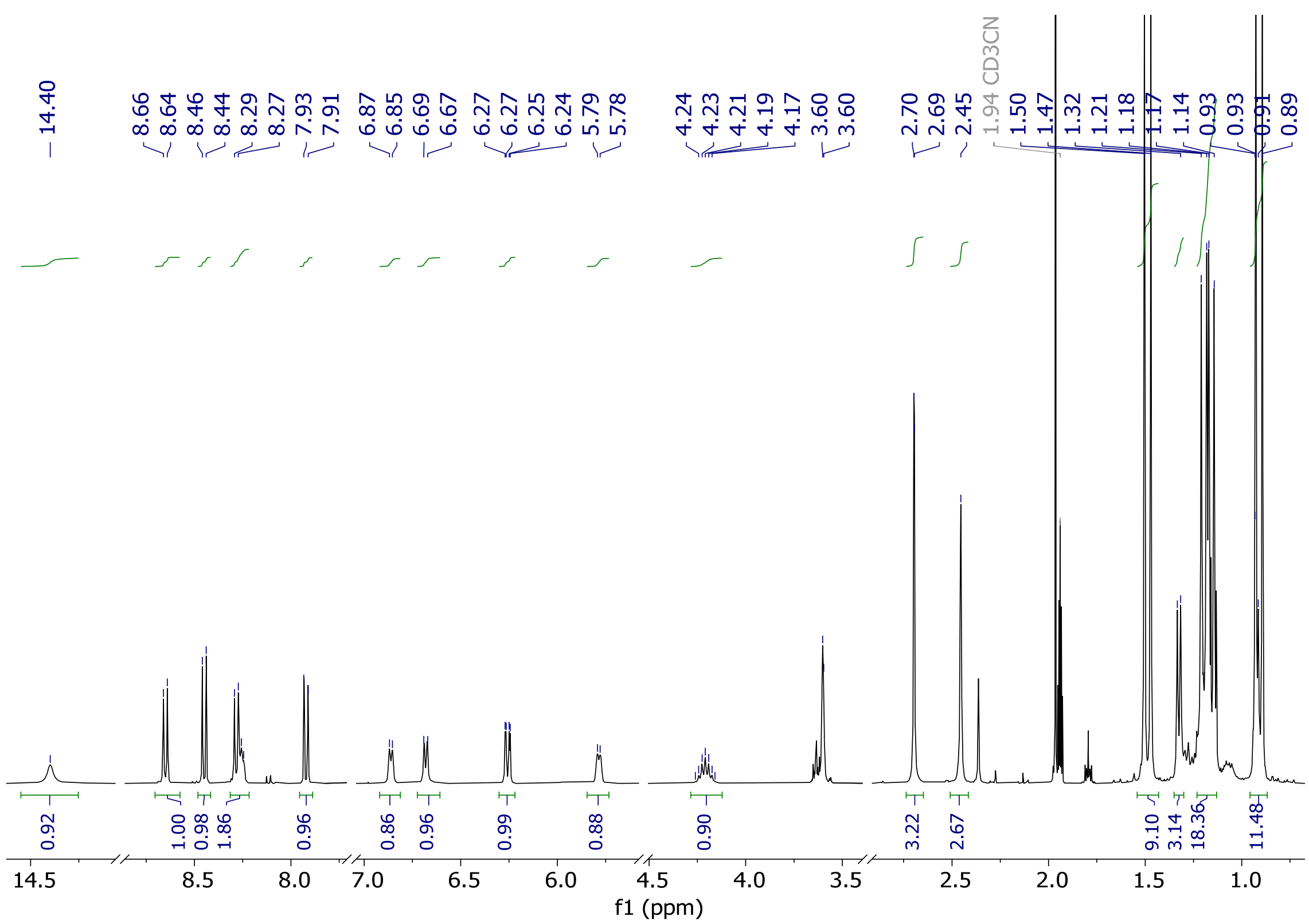

Figure S32. ^{1}H NMR spectrum of **4** in CD_3CN at 298 K (zoomed-in).

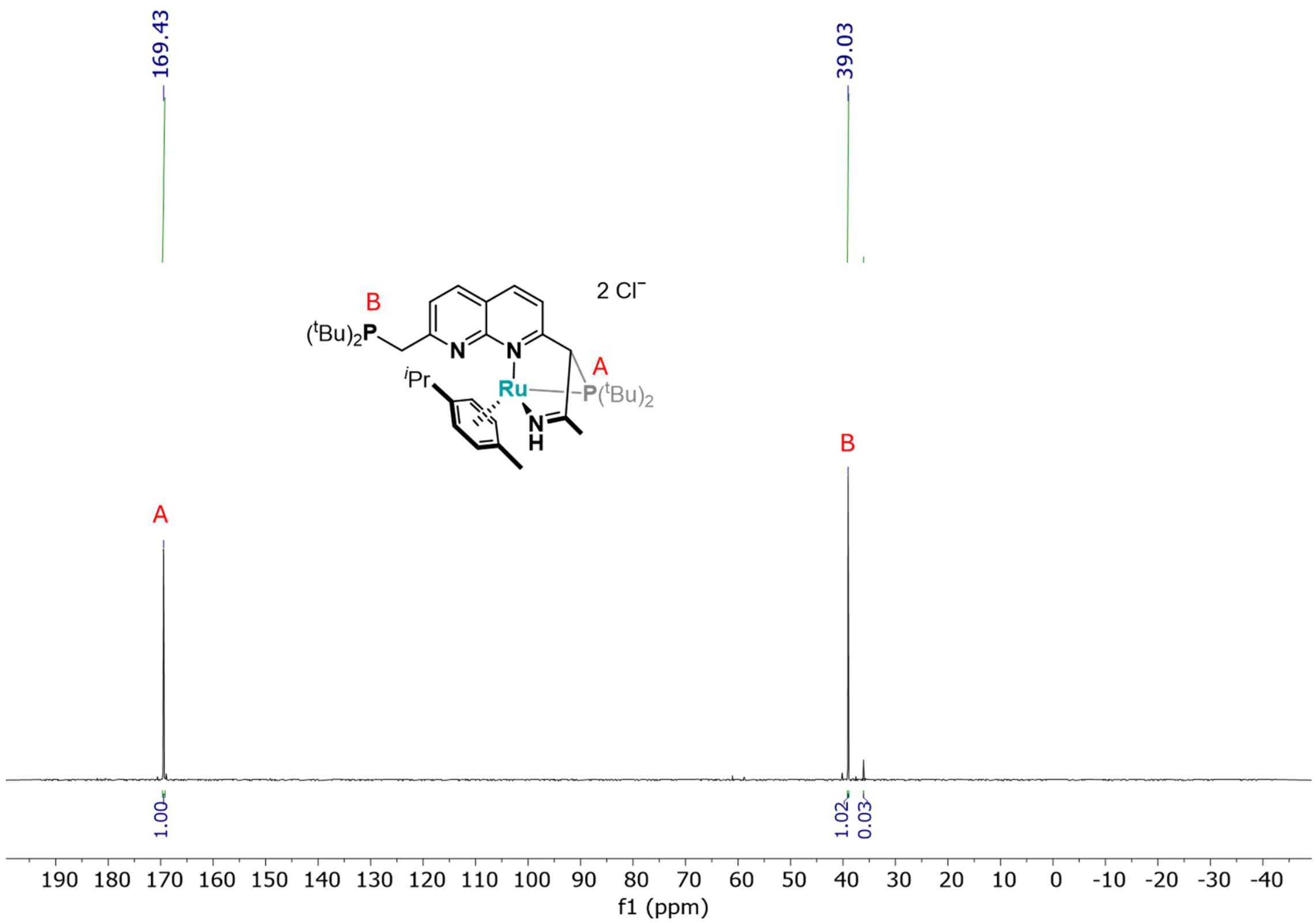

Figure S33. $^{31}P\{^{1}H\}$ NMR spectrum of **4** in CD_3CN at 298 K.

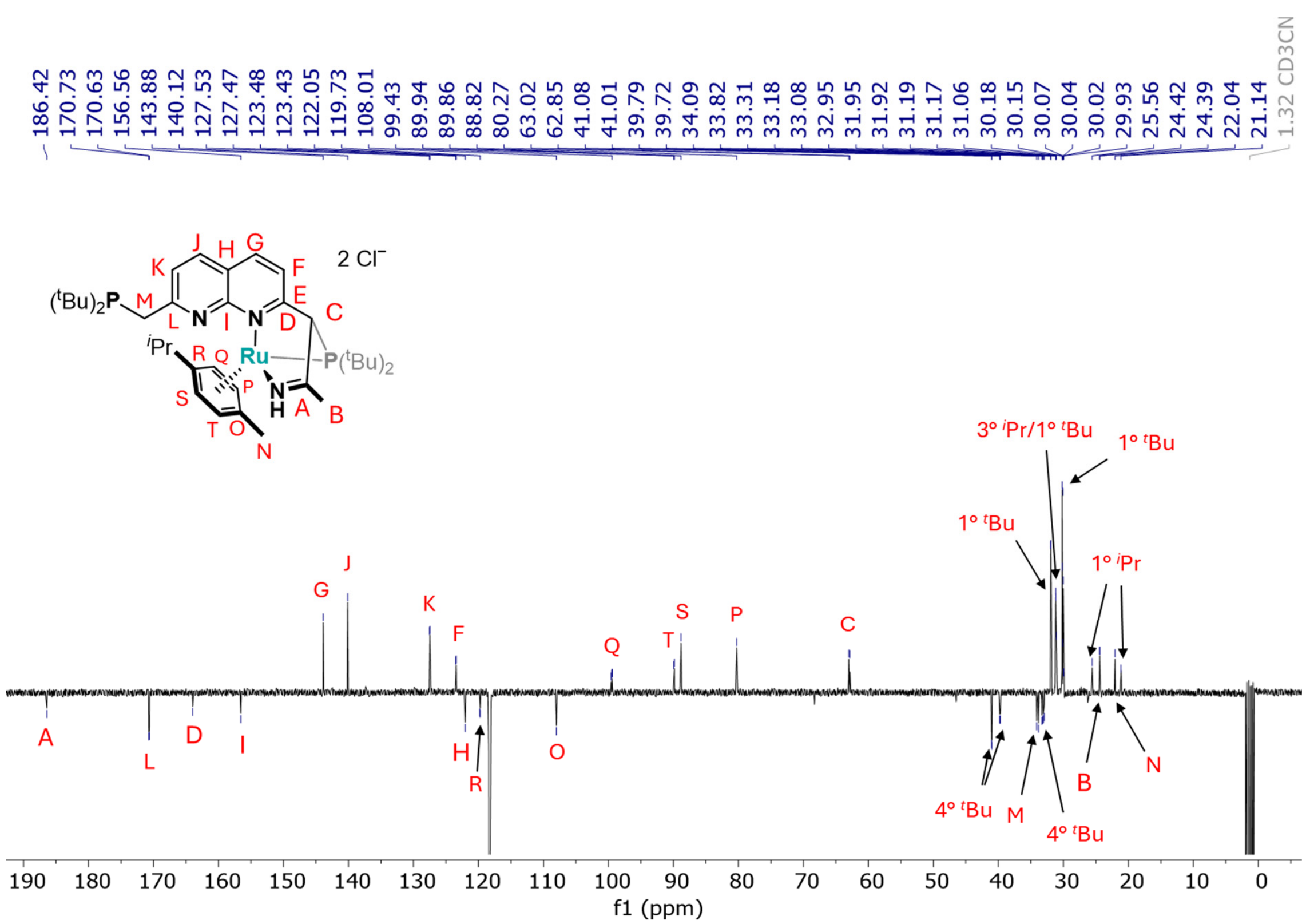

Figure S34. $^{13}C\{^{1}H\}$ APT NMR spectrum of **4** in CD_3CN at 298 K.

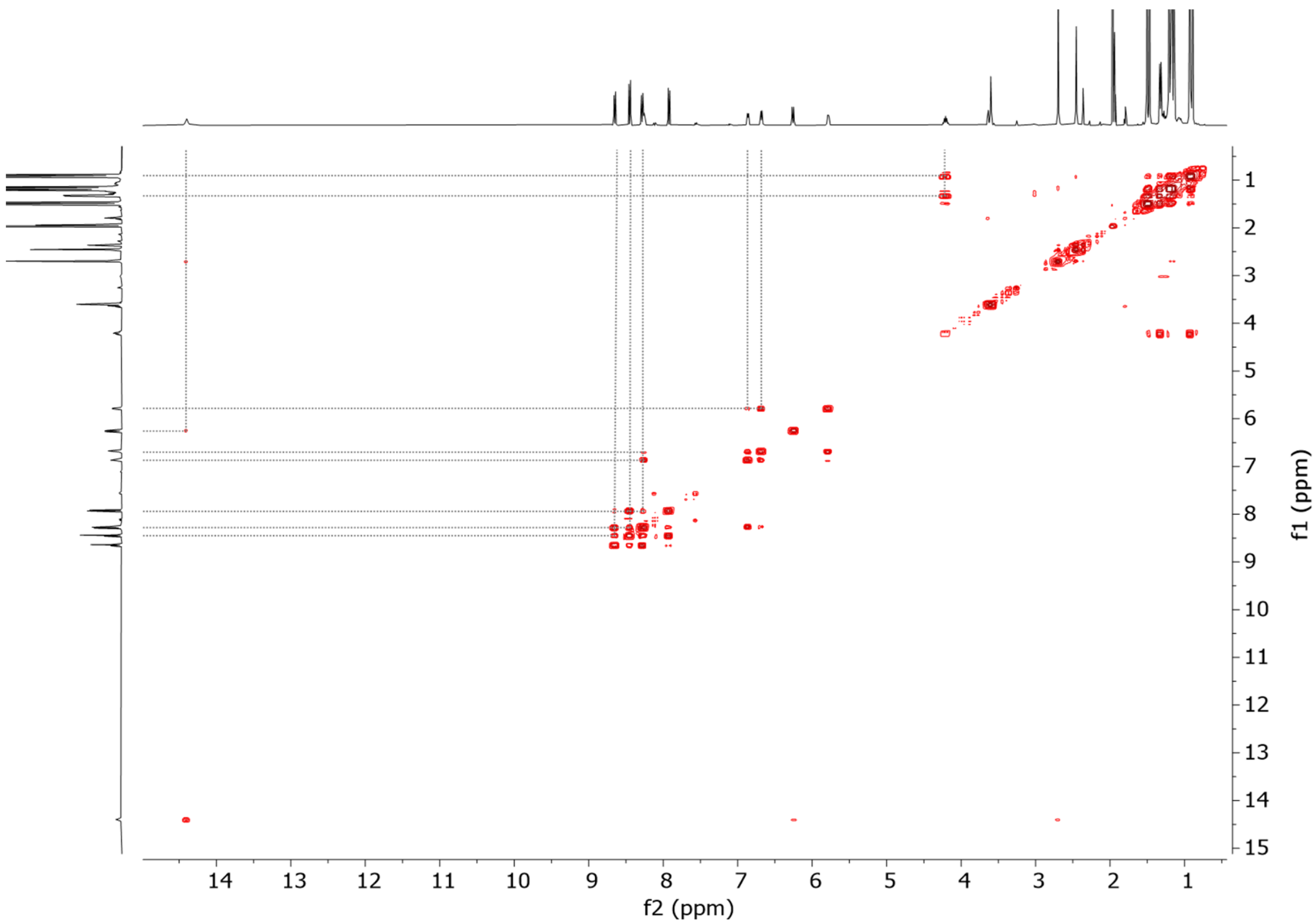

Figure S35. ^{1}H-^{1}H COSY NMR spectrum of **4** in CD_3CN at 298 K.

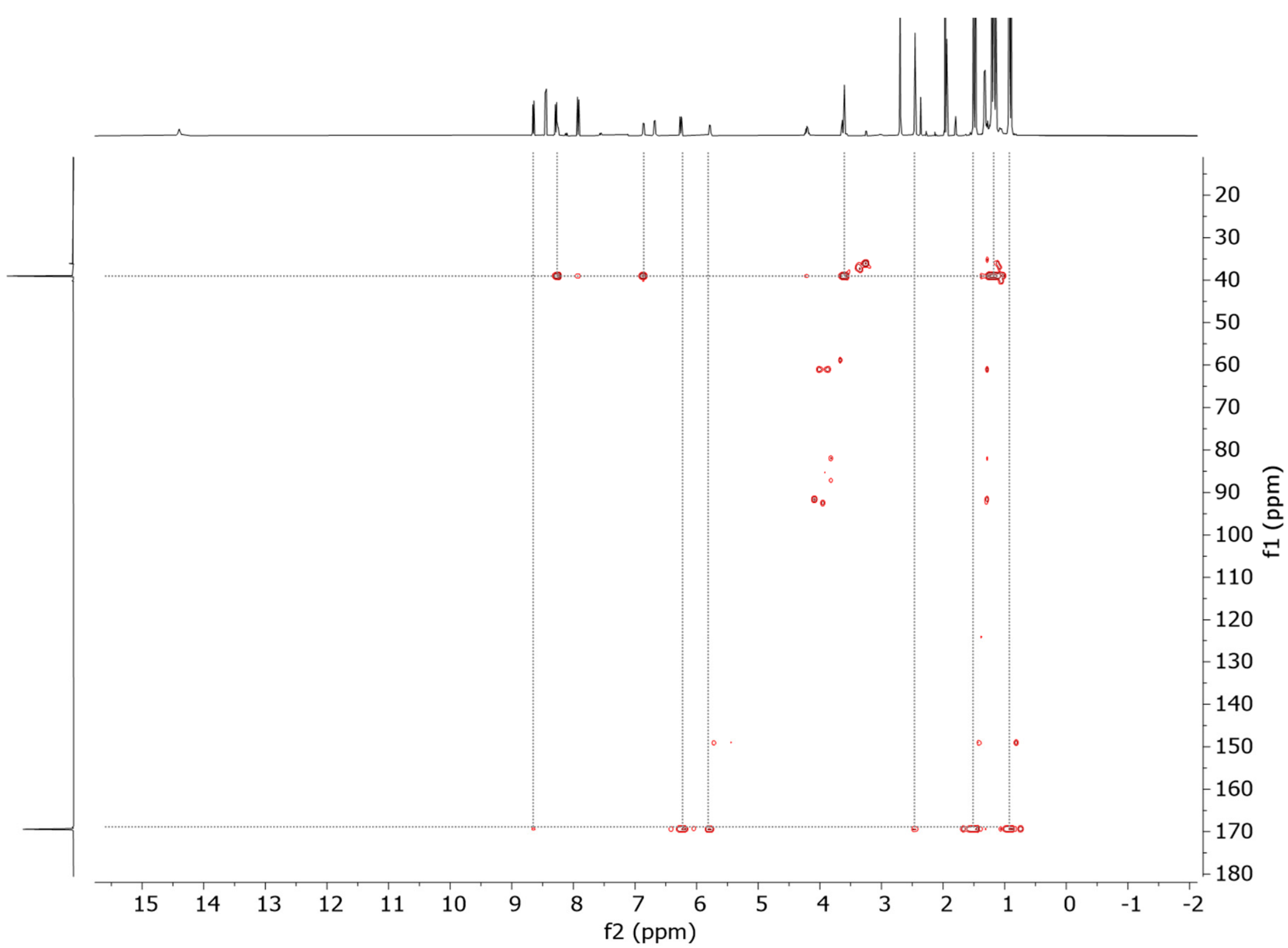

Figure S36. ^{1}H-^{31}P HMBC NMR spectrum of **4** in CD_3CN at 298 K.

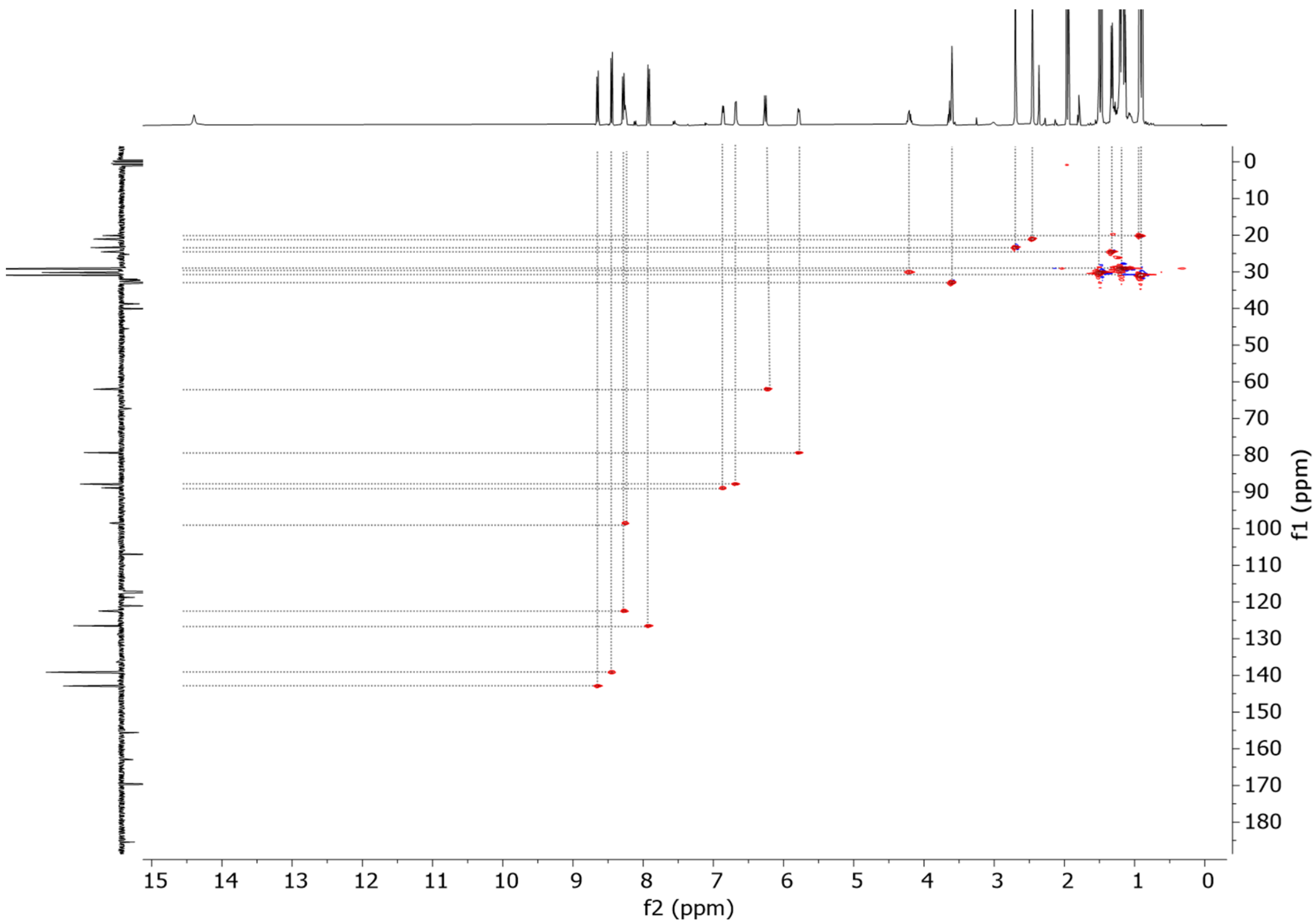

Figure S37. ^{1}H-^{13}C ASAP-HMQC NMR spectrum of **4** in CD_3CN at 298 K.

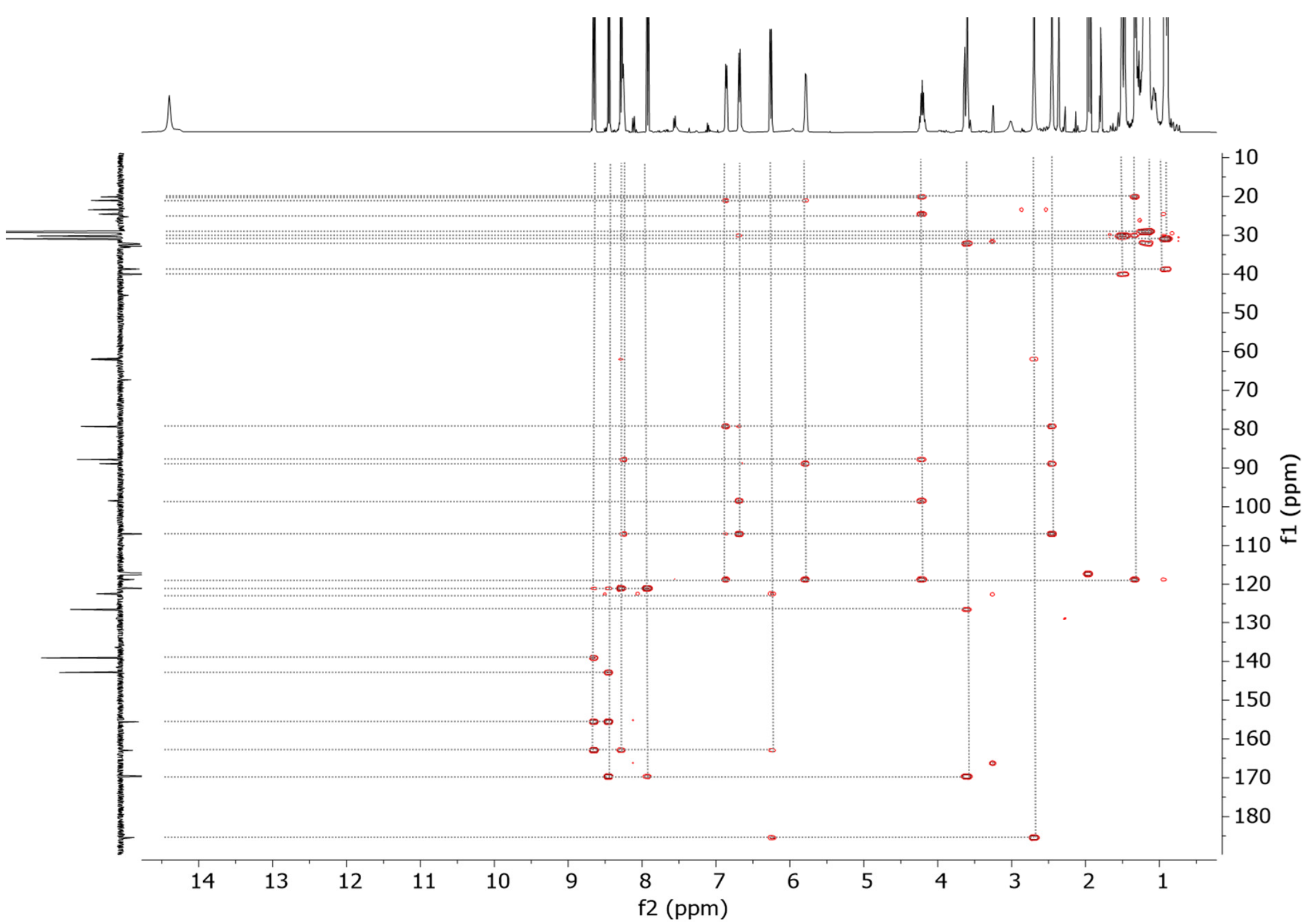

Figure S38. ^{1}H-^{13}C HMBC NMR spectrum of **4** in CD_3CN at 298 K.

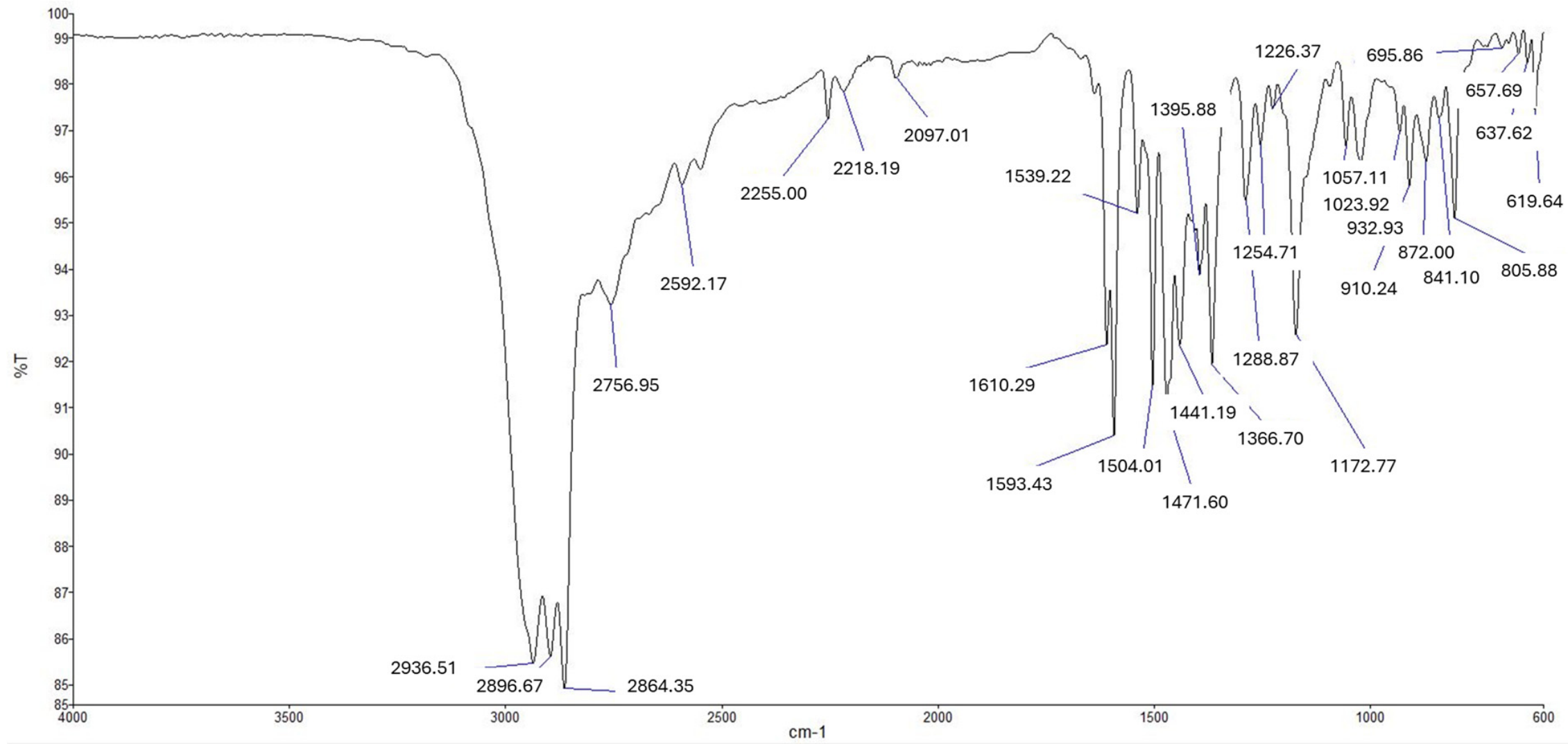

Figure S39. ATR-IR spectrum of **4** measured as a film under N_2 flow at 298 K.

S2.6. [PNNP^{2-MeCN}-$Ru_2Cl_2(MeCN)_2$] $2PF_6$ (5)

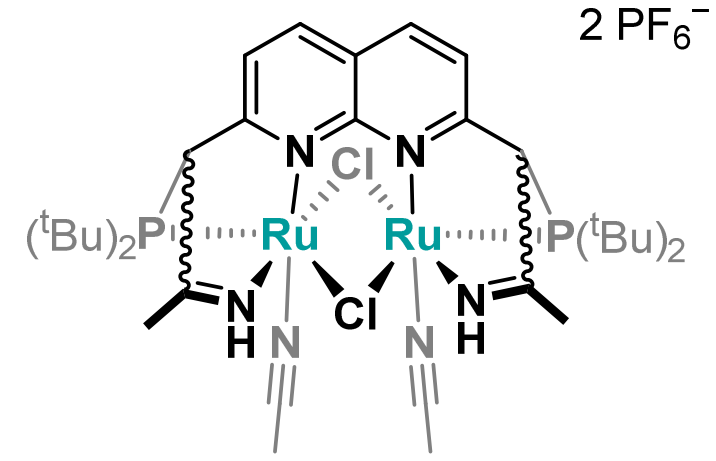

A 25 mL Schlenk bomb was charged with KPF_6 (36.8 mg, 0.2 mmol) dissolved in MeCN (2.0 mL), $^{t\text{-}Bu}$PNNP (44.6 mg, 0.1 mmol) dispersed in MeCN (3.0 mL) and $[Ru_2Cl_4(C_6H_6)_2]$ (50.0 mg, 0.1 mmol) dispersed in MeCN (3.0 mL) and a catalytic amount of Et_3N (10%, 1.5 µL). 4.0 mL more of MeCN was added to the Schlenk bomb, resulting in a brown suspension. The suspension was placed in an oil bath and stirred vigorously at 115 °C over the course of 36 h. By that time, the color of the reaction mixture had changed from brown to dark red. The mixture was allowed to cool to room temperature and transferred into a glovebox, where it was filtered to remove KCl. The mixture was subsequently filtered to remove KCl, after which all the volatiles were evaporated off under dynamic vacuum, resulting in a dark red solid as an inseparable mixture of the products (104.0 mg, 89.0 %).

<u>*Major species:*</u>

^{1}H NMR (400 MHz, CD_3CN, 298 K): δ = 10.63 (s, *broad*, 2H), 8.67 (d, $^3J_{H,H}$ = 7.8 Hz, 2H), 8.15 (d, $^3J_{H,H}$ = 8.1 Hz, 2H), 5.80 (dd, $^1J_{H,P}$ = 7.5, $^4J_{H,H}$ = 2.1 Hz, 2H), 2.69 (s, 6H), 2.47 (s, 6H), 1.44 (d, overlapping with the signal of the minor species), $^3J_{H,P}$ = 12.8 Hz, 18H), 0.90 (d, $^3J_{H,P}$ = 13.0 Hz, 18H).

^{13}C{^{1}H} NMR (100 MHz, CD_3CN, 298 K): δ = 181.6 (d, $^2J_{C,P}$ = 3.1 Hz), 169.5 (d, $^5J_{C,P}$ = 2.1 Hz), 166.2 (s), 143.2 (s), 130.6 (s), 125.4 (s), 124.1 (d, $^3J_{C,P}$ = 6.0 Hz, overlapping with the signal of the minor species), 67.3 (d, $^1J_{C,P}$ = 10.4 Hz), 40.0 (d, $^1J_{C,P}$ = 12.0 Hz), 39.3 (d, $^1J_{C,P}$ = 9.7 Hz), 31.2 (d, $^2J_{C,P}$ = 2.5 Hz), 30.6 (d, $^2J_{C,P}$ = 2.7 Hz), 25.0 (d, $^3J_{C,P}$ = 3.5 Hz), 4.6 (s).

^{31}P{^{1}H} NMR (162 MHz, CD_3CN, 298 K): δ = 150.0 (s, 2P), -144.0 (hept, $^1J_{P,F}$ = 706.5 Hz, 2P).

^{19}F{^{1}H} NMR (376 MHz, CD_3CN, 298 K): δ = −72.7 (d, $^1J_{F,P}$ = 706.5 Hz, 12F).

<u>*Minor species:*</u>

^{1}H NMR (400 MHz, CD_3CN, 298 K): δ = 10.74 (s, 2H), 8.59 (d, $^3J_{H,H}$ = 7.9 Hz, 2H), 8.06 (d, $^3J_{H,H}$ = 8.3 Hz, 2H), 5.69 (dd (overlapping doublets, $^1J_{H,P}$ = 4.7, $^4J_{H,H}$ = 2.2 Hz, 2H), 2.68 (s, 6H), 2.52 (s, 6H), 1.40 (d (overlapping with d of major species), $^3J_{H,P}$ = 13.6 Hz, 18H), 0.82 (d, $^3J_{H,P}$ = 12.8 Hz, 18H).

^{13}C{^{1}H} NMR (100 MHz, CD_3CN, 298 K): δ = 183.0 (d, $^2J_{C,P}$ = 2.9 Hz), 170.1 (s), 166.9 (s), 143.1 (s), 130.5 (s), 125.5 (s), 124.1 (d, $^3J_{C,P}$ = 6.0 Hz), 67.0 (d, $^1J_{C,P}$ = 9.5 Hz), 39.9 (d, $^1J_{C,P}$ = 12.0 Hz), 39.7 (d, $^1J_{C,P}$ = 9.4 Hz), 31.1 (d, $^2J_{C,P}$ = 2.0 Hz), 30.6 (d, $^2J_{C,P}$ = 1.7 Hz), 25.2 (d, $^3J_{C,P}$ = 3.1 Hz), 4.2 (s).

^{31}P{^{1}H} NMR (162 MHz, CD_3CN, 298 K): δ = 155.0 (s, 2P), -144.0 (hept, $^1J_{P,F}$ = 706.5 Hz, 2P).

^{19}F{^{1}H} NMR (376 MHz, CD_3CN, 298 K): δ = −72.7 (d, $^1J_{F,P}$ = 706.5 Hz, 12F).

Anal. Calcd. For $C_{34}H_{56}Cl_2F_{12}N_6P_4Ru_2$: C, 34.79; H, 4.81; N, 7.16. **Found:** C, 35.09; H, 5.12; N, 8.16. *The observed deviations in the found values are consistent with the presence of residual MeCN in the sample (also detected in the ^{1}H NMR spectrum, ~1 equiv; Figure S40). For comparison, inclusion of 1 equiv of MeCN gives calculated values in closer agreement with the experimental data:* **Anal. Calcd. For $C_{34}H_{56}Cl_2F_{12}N_6P_4Ru_2$ + 1 CH_3CN***:* C, 35.59; H, 4.90; N, 8.07.

ATR-IR (film, N_2 flow, 298 K): ν = 3303 (m), 2953 (m), 2908 (m), 2875 (m), 2284 (w), 2251 (w), 1670 (w), 1615 (m), 1593 (m), 1515 (w), 1482 (m), 1467 (m), 1437 (m), 1372 (m), 1291 (w), 1217 (w), 1176 (m), 1038 (w), 829 (s), 739 (w), 631 (w) cm^{-1}.

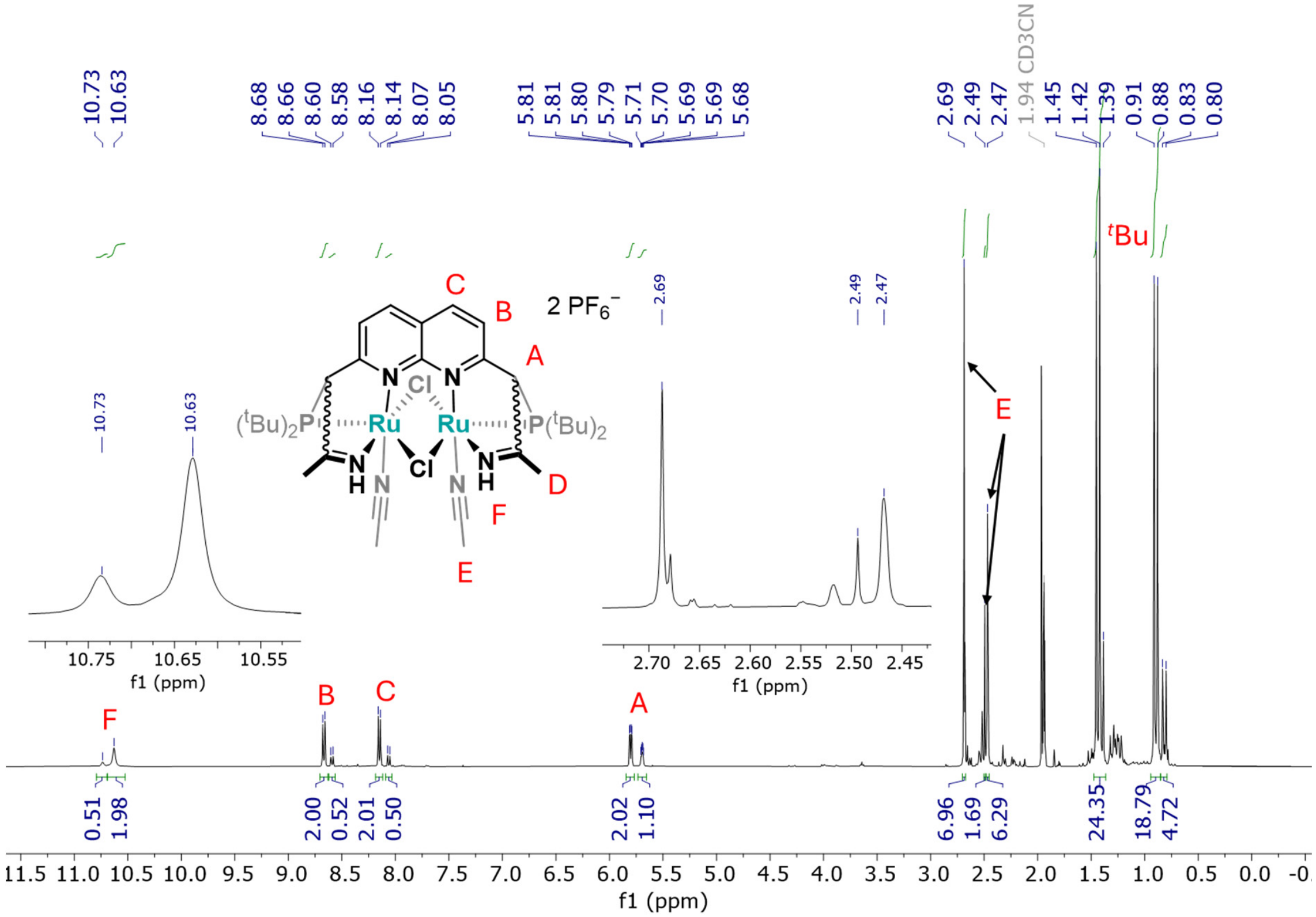

Figure S40. ^{1}H NMR spectrum of the mixture of the diastereomers of **5** in CD_3CN at 298 K.

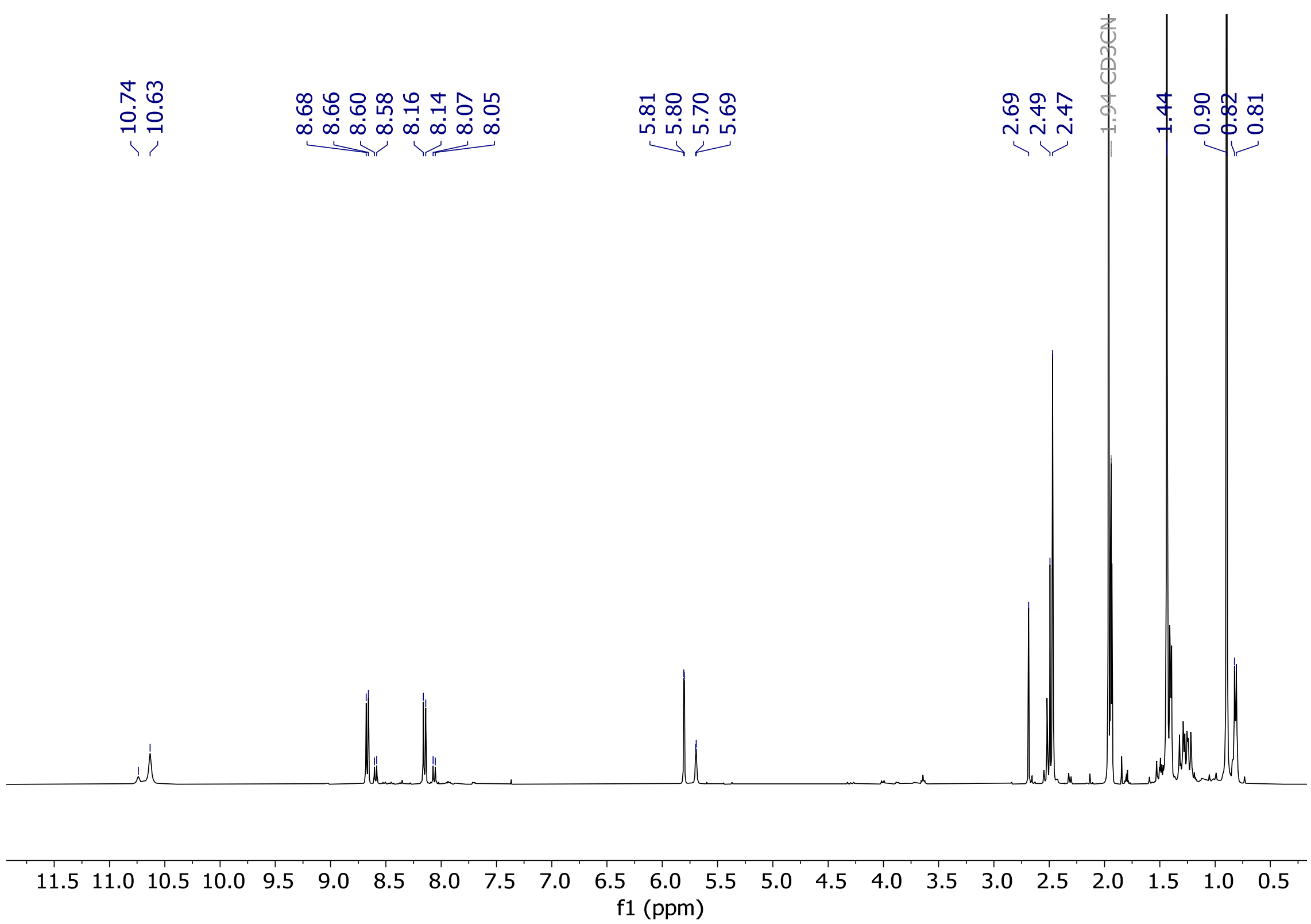

Figure S41. $^1H\{^{31}P\}$ NMR spectrum of the mixture of the diastereomers of **5** in CD_3CN at 298 K.

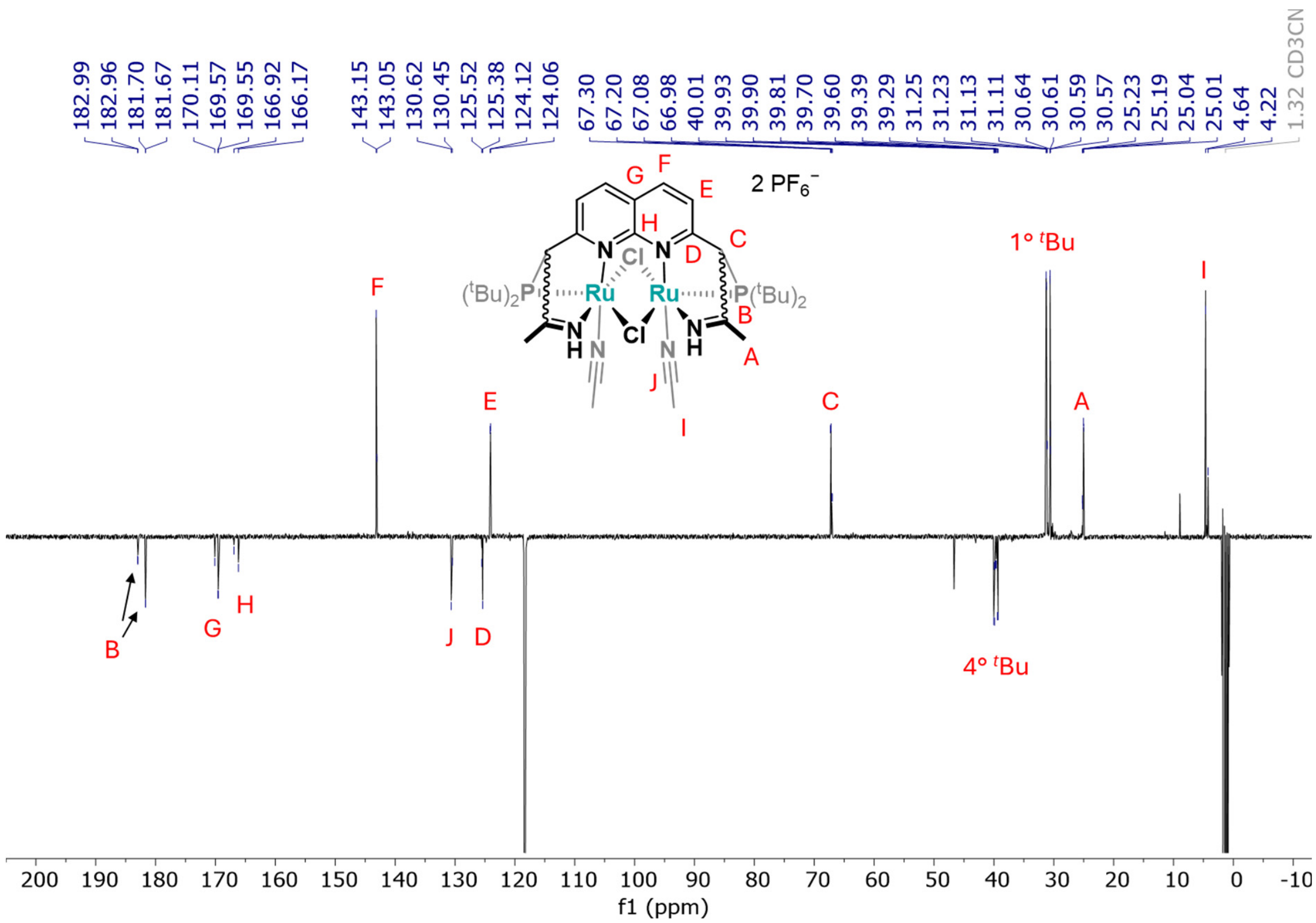

Figure S42. $^{13}C\{^{1}H\}$ APT NMR spectrum of the mixture of the diastereomers of **5** in CD_3CN at 298 K.

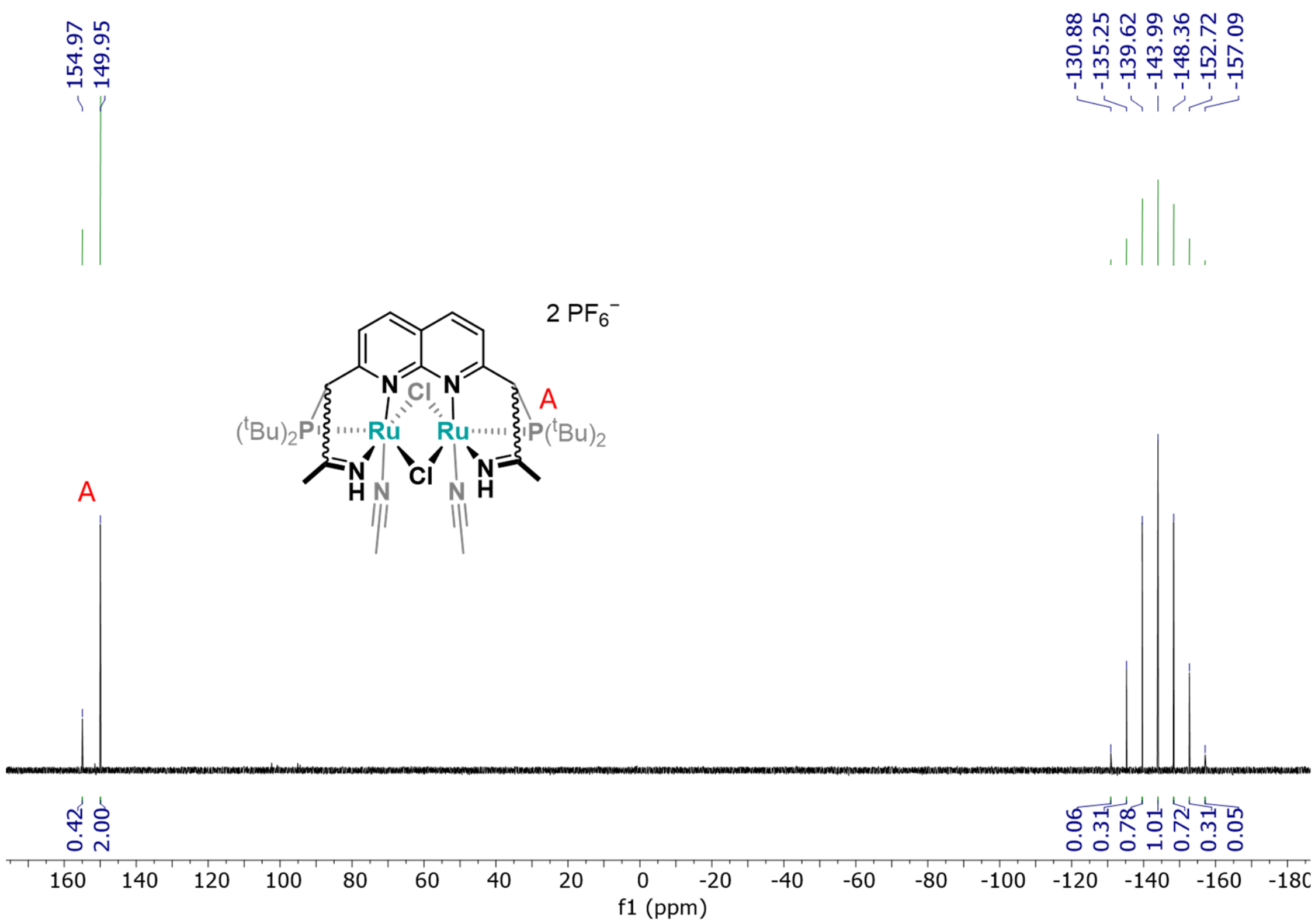

Figure S43. $^{31}P\{^{1}H\}$ NMR spectrum of the mixture of the diastereomers of **5** in CD_3CN at 298 K.

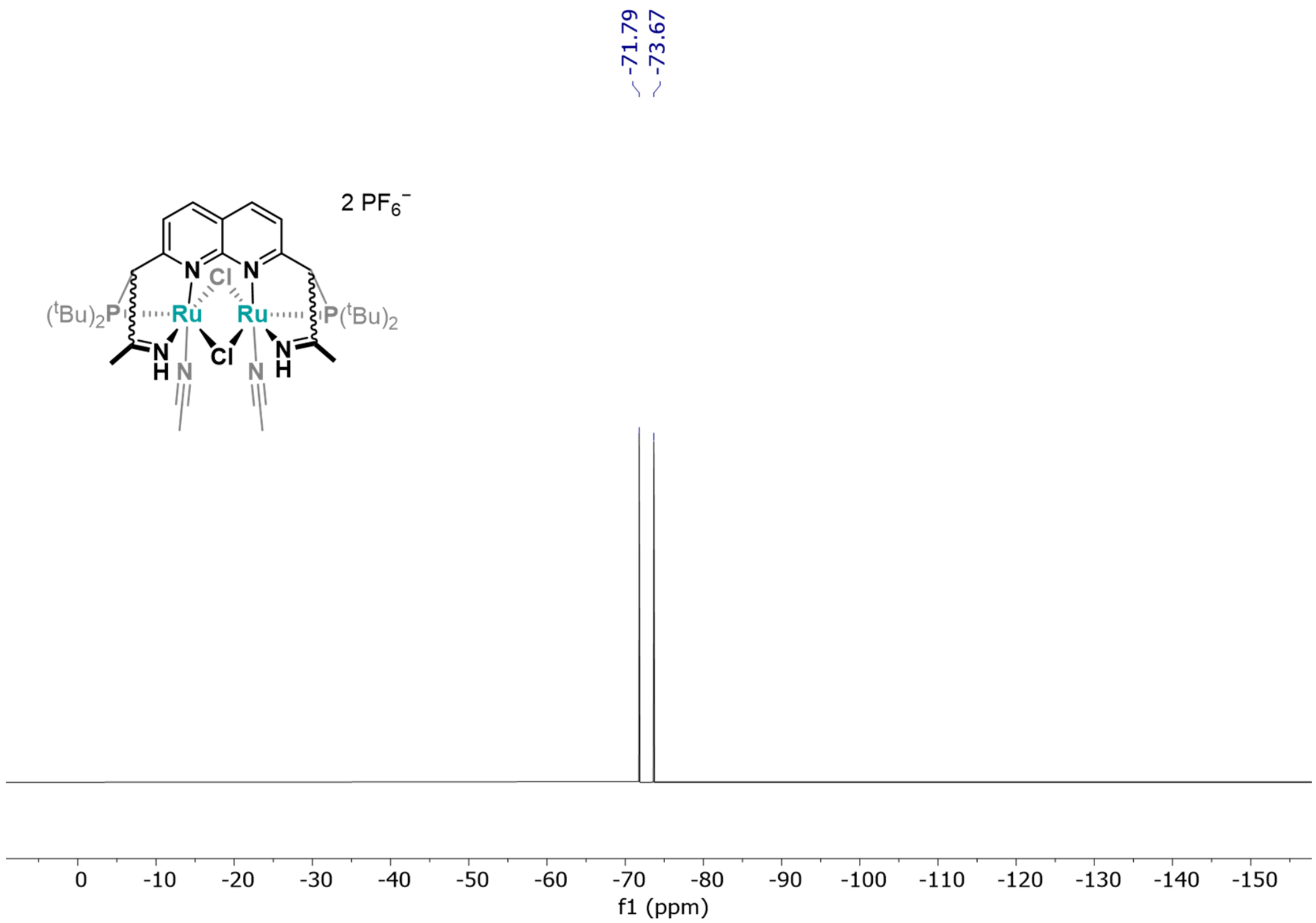

Figure S44. $^{19}F\{^{1}H\}$ NMR spectrum of the mixture of the diastereomers of **5** in CD_3CN at 298 K.

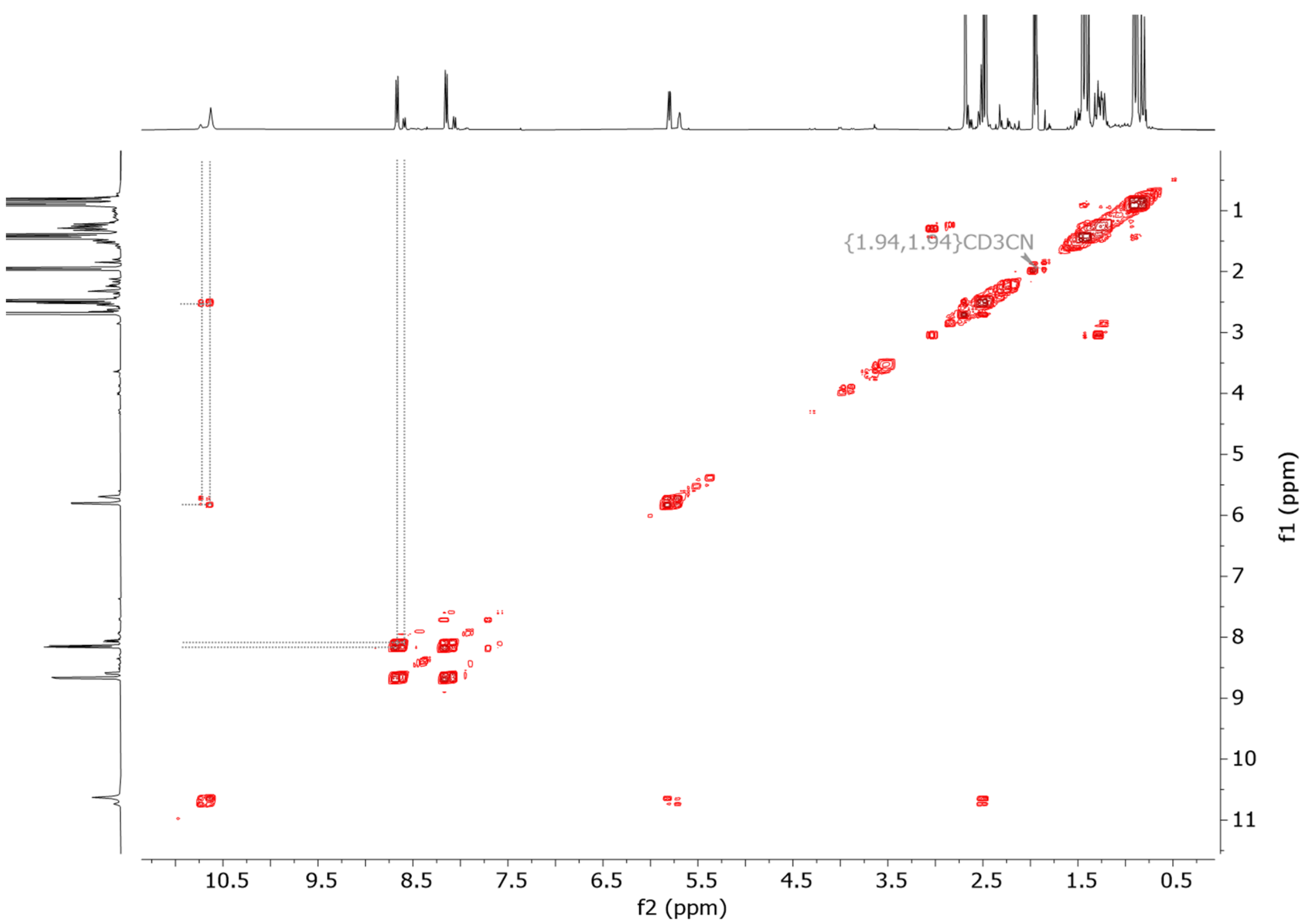

Figure S45. ^{1}H-^{1}H COSY NMR spectrum of the mixture of the diastereomers of **5** in CD_3CN at 298 K.

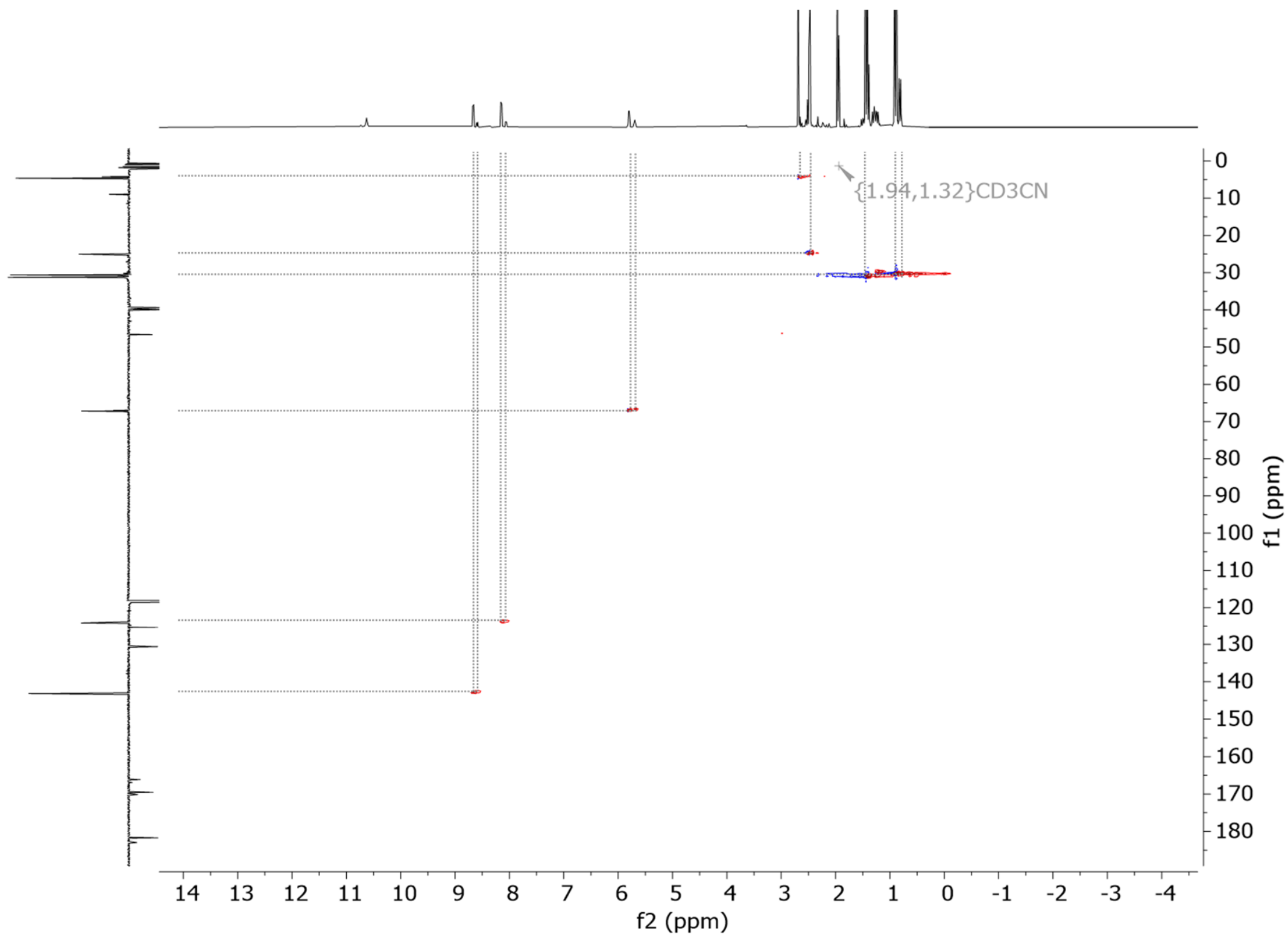

Figure S46. ^{1}H-^{13}C ASAP-HMQC NMR spectrum of the mixture of the diastereomers of **5** in CD_3CN at 298 K.

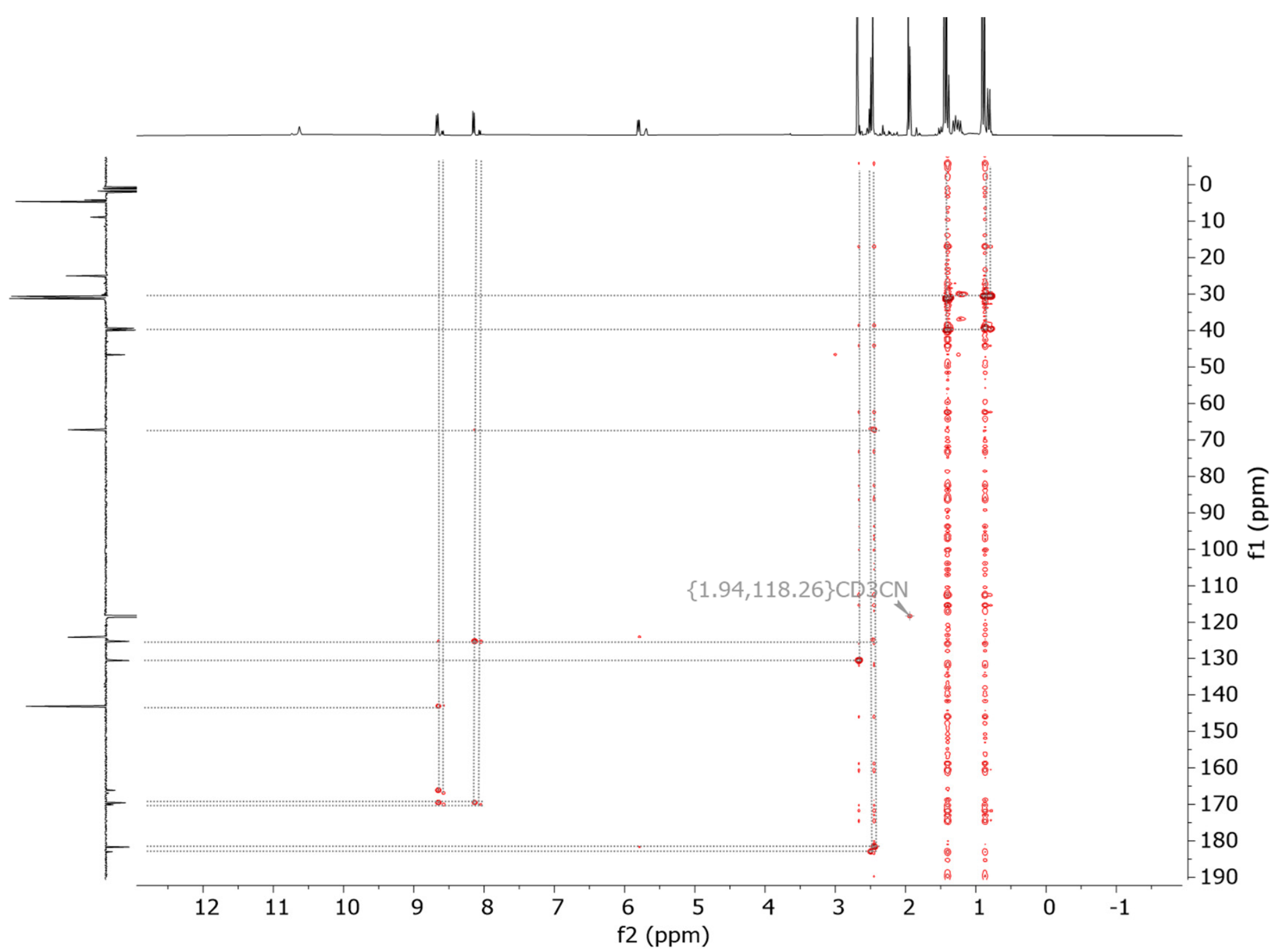

Figure S47. ^{1}H-^{13}C HMBC NMR spectrum of the mixture of the diastereomers of **5** in CD_3CN at 298 K.

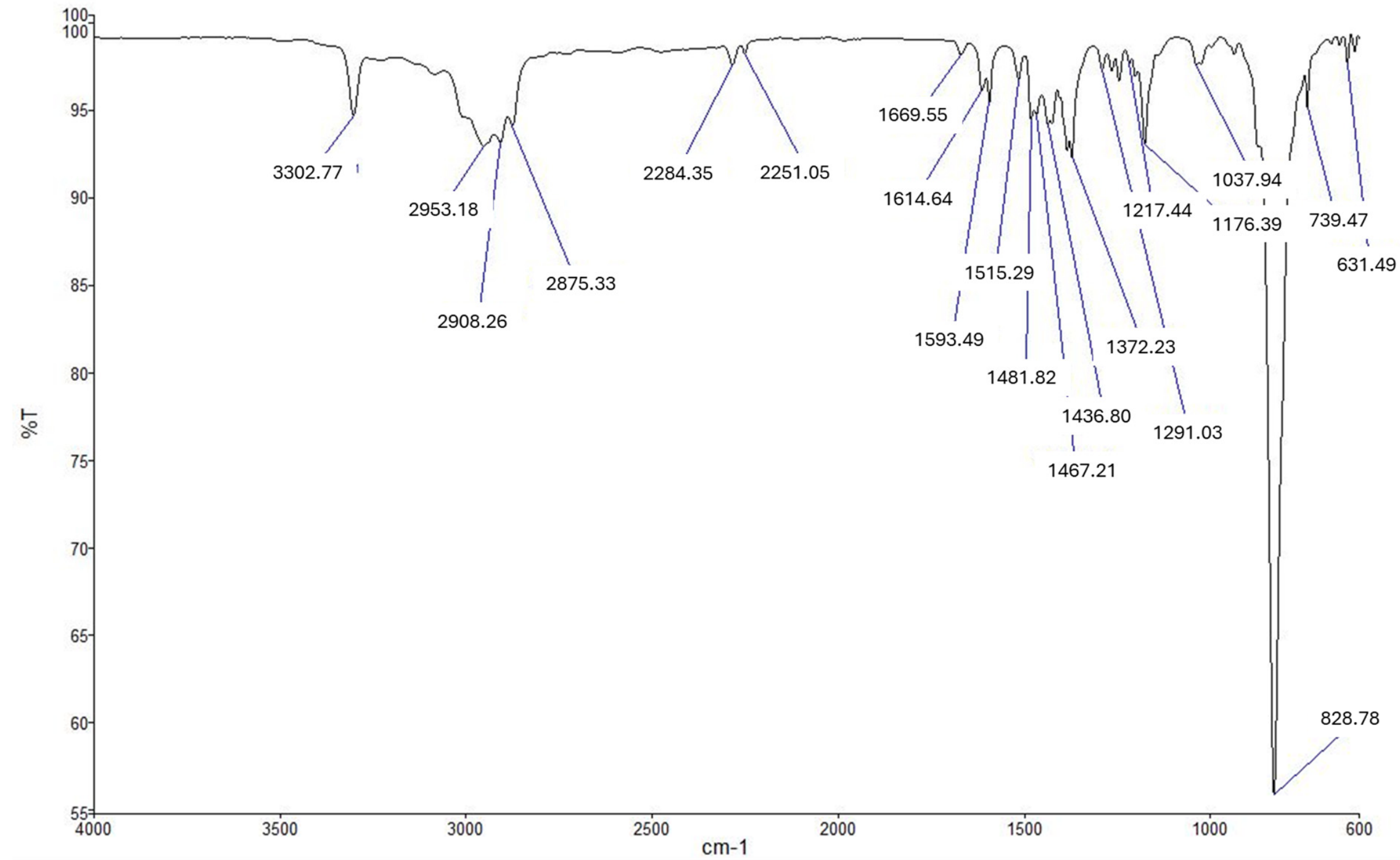

Figure S48. ATR-IR spectrum of the mixture of the diastereomers of **5** measured as a film under N_2 flow at 298 K.

3. Isotopic Labelling Experiments

S3.1. PNNP-d_4

The procedure is adapted from one for analogous the original PNNP ligand synthesis[1] *and deuteration of analogous PN ligand.*[3]

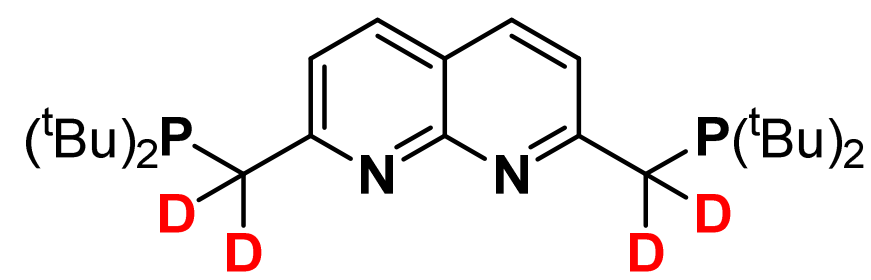

A 100 mL Schlenk tube was charged with ^{tBu}PNNP (150.0 mg, 0.34 mmol), KO^{t}Bu (75.4, 0.67 mmol), 1.0 mL MeOD and 10.0 mL THF to give a yellow solution. The resulting mixture was refluxed for 16 hours. Next, the yellow mixture was extracted with DCM (3x5 mL). The extracts were combined, dried over Na_2SO_4 and concentrated in vacuum to yield a white-off solid, which was transferred into a N_2-filled glovebox. The solid was extracted by vigorous stirring with *n*-hexane (5 mL, 20 min), and THF (5 mL, 10 min). The residual solid was dried in vacuum to give a white solid (112.0 g, 74%).

^{1}H NMR (61 MHz, CH_2Cl_2, 298 K): δ = 8.00 (d, $^3J_{H,H}$ = 8.4 Hz, 2H), 7.58 (d, $^3J_{H,H}$ = 8.2 Hz, 2H), 3.22 (s, 0.1H*), 1.15 (d, $^2J_{H,P}$ = 11.3 Hz, 36H).

^{2}H NMR (61 MHz, CH_2Cl_2, 298 K): δ = 3.2 (s, broad, 4D).

Note: ^{1}H NMR spectrum is analogous to the non-deuterated PNNP ligand.[1] **The methylene positions of the ligand are deuterated (~95% "D").*

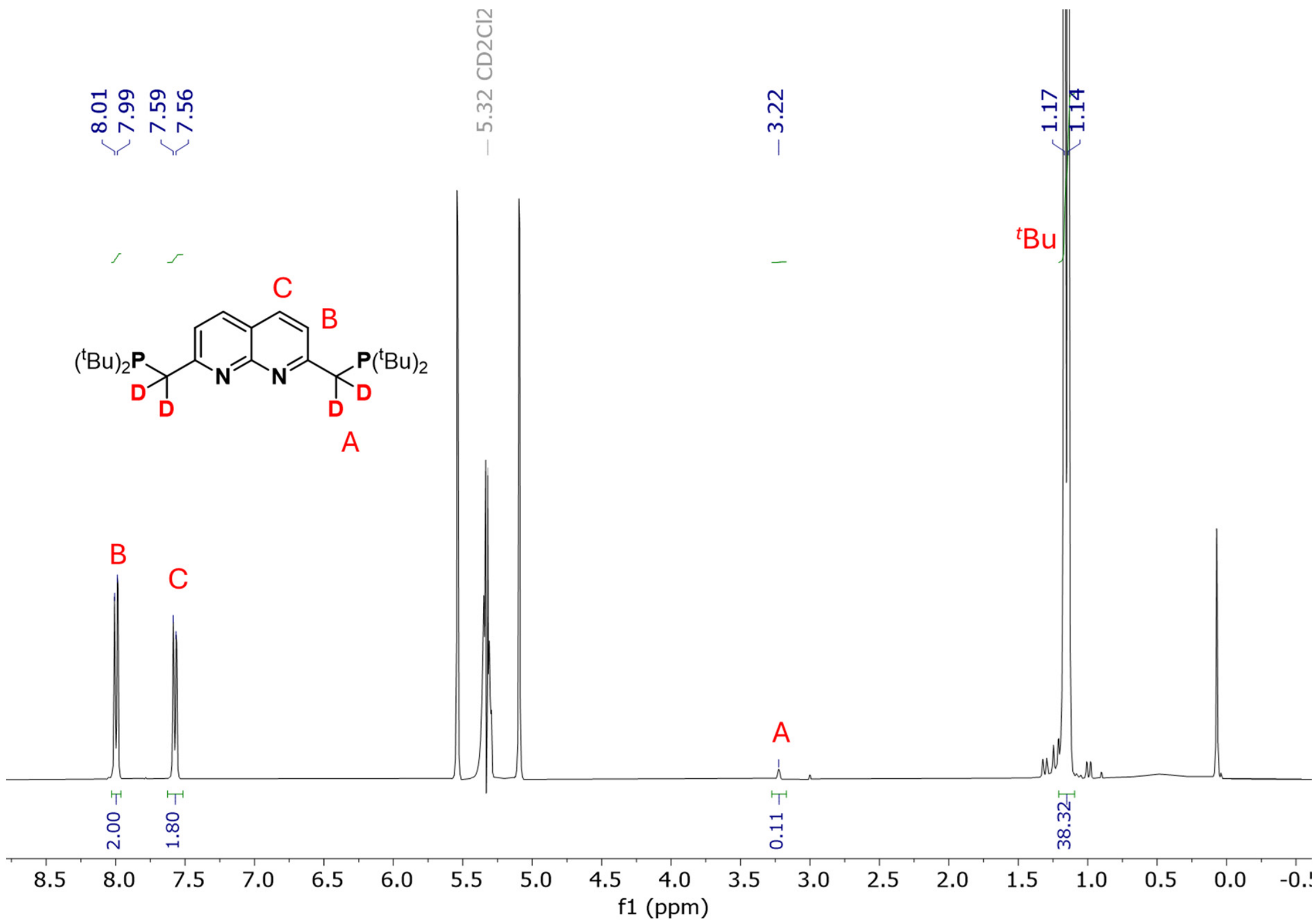

Figure S49. ^{1}H NMR spectrum of **PNNP-*d*$_4$** in CH_2Cl_2 (using the PRESAT function) at 298 K.

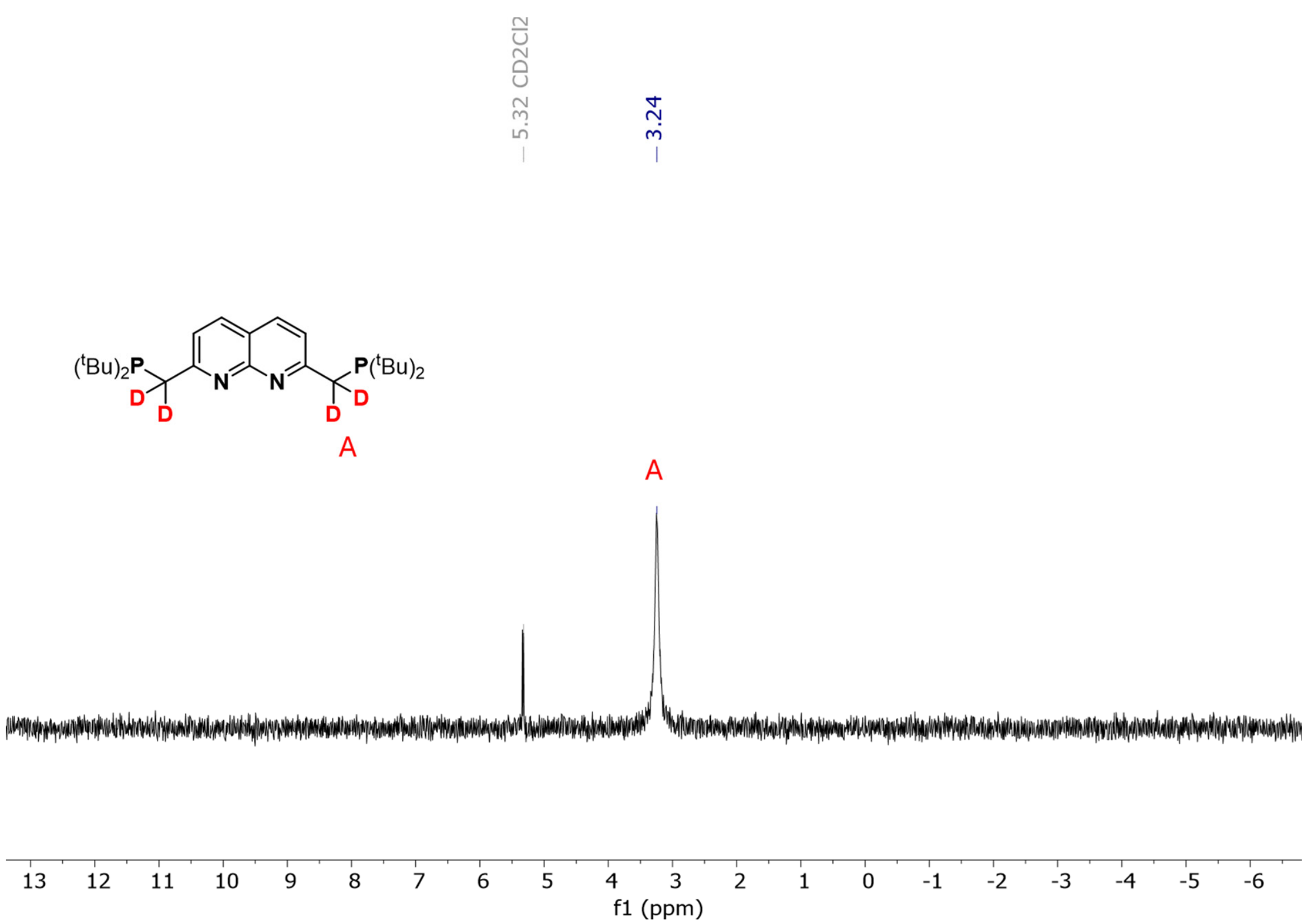

Figure S50. ^{2}H NMR spectrum of **PNNP-*d*$_4$** in CH_2Cl_2 at 298 K.

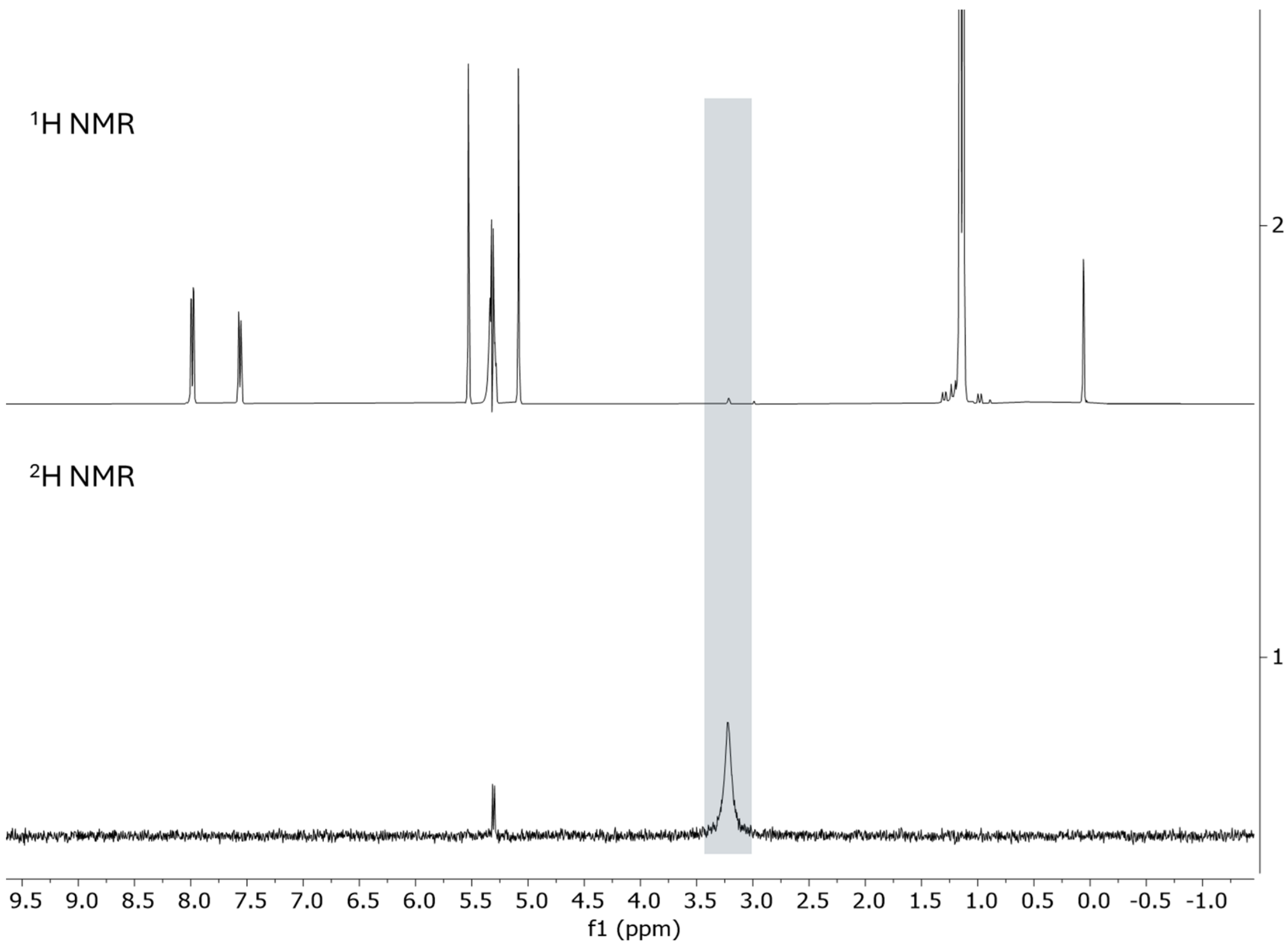

Figure S51. The overlap of ^{1}H NMR and ^{2}H NMR spectra of **PNNP-*d*$_4$** in CH_2Cl_2 at 298 K.

S3.2. [PNNP-RuCl(*p*-cymene)] Cl (2-D)

*Similarly to the synthesis of non-deuterated analogue **2**.*

A 20 mL glass vial was charged with **PNNP-*d*$_4$** (90.1 mg, 0.20 mmol), and [Ru_2Cl_4(*p*-cymene)$_2$] (61.2 mg, 0.10 mmol). Next, DCM (10.0 mL) was added to give a clear red solution. The reaction mixture was kept stirring in a glovebox at RT for 2 h, and the color of the reaction mixture became dark red. The mixture was filtered through a glass filter to give a red solution. All the volatiles were removed under a dynamic vacuum giving 121.1 mg (80%) of red powder.

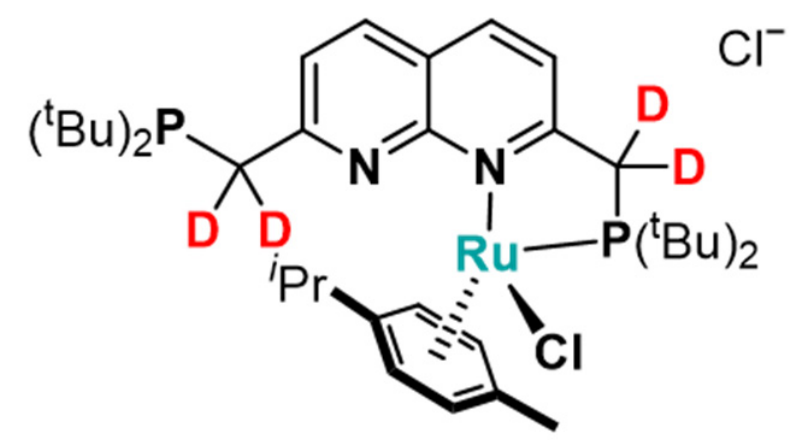

^{1}H NMR (400 MHz, CD_2Cl_2, 298 K): *the spectroscopic features are analogous to complex **2**. However, due to the fluxional process in solution, the exact resonance assignment is difficult. The spectrum obtained at 40 °C shows the significant sharpening as well as coalescence of the signals into one set of signals. Moreover, due to deuterated methylene positions, the signals associated with those positions (δ = 4.5 – 3.5 ppm) are absent in the ^{1}H spectra.*

The deuteration percentages: 94% D at the methylene linker of the(ruthenium side) and 96% D at the methylene linker (free side)

^{31}P{^{1}H} NMR (162 MHz, CD_2Cl_2, 298 K): δ = 84.7 (s, broad), 83.3 (s, broad), 37.0 (s, broad), 36.4 (s, broad). *Similarly to complex **2**, due to the fluxional process in solution, four ^{31}P resonances are observed at RT. The spectrum obtained at 40 °C shows the coalescence of the signals into one set of singlets.*

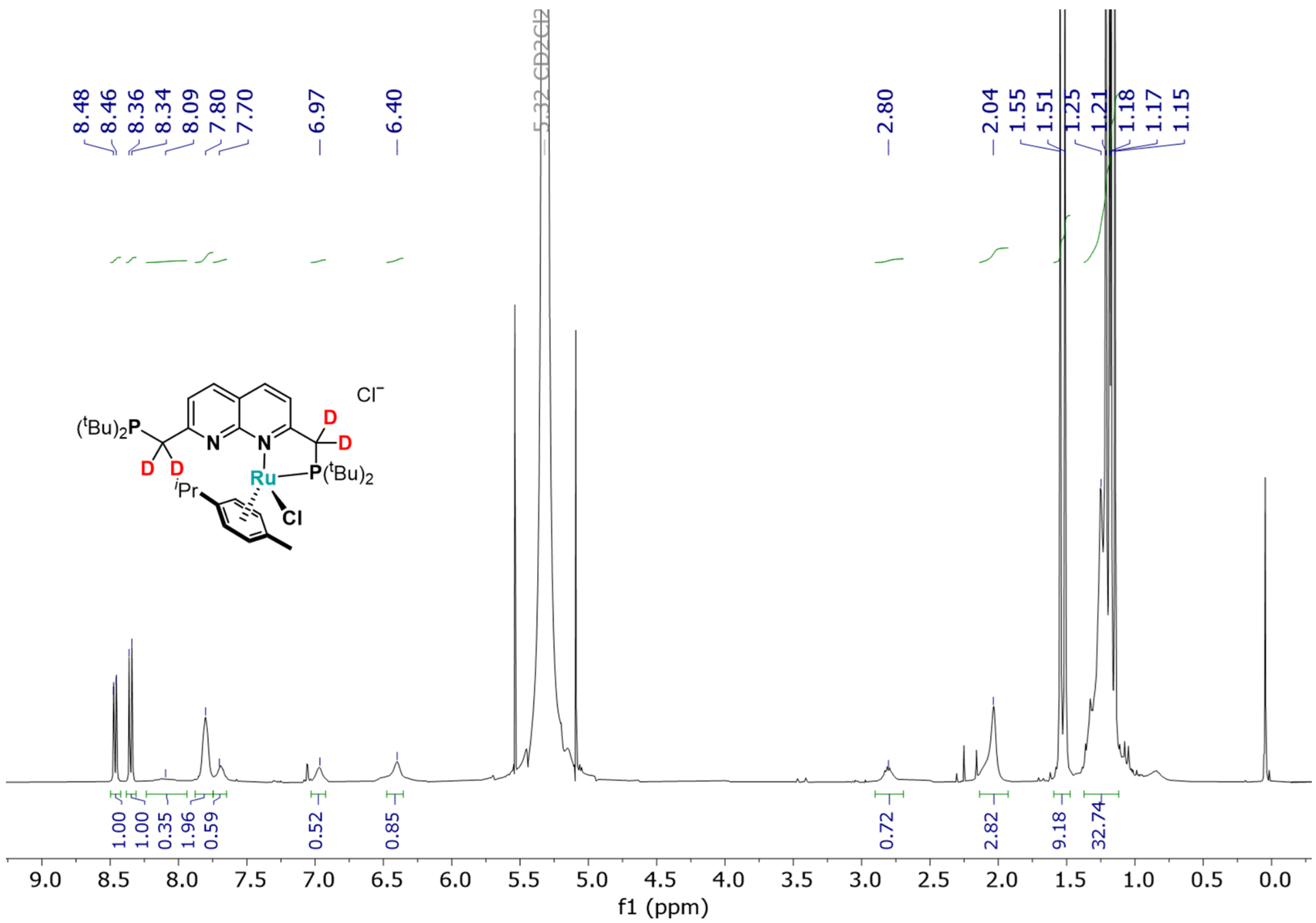

Figure S52. ^{1}H NMR spectrum of **2-D** in CH_2Cl_2 at 298 K. *The methylene protons at δ = 4.5 – 3.5 ppm are absent compared to* ***2****.*

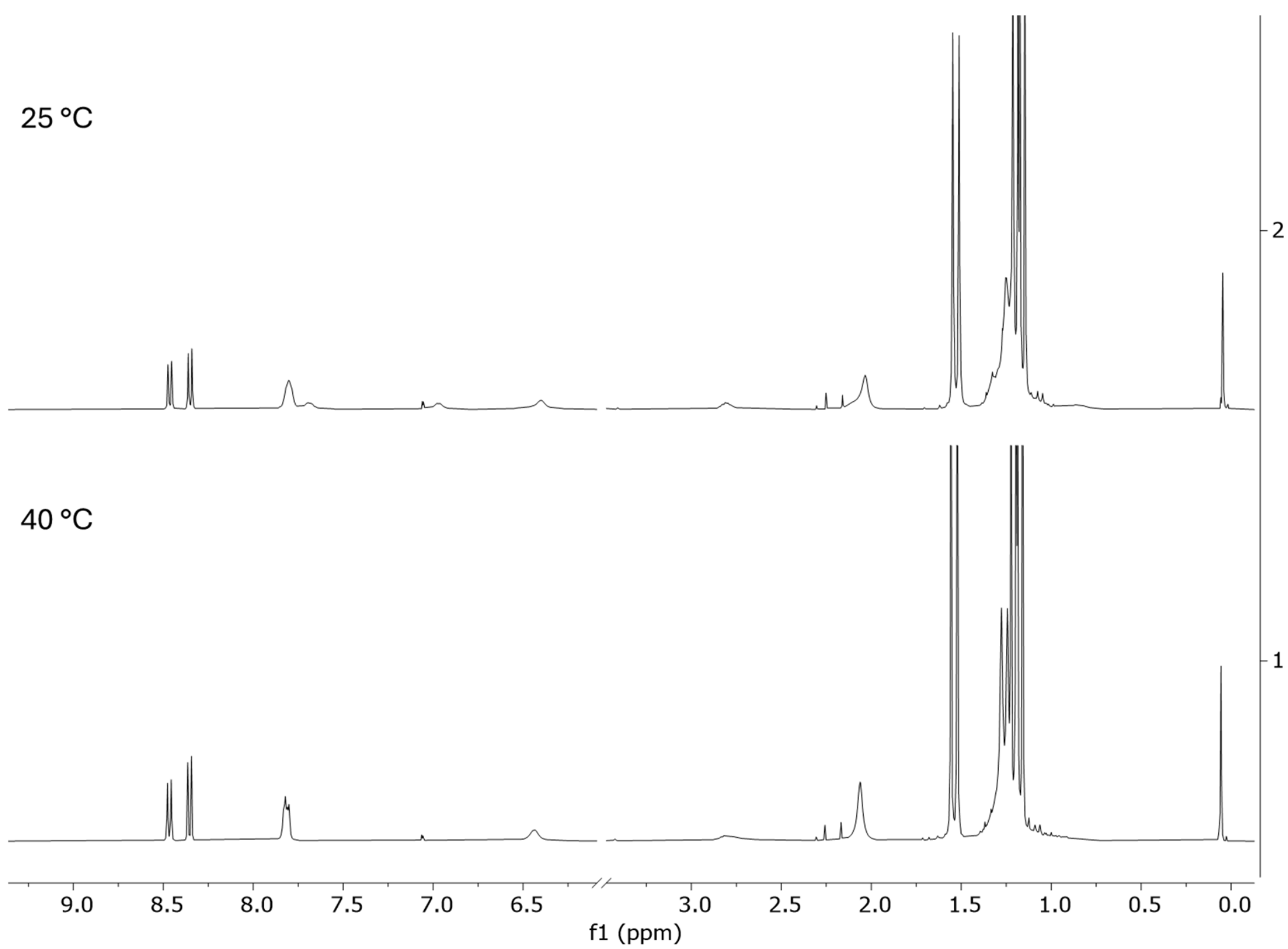

Figure S53. Stacked ^{1}H NMR spectra of **2-D** in CH_2Cl_2 at 298 K (*top*) and 313 K (*bottom*).

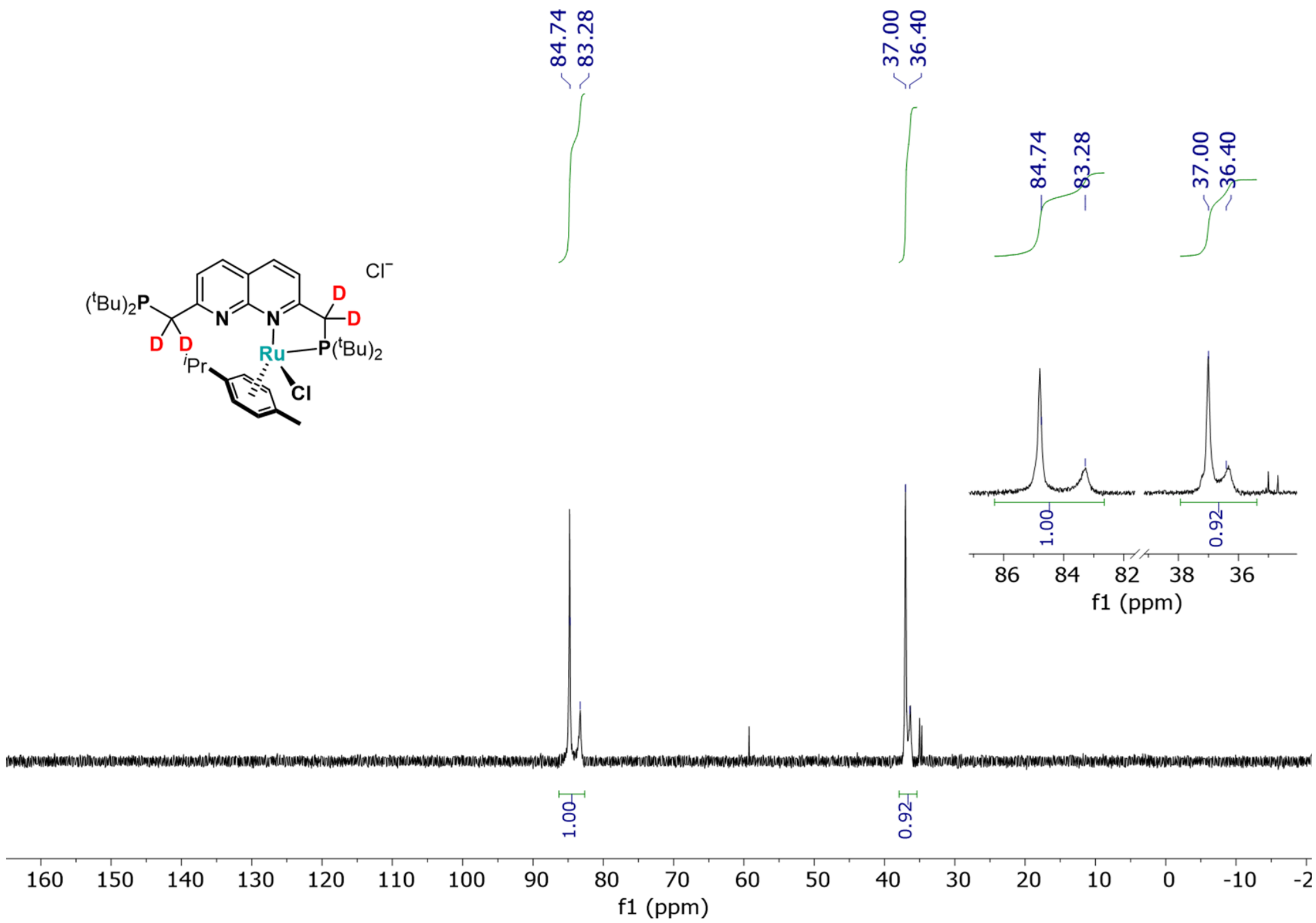

Figure S54. $^{31}P\{^1H\}$ NMR spectrum of **2-D** in CH_2Cl_2 at 298 K.

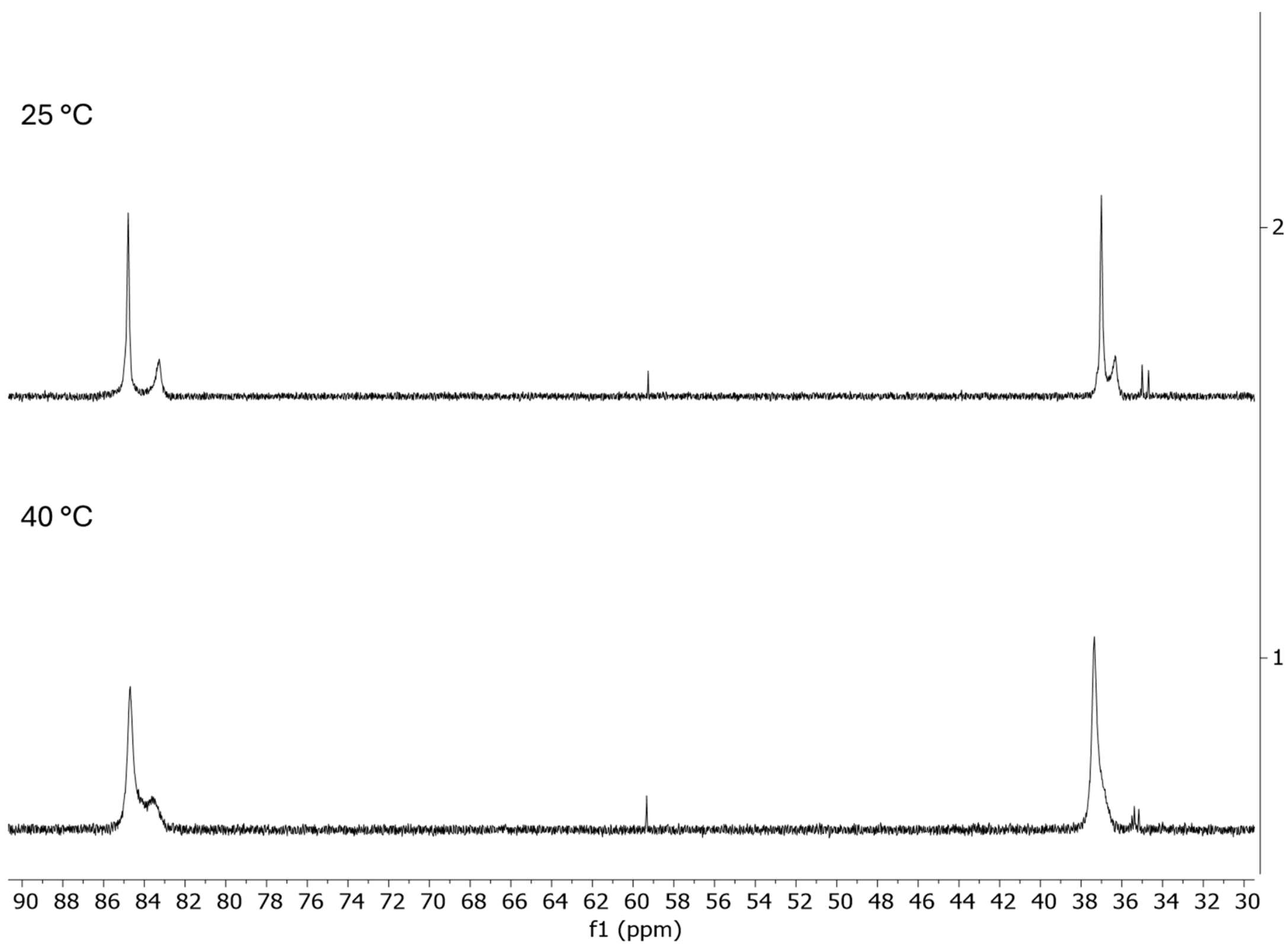

Figure S55. Stacked $^{31}P\{^1H\}$ NMR spectra of **2-D** in CH_2Cl_2 at 298 K (*top*) and 313 K (*bottom*). *Similar coalescence as for non-deuterated complex **2** is observed.*

S3.3. [PNNP-d_3^{MeCN}-RuCl(*p*-cymene)] 2Cl (4-D^{PNNP})

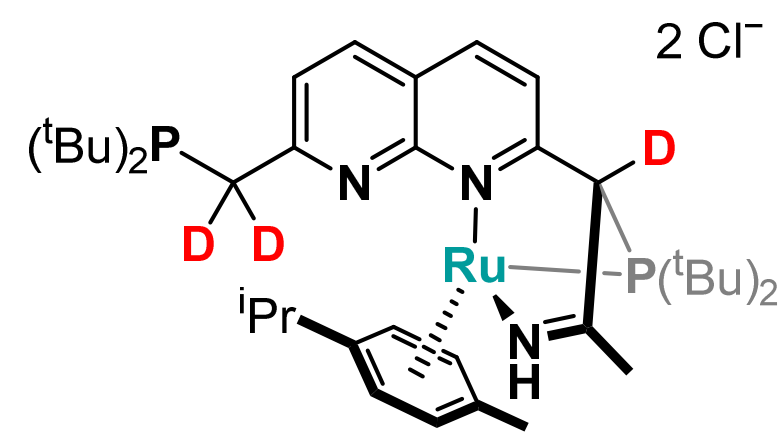

A 20 mL glass vial was charged with **2-D** (37.8 mg, 0.05 mmol), followed by addition of MeCN (7.0 mL) to give an orange solution. Next, a catalytic amount of Et_3N was added (10%, 1.0 mL of a 5 mM MeCN solution). The reaction mixture was kept stirring in a glovebox at RT for 2 h, and the color of the reaction mixture became red with a substantial amount of yellow precipitate formed. The mixture was filtered through a small glass frit to give a yellow residue. It was washed with THF (5.0 mL) and dried under dynamic vacuum to give the product as a yellow powder (36.3 mg, 91%).

^{1}H NMR (400 MHz, CD_3CN, 298 K): 14.36 (s, 1H, broad), 8.72 (d, $^3J_{H,H}$ = 8.1 Hz, 1H), 8.50 (d, $^3J_{H,H}$ = 8.4 Hz, 1H), 8.43 (d, $^3J_{H,H}$ = 8.1 Hz, 1H), 8.30 – 8.23 (m, 1H), 7.92 (d, $^3J_{H,H}$ = 8.2 Hz, 1H), 6.86 (d, $^3J_{H,H}$ = 6.2 Hz, 1H), 6.67 (d, $^3J_{H,H}$ = 6.4 Hz, 1H), 6.58 (d, $^1J_{H,P}$ = 8.3 Hz, 0.1H*), 5.80 (d, $^3J_{H,H}$ = 5.1 Hz, 1H), 4.20 (hept, $^3J_{H,H}$ = 6.2 Hz, 1H), 3.61 – 3.56 (m, 0.1H), 2.70 (d, $^4J_{H,H}$ = 1.2 Hz, 3H), 2.45 (s, 3H), 1.49 (d, $^3J_{H,P}$ = 13.4 Hz, 9H), 1.31 (d, $^3J_{H,H}$ = 6.9 Hz, 3H), 1.18 (d, $^3J_{H,P}$ = 11.0 Hz, 9H), 1.15 (d, $^3J_{H,P}$ = 11.0 Hz, 9H), 0.91 (d, $^3J_{H,H}$ = 13.4 Hz, 3H), 0.90 (d, $^3J_{H,P}$ = 8.0 Hz, 9H).

^{2}H NMR (61 MHz, CD_3CN, 298 K): δ = 6.5 (s, 1D, broad), 3.6 (s, 2D, broad).

**Note: methine and methylene positions of the ligand are deuterated.*

The deuteration percentages: 90% D at the CH (ruthenium side) and 94% D at the methylene linker (free side)

ATR-IR (film, N_2 flow, 298 K): ν = 2939 (s), 2897 (s), 2864 (s), 2748 (w), 2575 (w), 2255 (w), 2151 (w), 1612 (m), 1593 (m), 1535 (m), 1503 (m), 1471 (m), 1439 (m), 1394 (m), 1366 (m), 1289 (w), 1252 (w), 1173 (m), 1093 (w), 1058 (w), 1024 (w), 934 (w), 880 (w), 807 (w), 697 (w), 634 (w), 609 (w), 549 (w) cm^{-1}.

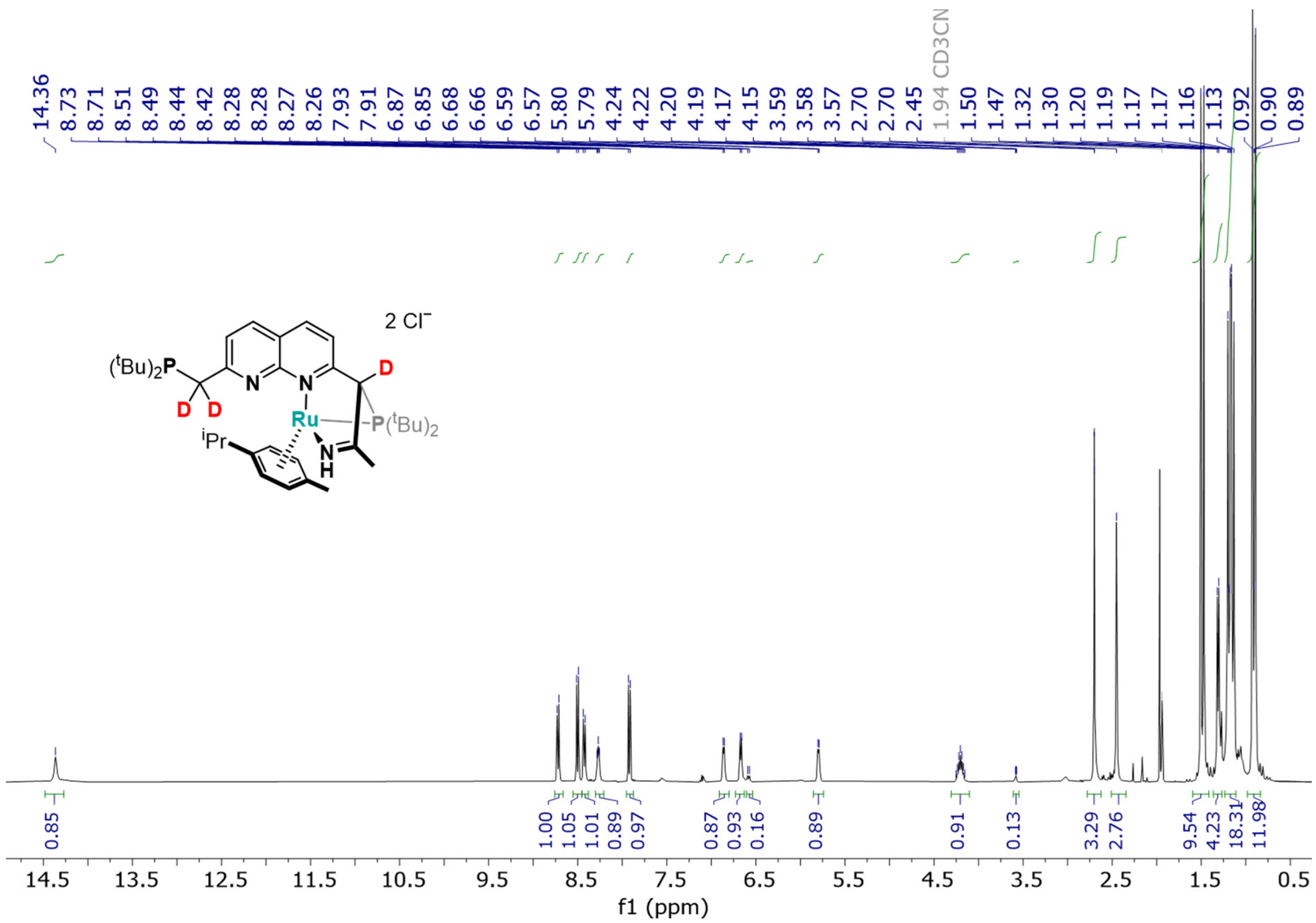

Figure S56. ^{1}H NMR spectrum of **4-D^{PNNP}** in CD_3CN at 298 K.

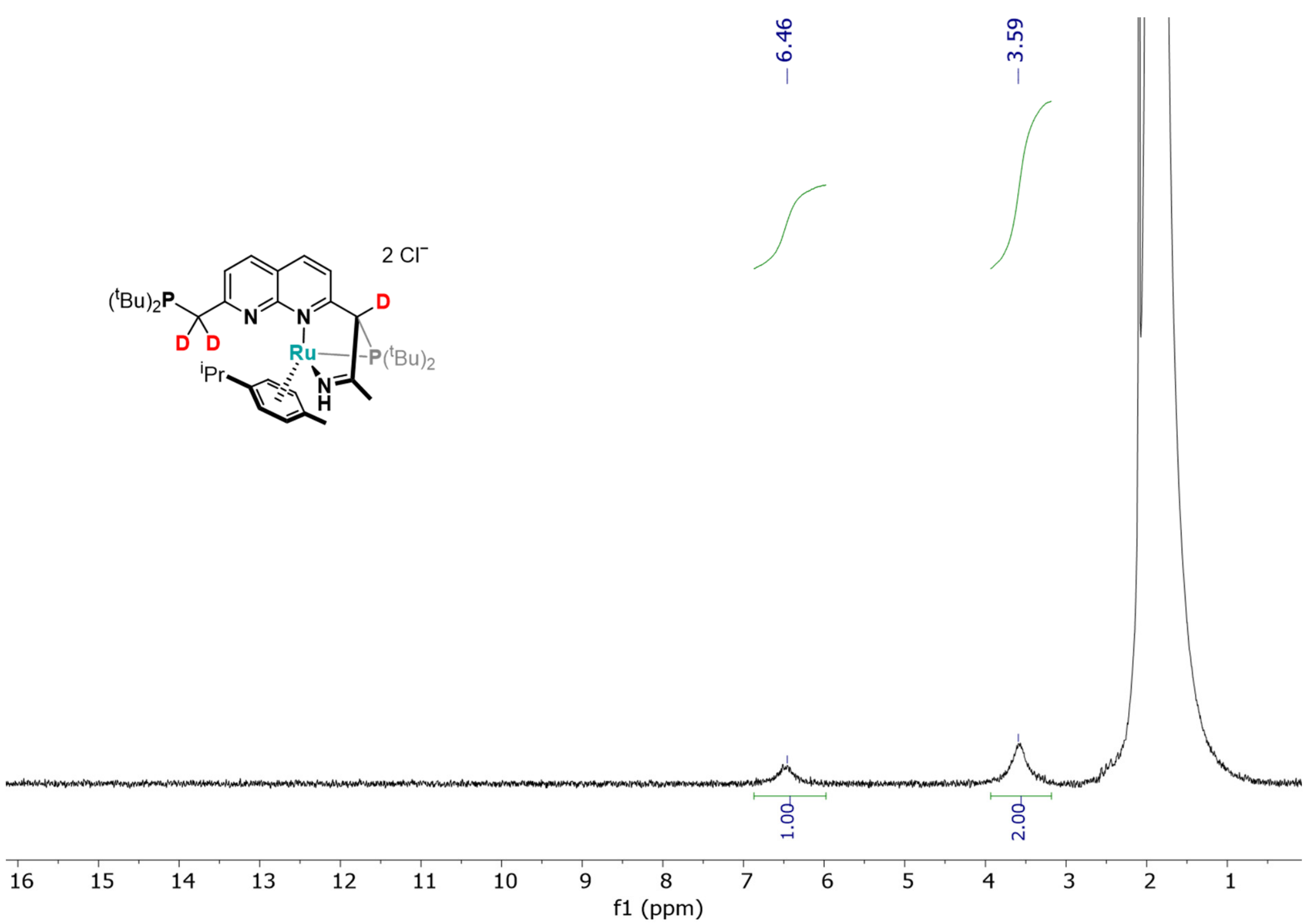

Figure S57. ^{2}H NMR spectrum of **4-D^{PNNP}** in CD_3CN at 298 K.

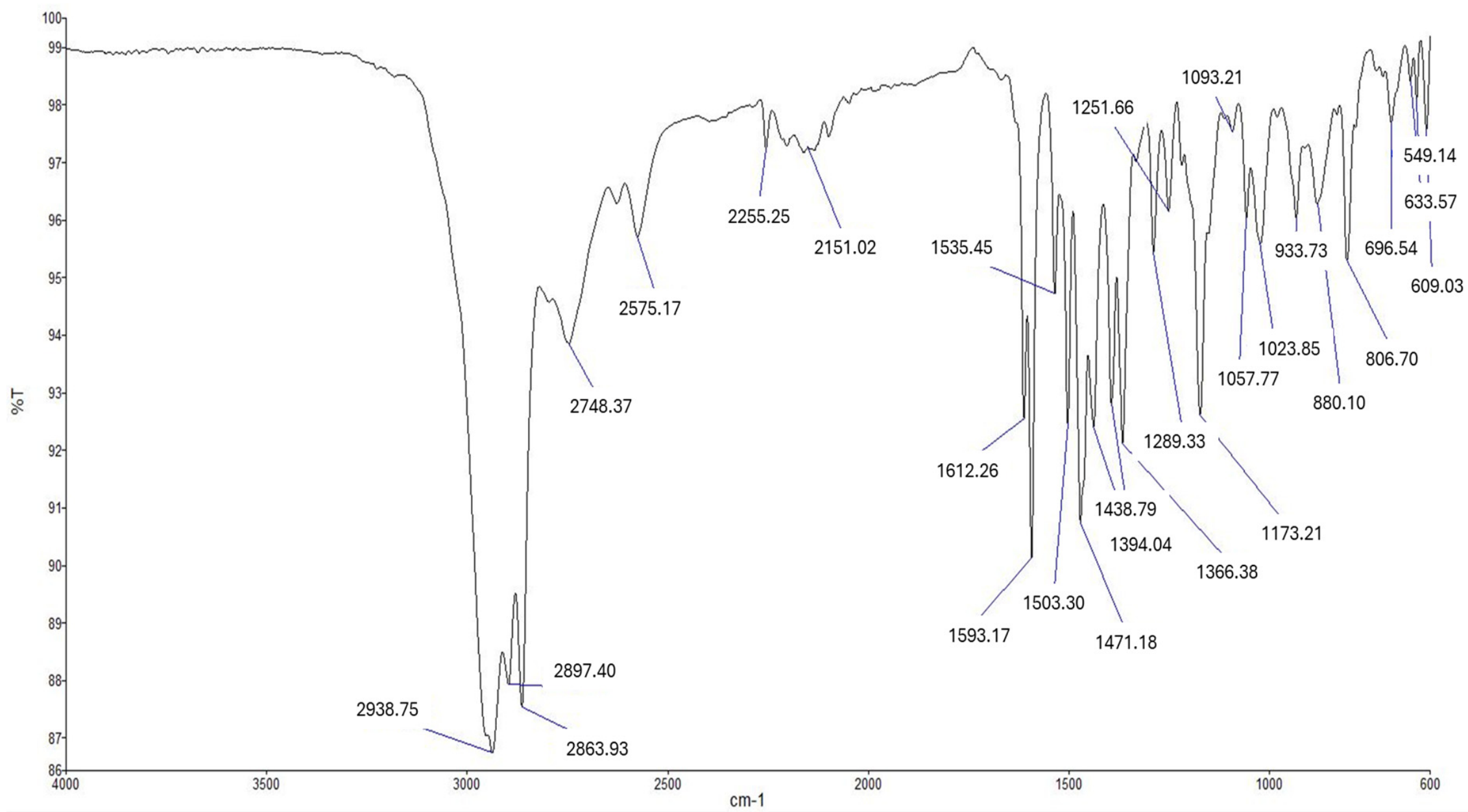

Figure S58. ATR-IR spectrum of **4-D^{PNNP}** measured as a film under N_2 flow at 298 K.

S3.4. [PNNP$^{MeCN\text{-}d4}$-RuCl(*p*-cymene)] 2Cl (4-D^{MeCN})

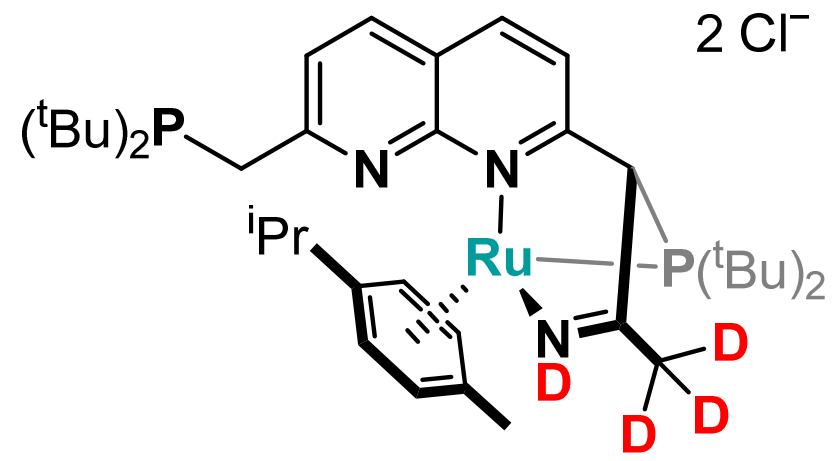

A 20 mL glass vial was charged with **2** (37.6 mg, 0.05 mmol), followed by addition of CD_3CN (7.0 mL) to give an orange solution. Next, a catalytic amount of Et_3N was added (10%, 1.0 mL of a 5 mM MeCN solution). The reaction mixture was kept stirring in a glovebox at RT for 2 h, and the color of the reaction mixture became red with a substantial amount of yellow precipitate formed. The mixture was filtered through a small glass frit to give a yellow residue. It was washed with THF (5.0 mL) and dried under dynamic vacuum to give the product as a yellow powder (38.3 mg, 96%).

^{1}H NMR (400 MHz, CD_3CN, 298 K): 14.42 (s, 0.1H*, broad), 8.65 (d, $^3J_{H,H}$ = 8.1 Hz, 1H), 8.45 (d, $^3J_{H,H}$ = 8.4 Hz, 1H), 8.28 (d, $^3J_{H,H}$ = 8.4 Hz, 1H), 8.26 – 8.24 (m, 1H, broad), 7.92 (d, $^3J_{H,H}$ = 8.3 Hz, 1H), 6.87 (d, $^3J_{H,H}$ = 6.3 Hz, 1H), 6.68 (d, $^3J_{H,H}$ = 7.5 Hz, 1H), 6.25 (d, $^1J_{H,P}$ = 8.5 Hz, 1H), 5.78 (d, $^3J_{H,H}$ = 4.9 Hz, 1H, broad), 4.21 (hept, $^3J_{H,H}$ = 6.7 Hz, 1H), 3.60 (d, $^1J_{H,P}$ = 2.5 Hz, 1H), 2.71 – 2.65 (m, 0.2H*, broad), 2.45 (s, 1H), 1.49 (d, $^3J_{H,P}$ = 13.5 Hz, 9H), 1.33 (d, $^3J_{H,H}$ = 6.9 Hz, 3H), 1.20 (d, $^3J_{H,P}$ = 11.1 Hz, 9H), 1.16 (d, $^3J_{H,P}$ = 11.1 Hz, 9H), 0.92 (d, $^3J_{H,H}$ = 6.8 Hz, 3H), 0.91 (d, $^3J_{H,P}$ = 13.5 Hz, 9H).

^{2}H NMR (61 MHz, CD_3CN, 298 K): δ = 14.4 (s, broad, 1D), 2.7 (s, broad, 3D).

**Note: the NH and the methyl group of the activated MeCN fragment are deuterated.*

The deuteration percentages: 88% D at the NH of the ketimine and 92% D at the CH_3 group of the ketimine fragment.

ATR-IR (film, N_2 flow, 298 K): ν = 2937 (m), 2895 (m), 2865 (m), 2769 (w), 2255 (w), 2206 (w), 2149 (w), 2099 (w), 2049 (w), 1612 (m), 1593 (m), 1538 (m), 1504 (m), 1470 (m), 1438 (m), 1396 (m), 1366 (m), 1289 (w), 1173 (m), 1056 (w), 1024 (w), 933 (w), 885 (w), 807 (w), 694 (w), 636 (w) cm^{-1}.

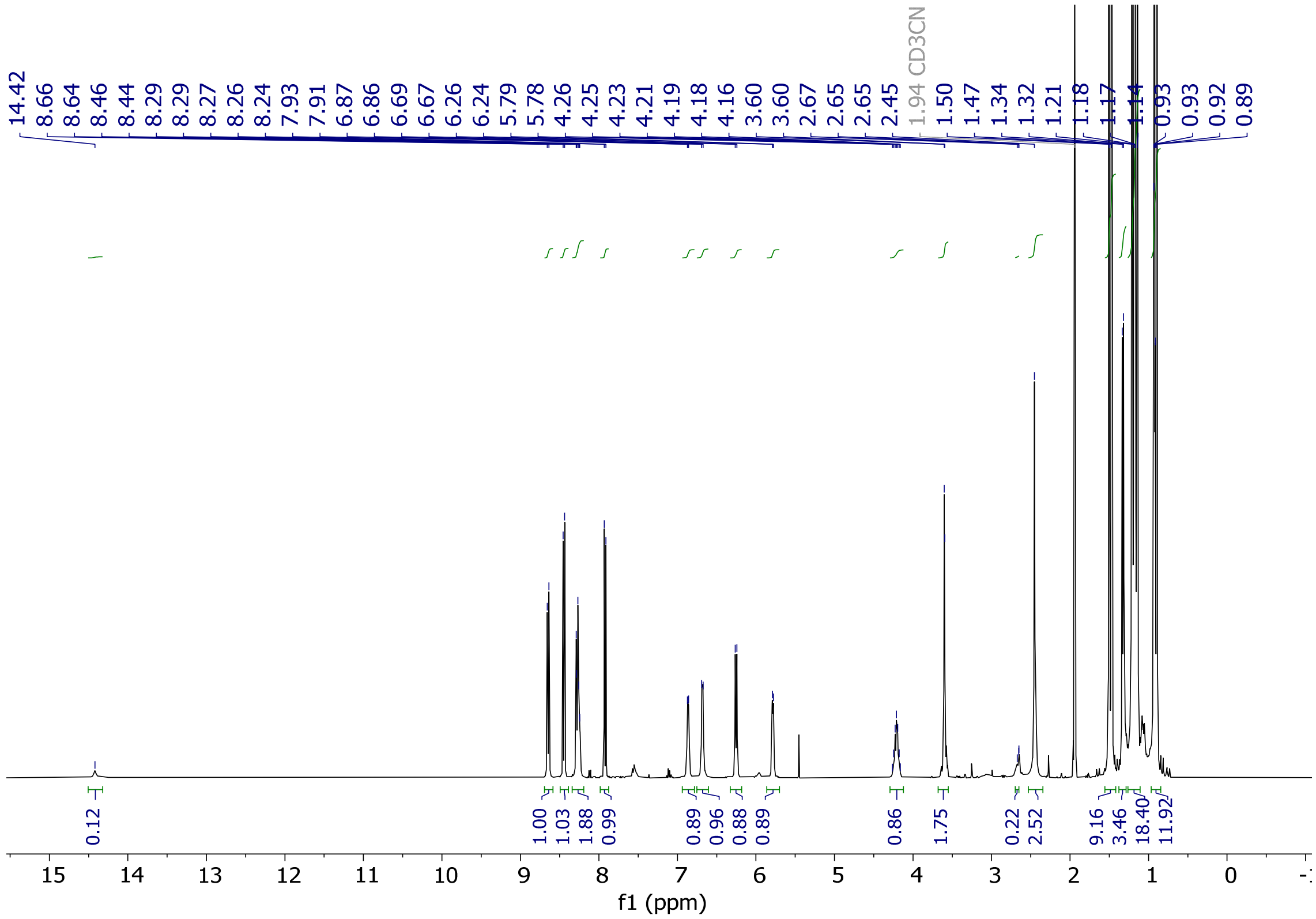

Figure S59. ^{1}H NMR spectrum of **4-D^{MeCN}** in CD_3CN at 298 K.

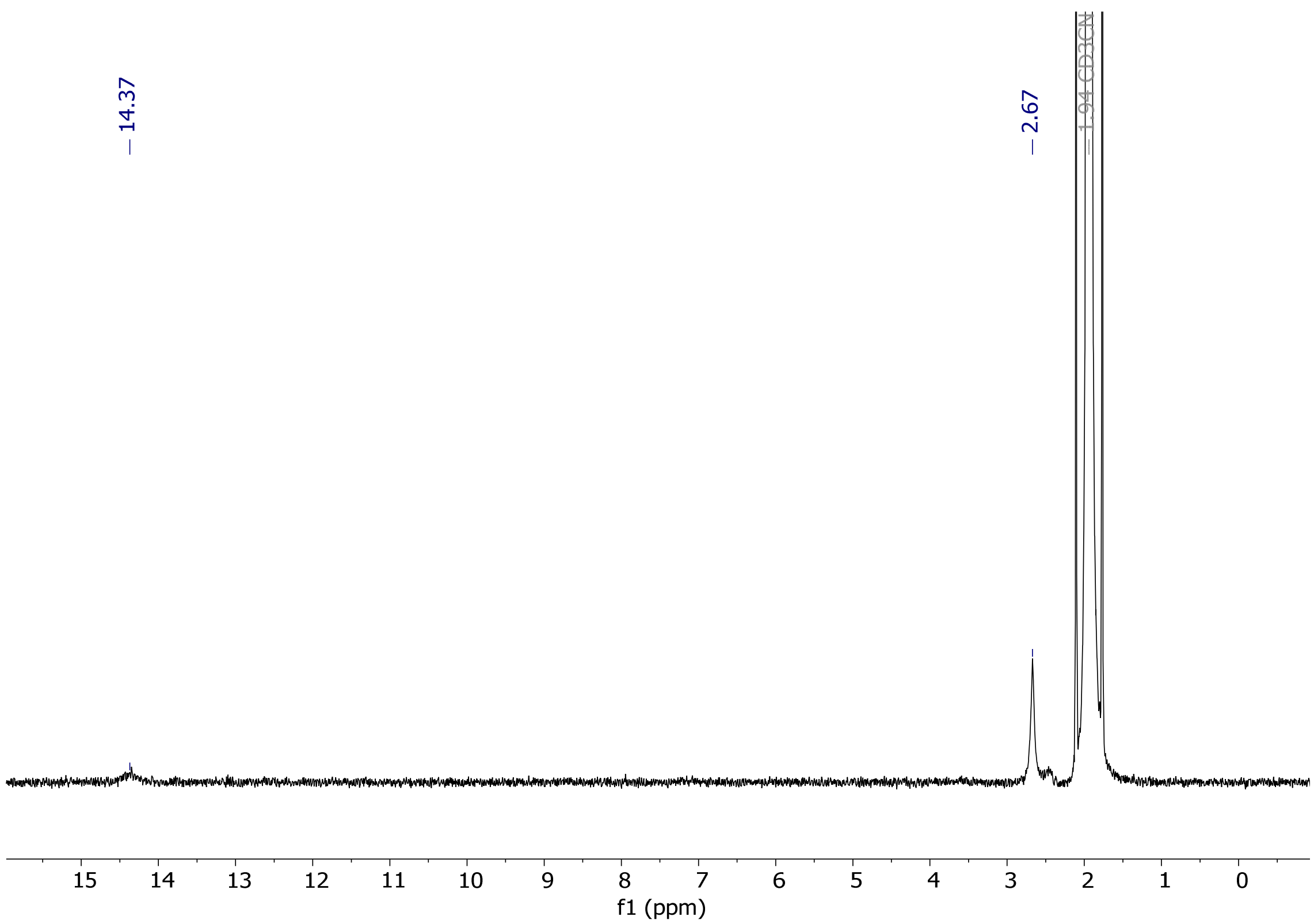

Figure S60. ^{2}H NMR spectrum of **4-D^{MeCN}** in CD_3CN at 298 K.

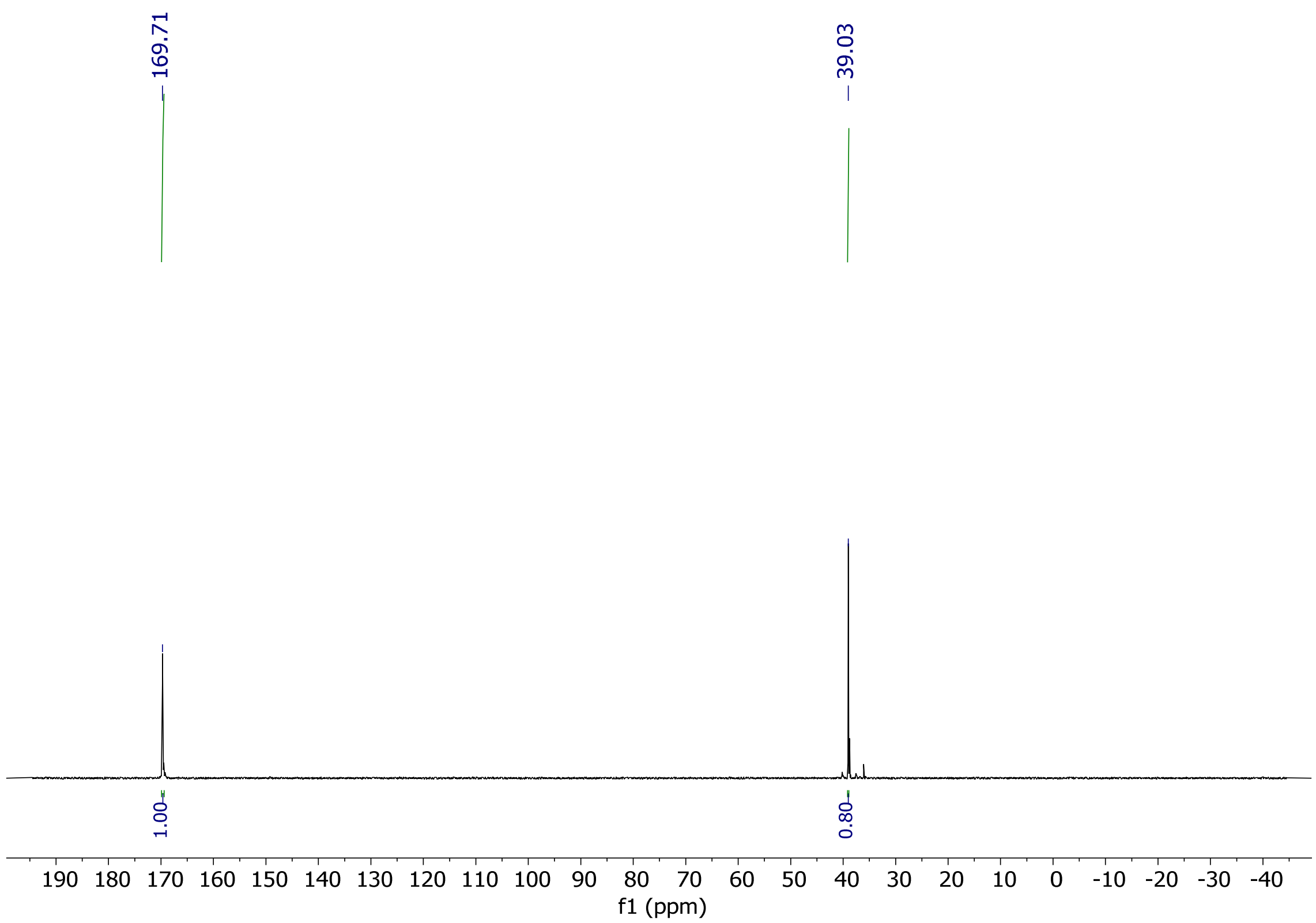

Figure S61. $^{31}P\{^{1}H\}$ NMR spectrum of **4-D^{MeCN}** in CD_3CN at 298 K.

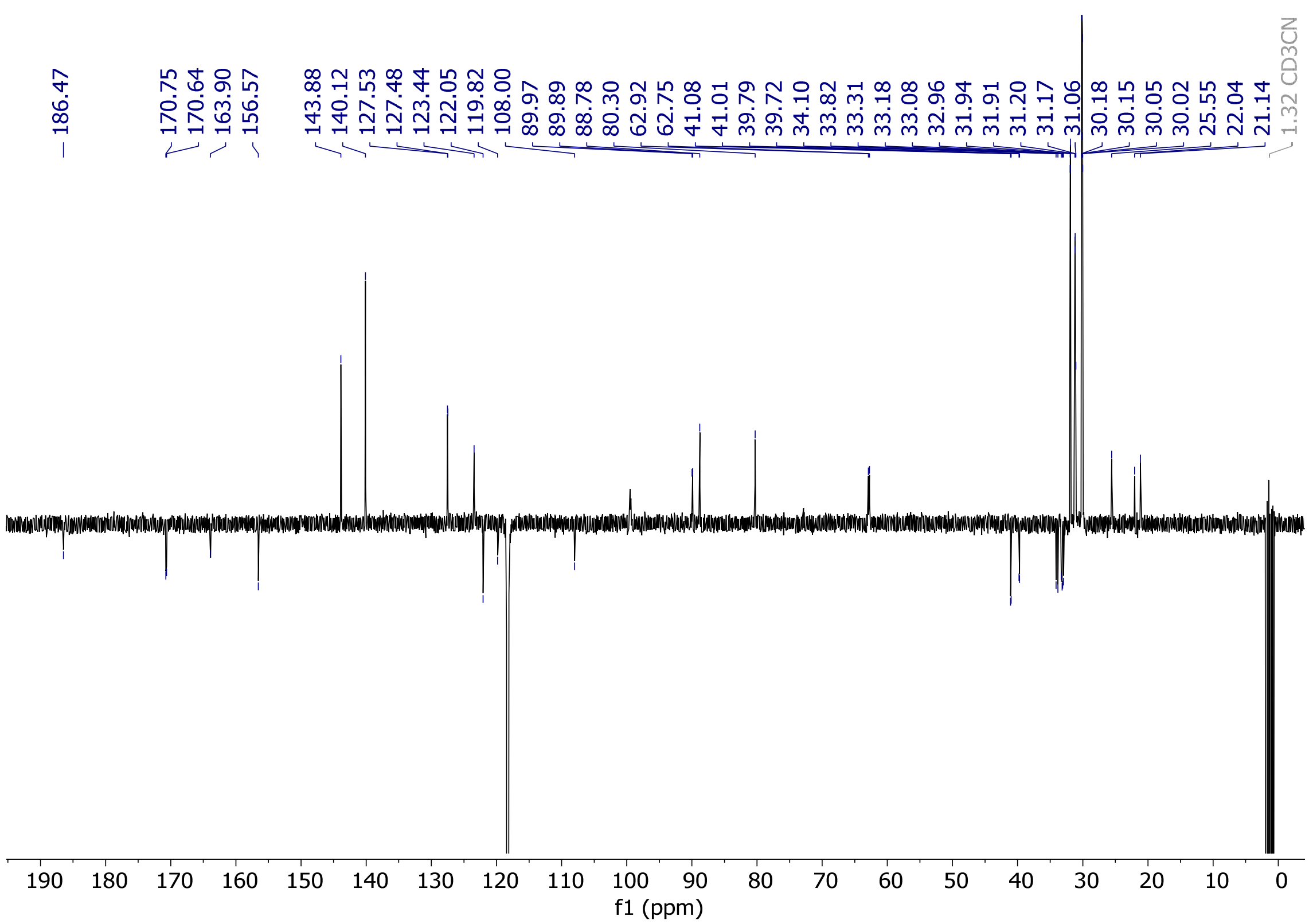

Figure S62. $^{13}C\{^{1}H\}$ APT NMR spectrum of **4-D^{MeCN}** in CD_3CN at 298 K.

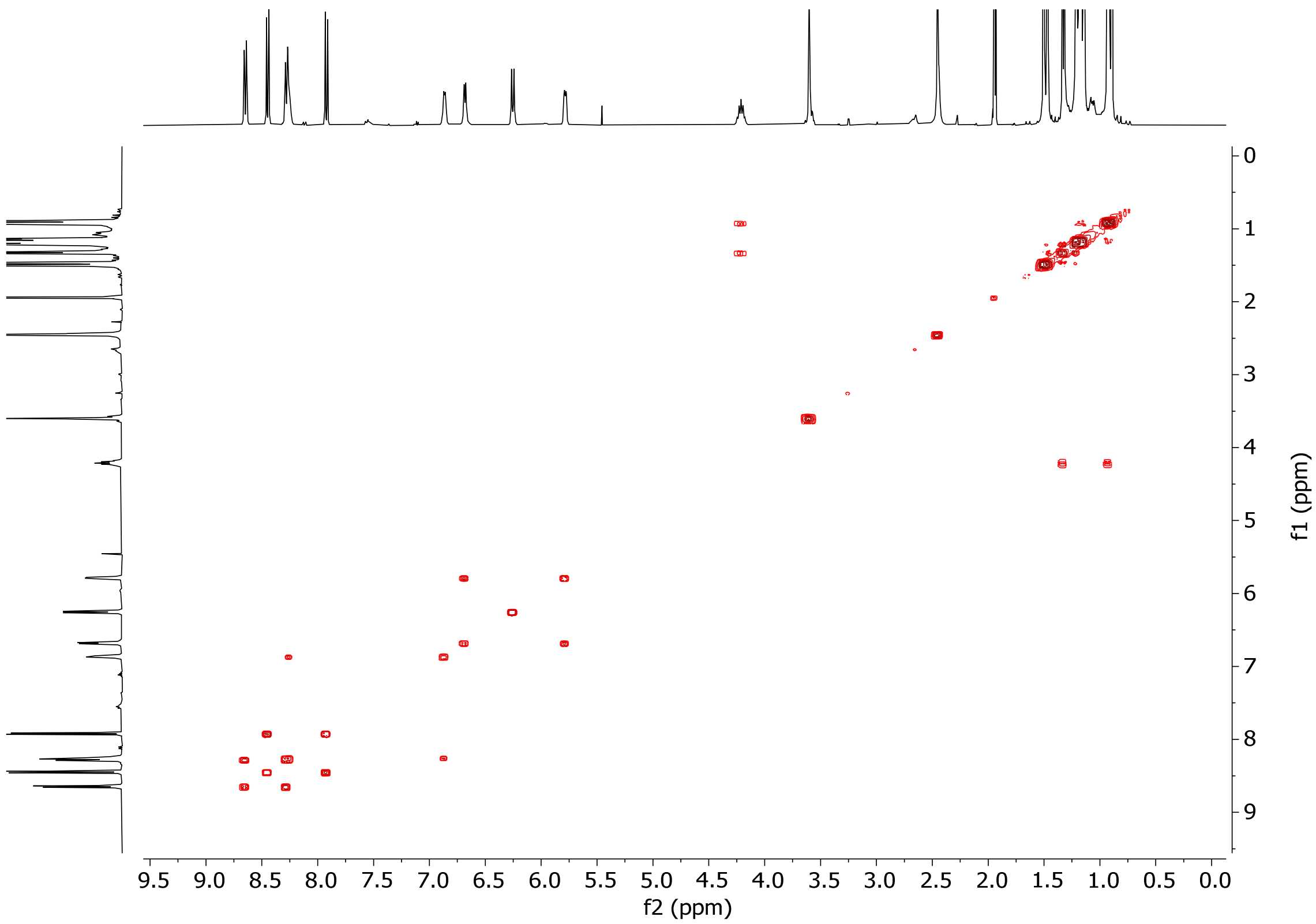

Figure S63. ^{1}H-^{1}H COSY NMR spectrum of **4-D^{MeCN}** in CD_3CN at 298 K.

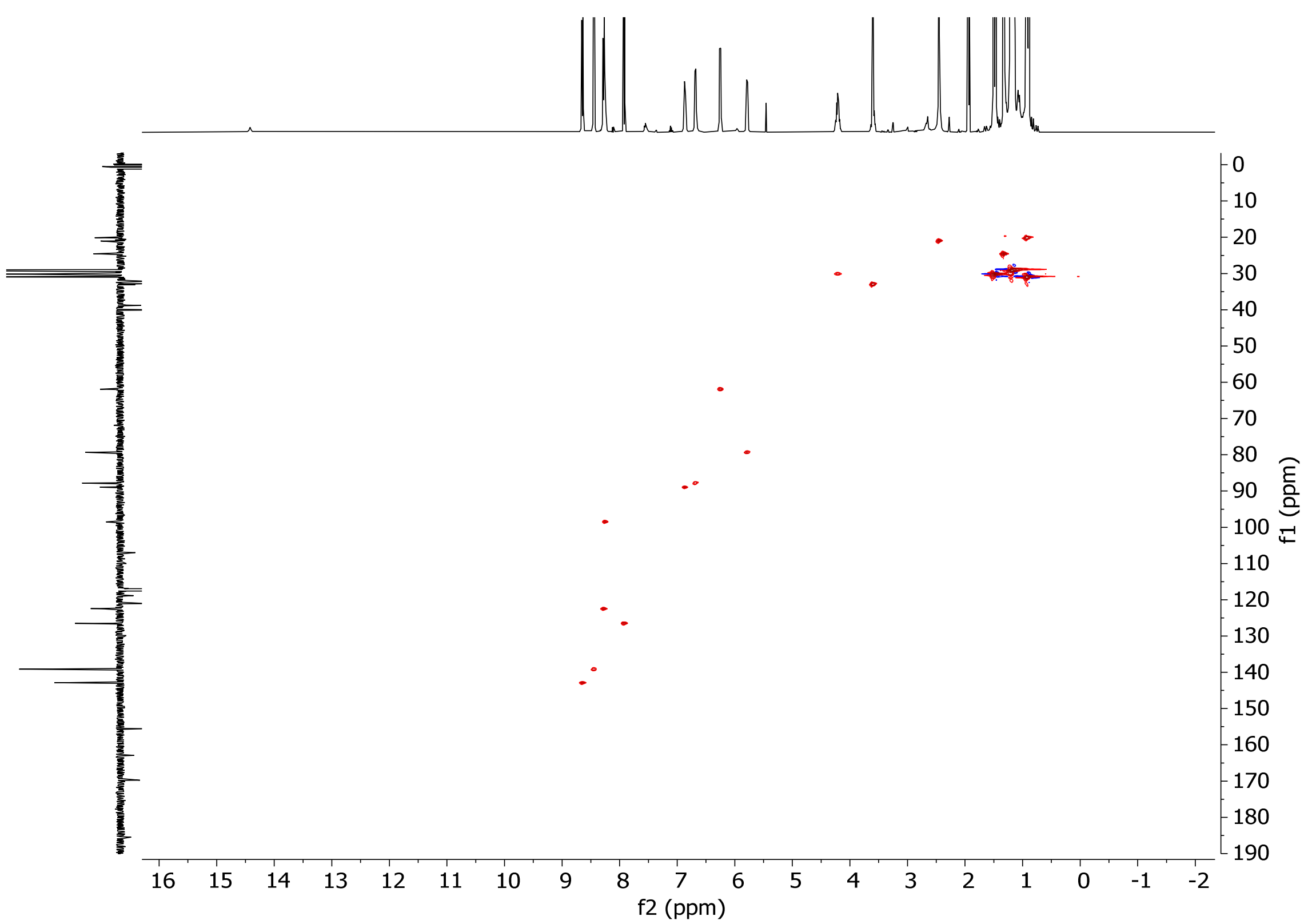

Figure S64. ^{1}H-^{13}C ASAP-HMQC NMR spectrum of **4-D^{MeCN}** in CD_3CN at 298 K.

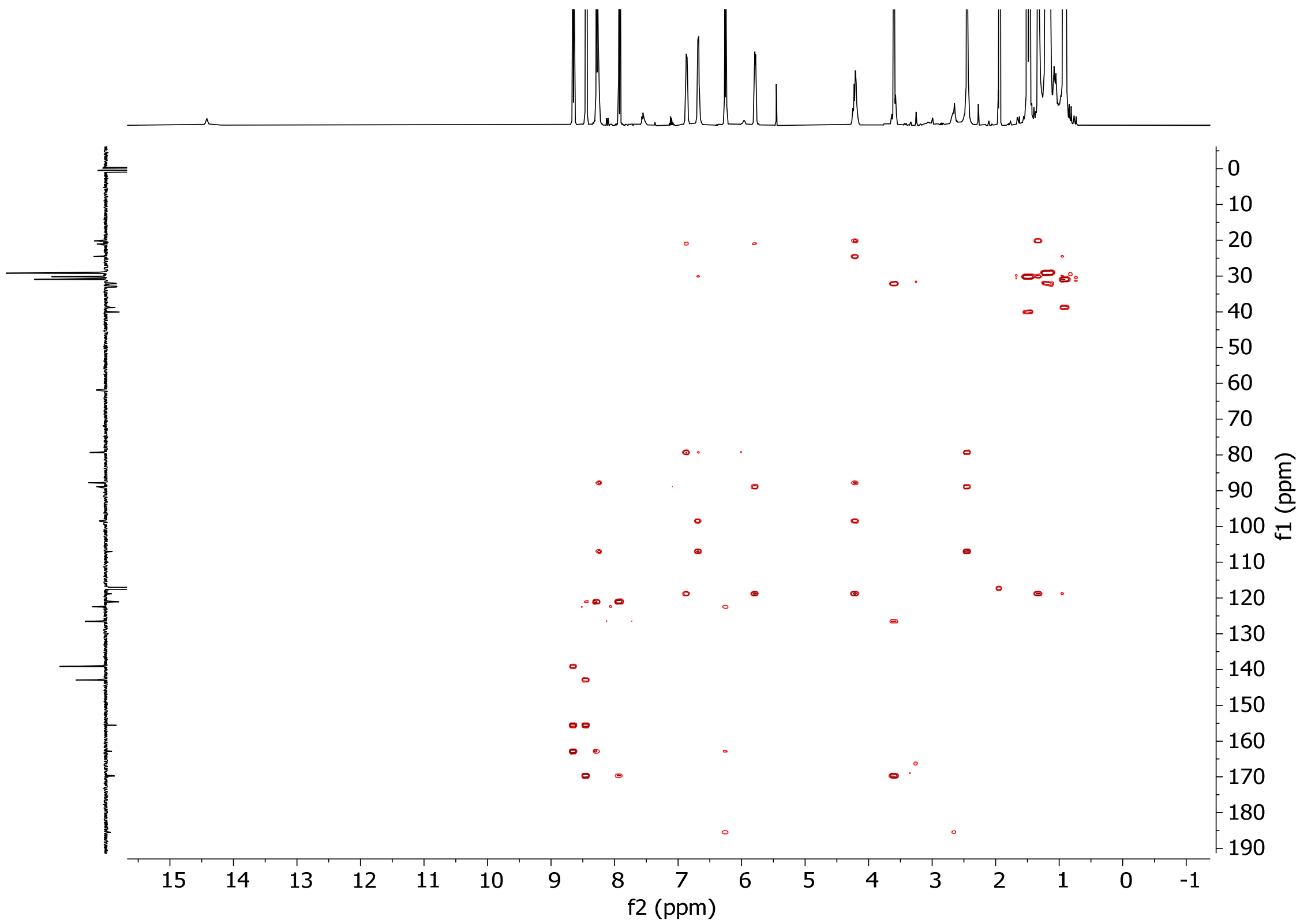

Figure S65. ^{1}H-^{13}C HMBC NMR spectrum of **4-D$^{\mathbf{MeCN}}$** in CD_3CN at 298 K.

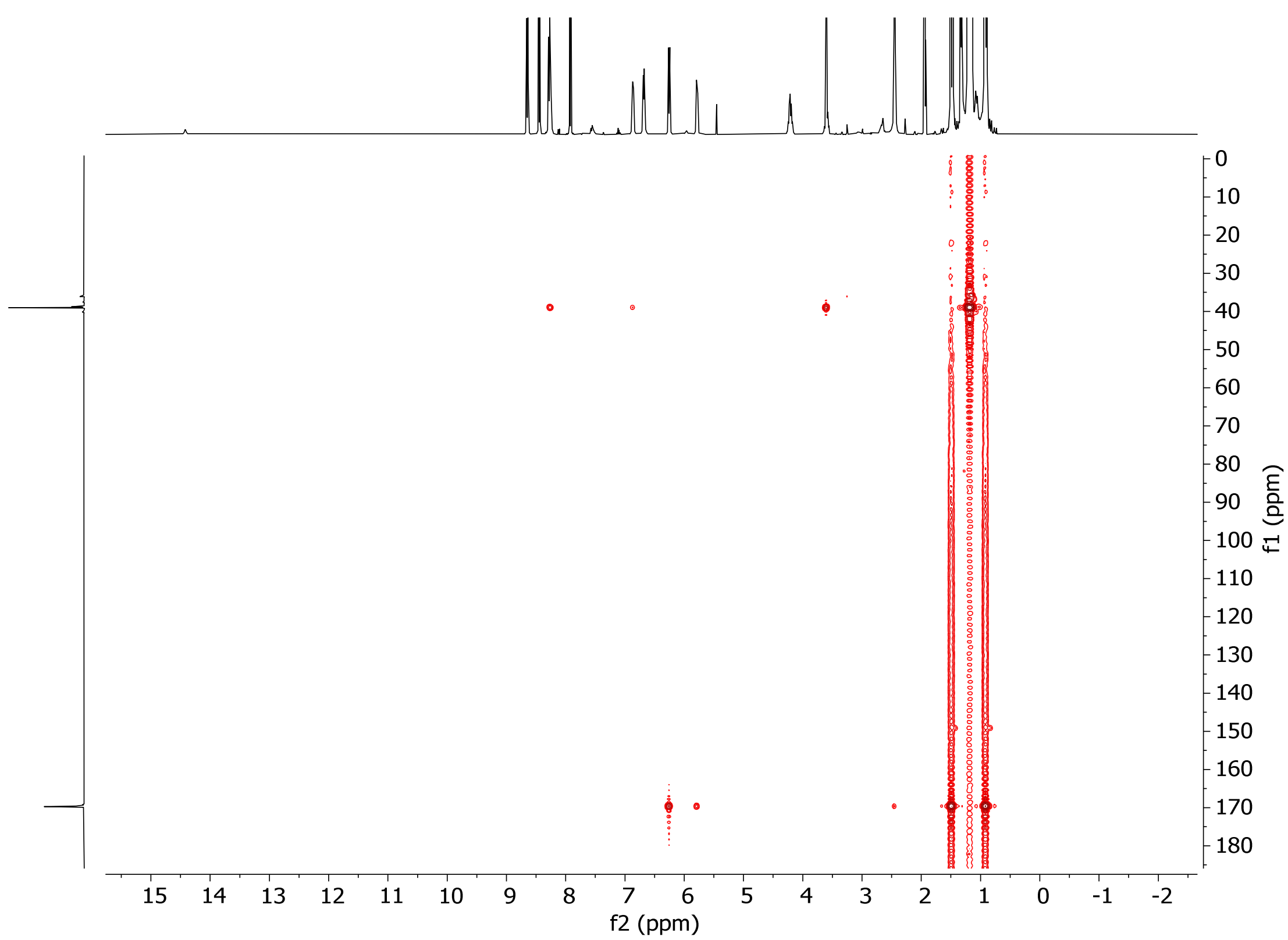

Figure S66. ^{1}H-^{31}P HMBC NMR spectrum of **4-D$^{\mathbf{MeCN}}$** in CD_3CN at 298 K.

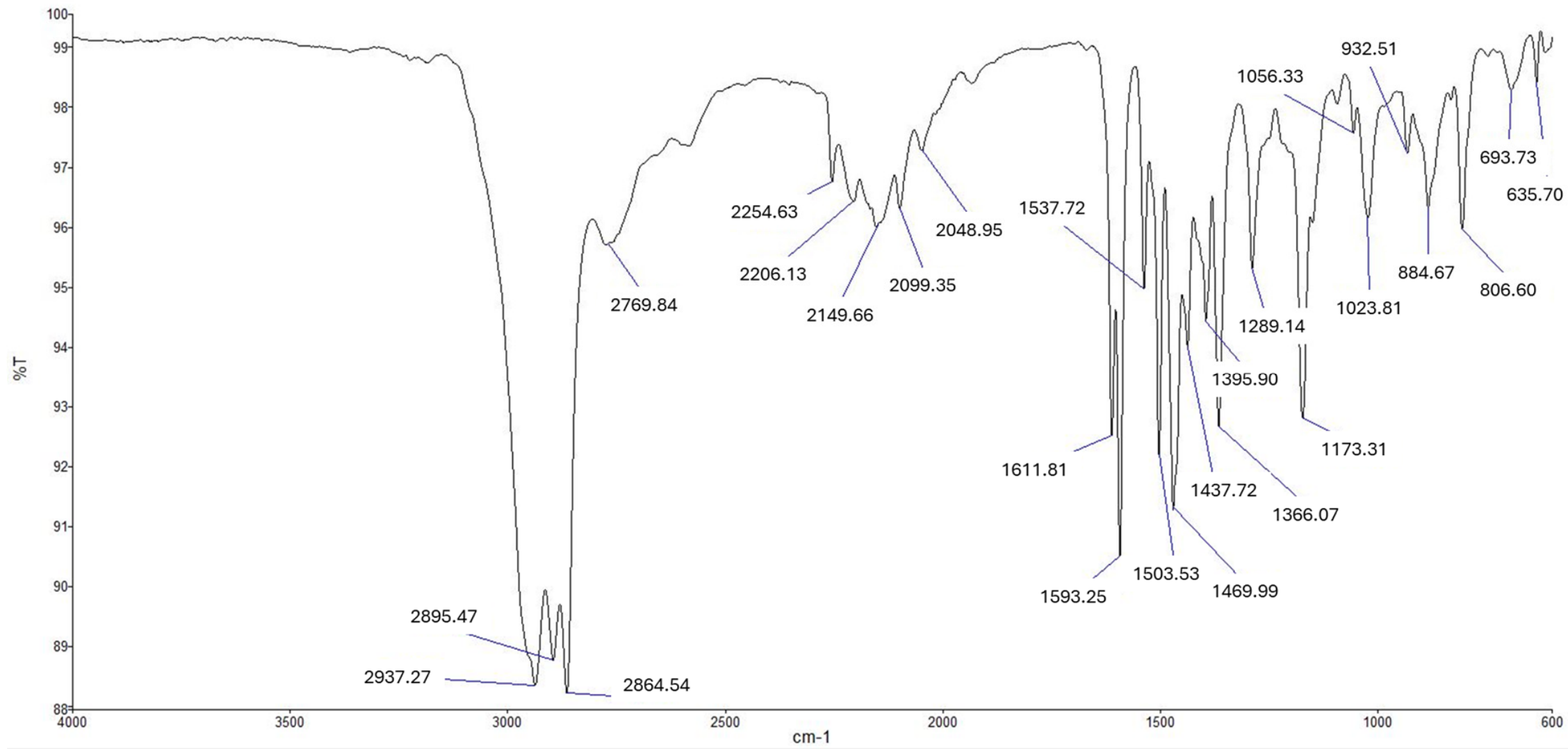

Figure S67. ATR-IR spectrum of **4-D^{MeCN}** measured as a film under N_2 flow at 298 K.

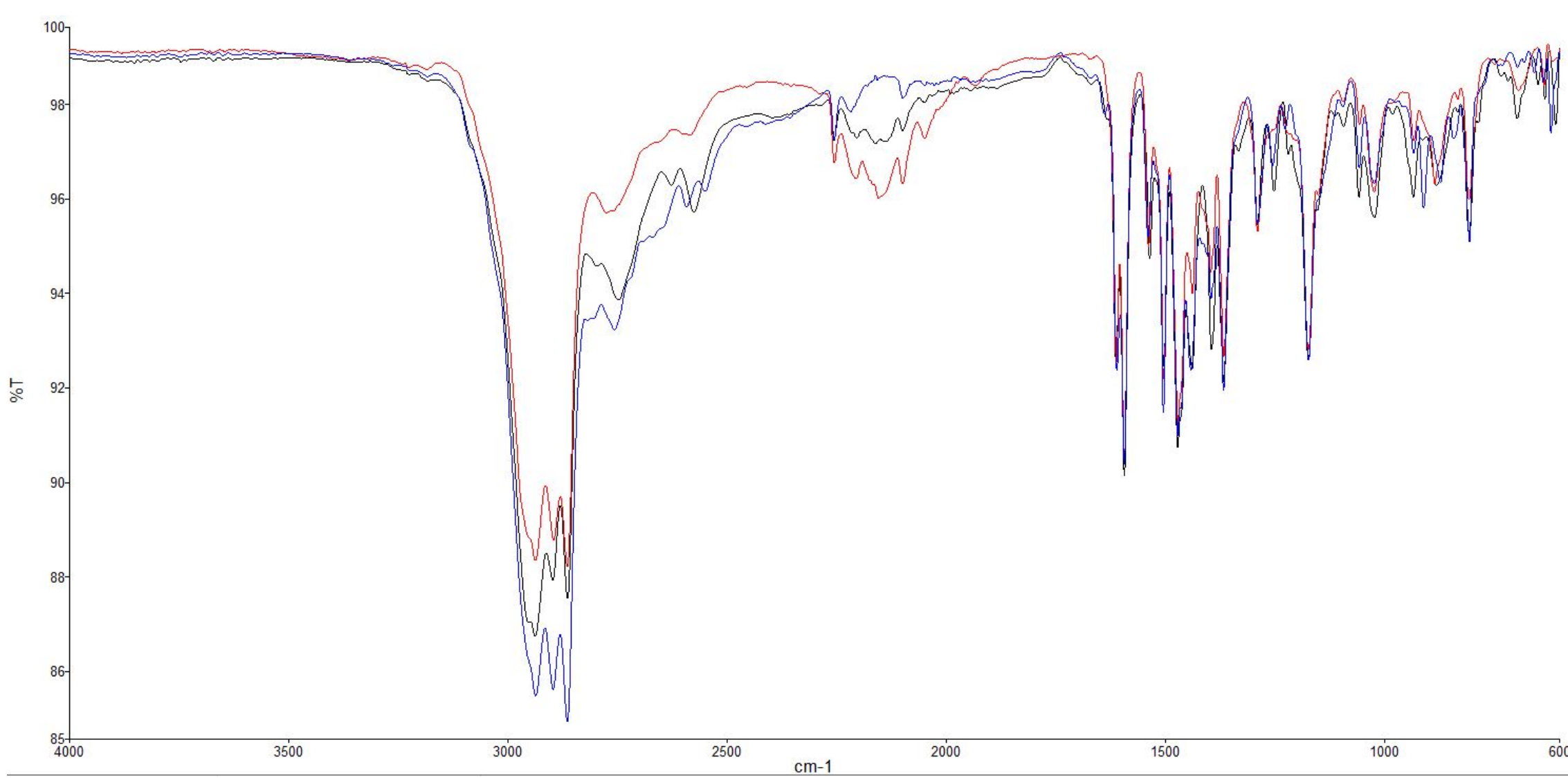

Figure S68. Overlapped ATR-IR spectra of **4** (blue), **4-D^{PNNP}** (black) and **4-D^{MeCN}** (red) measured as a film under N_2 flow at 298 K.

S4. Computational Part

All calculations of molecules and complexes were carried out using DFT[4] as implemented in ORCA 6.1.[5–8] Both geometry optimization and single point calculations were performed at BP86 level of theory. Geometry optimizations and analytical vibrational frequency calculations were carried out with the def2-SVP basis set[9] with def2-ECP for Ru.[10] For all optimized structures, the intermediates were confirmed with no imaginary vibrational frequency, while transition states showed a single imaginary frequency with a motion corresponding to the proper transitions. The solvated energies of optimized structures were re-evaluated by additional single-point calculations on each optimized geometry using the def2-TZVPP basis set.[9] For all calculations, the RIJCOSX approximation[11,12] was utilized with the auxiliary basis set def2/J.[13] To model the solution environment for MeCN, the solvation model based on density (SMD)[14] was utilized with parameters which have been implemented in ORCA. Visualization has been done using ChemCraft and CYLview20. The pK_a values were estimated according to our previously reported approach using DBU in an isodesmic reaction.[15] 1,8-Diazabicyclo[5.4.0]undec-7-ene (DBU) was selected as the reference base, as its experimental acidity has been extensively characterized in a variety of solvents, and this protocol has previously been shown to provide reliable results for closely related systems.[15]

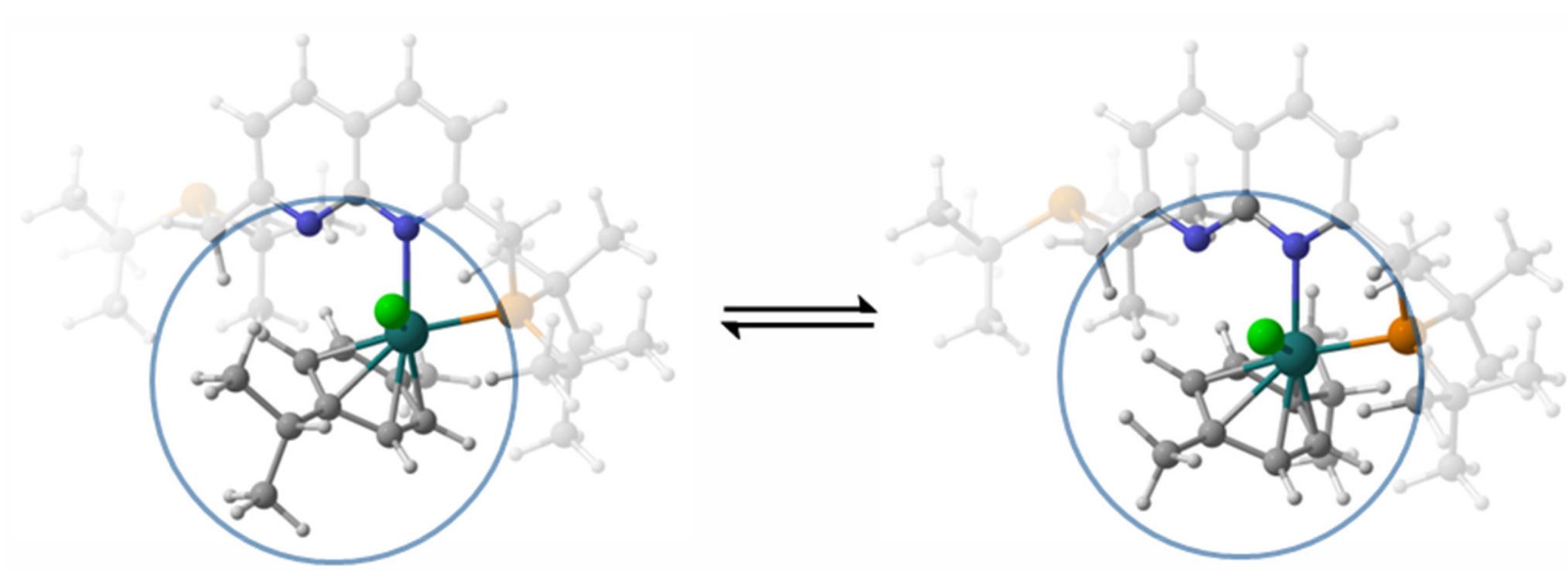

Figure S69. The rotation of the p-cymene fragment observed in solution according to the ^1H NMR for complex **2**. The geometries of different conformers of complex **2** were optimized at the BP86-D3BJ/def2-SVP level of theory.

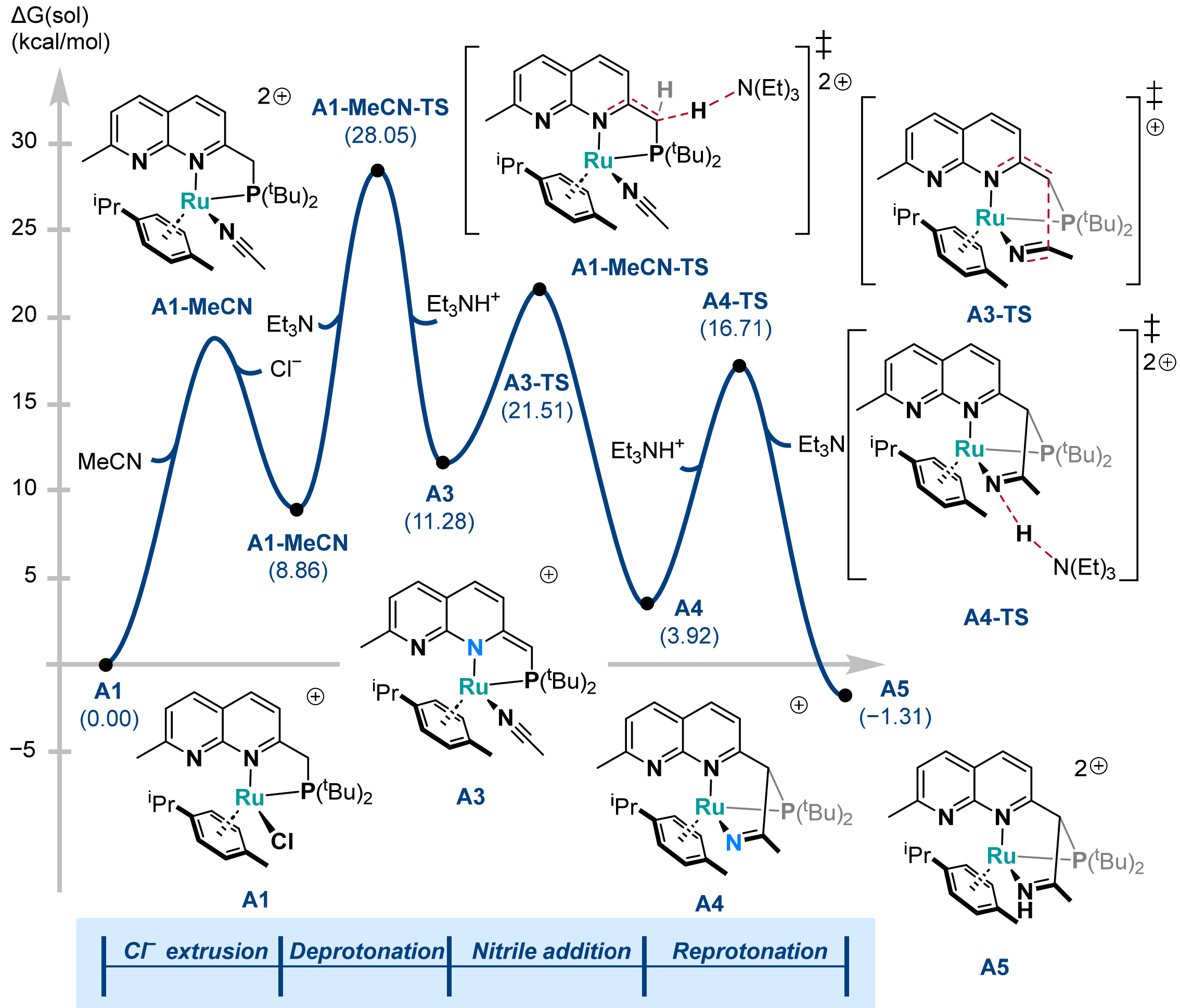

Figure S70. Computed reaction profile where the chloride extrusion precedes the deprotonation step.

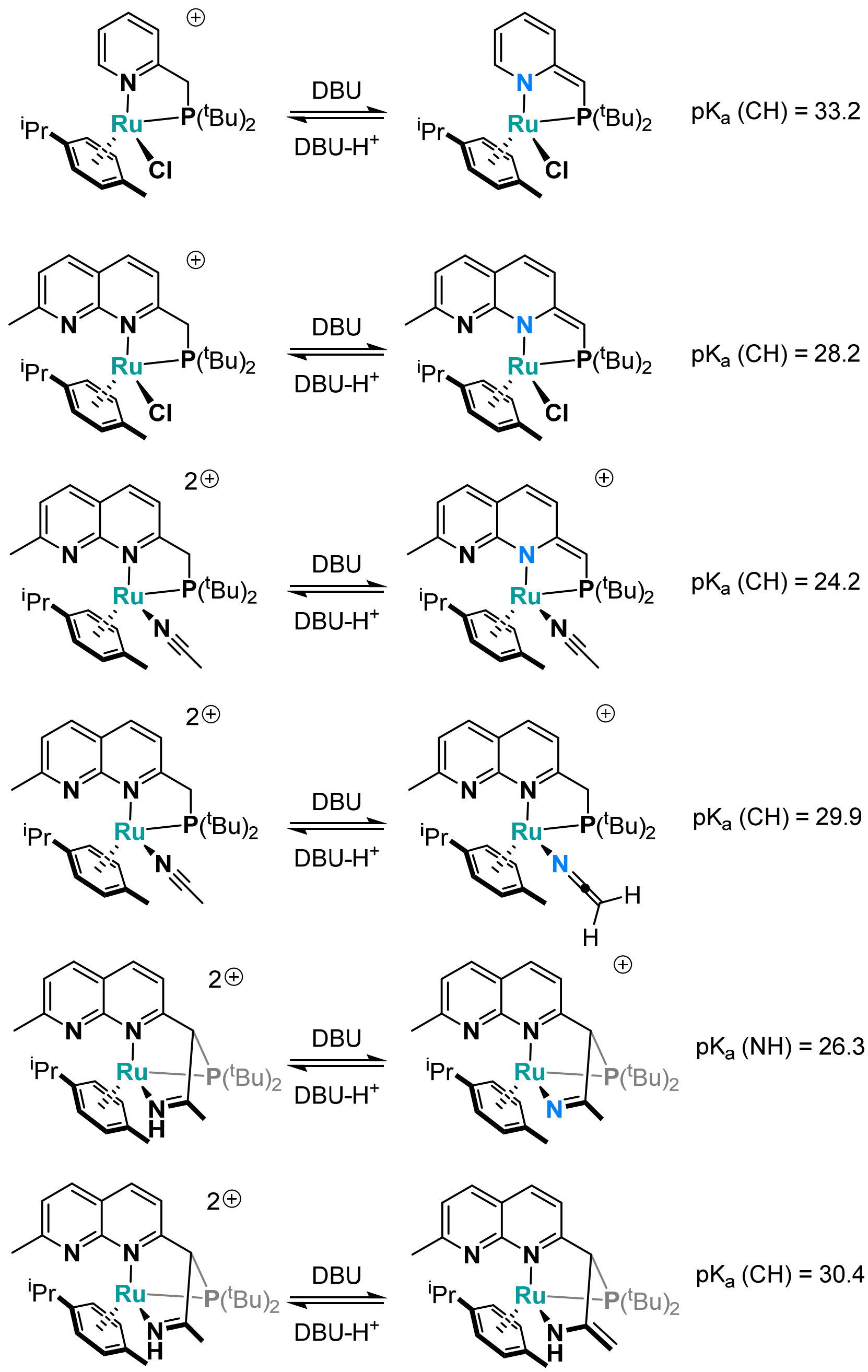

Figure S71. Computed pK_a values of various compounds participating in the MeCN activation via MLC. The computed pK_a values are based on the isodesmic approach using DBU[15,16] as an external base with the reported pK_a value DBU as 24.34 in MeCN at RT.[17] The acidity estimations were computed using formula 1. Computed at the BP86-D3BJ/def2-TZVPP//def2-SVP level with SMD (MeCN) implicit solvation at 298.15 K.

$$pK_a = \frac{\Delta G^{\circ}_{sol}}{RTln(10)} - pK_a(DBU) \quad (1)$$

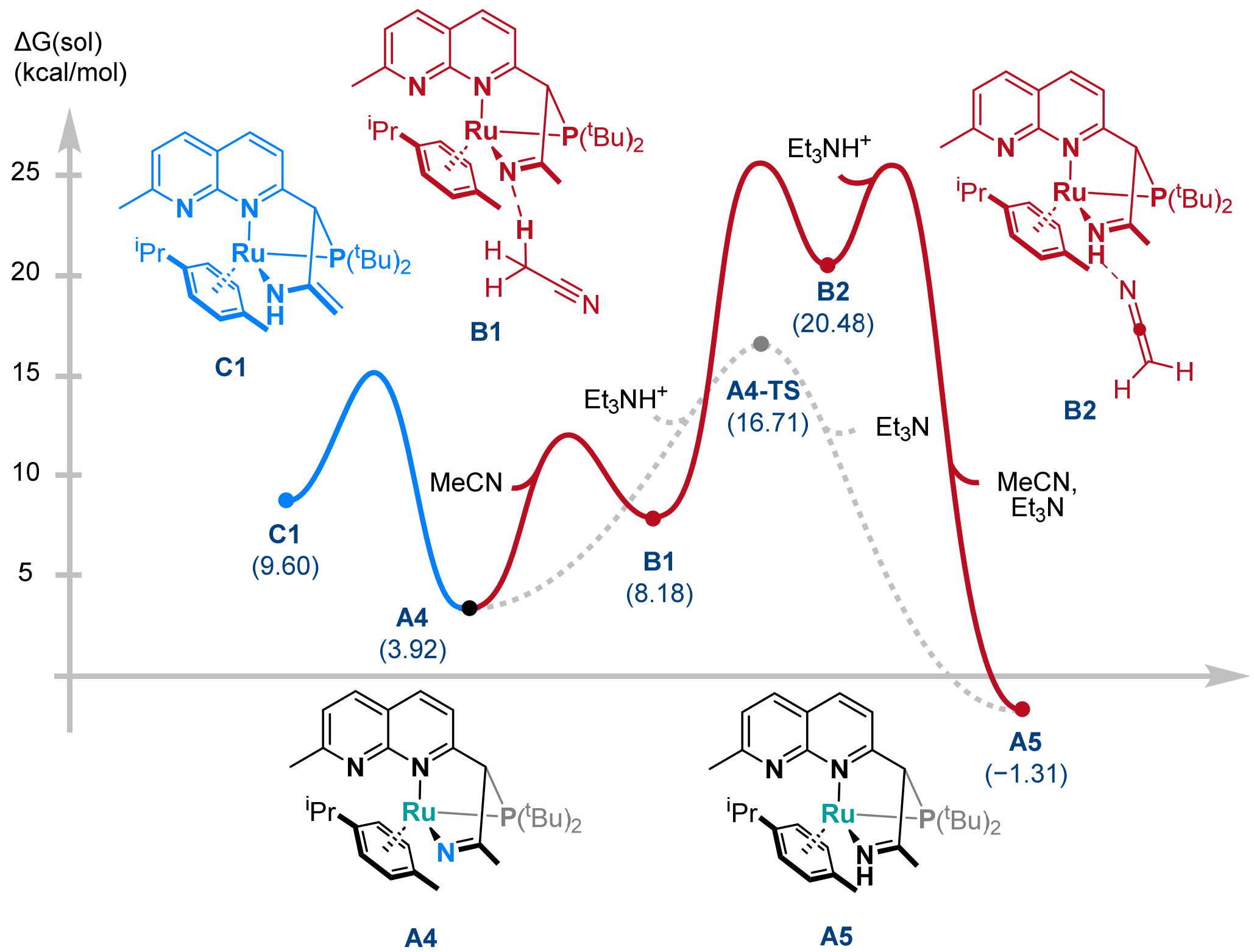

Figure S72. Computed reaction profile explaining the possible proton exchange between the MeCN solvent molecules and the activated NH fragment. Inspired by the hypothesis of Milstein and co-workers.[18]

Consistent with hypothesis suggested by Milstein and co-workers,[18] our calculations indicate that intermediate **A4** can form an associated complex with MeCN (**B1**, +8.2 kcal mol^{-1}, Figure S72). Within this associated species, direct proton transfer from the bound acetonitrile to the basic ketimido nitrogen becomes feasible, yielding the observed ketimine product and a deprotonated CH_2=C=N fragment bound via a hydrogen bonding N–H…N (**B2**, 20.5 kcal mol^{-1}). The resulting anionic fragment can subsequently be reprotonated by Et_3NH^+, thereby closing the proton-transfer cycle.

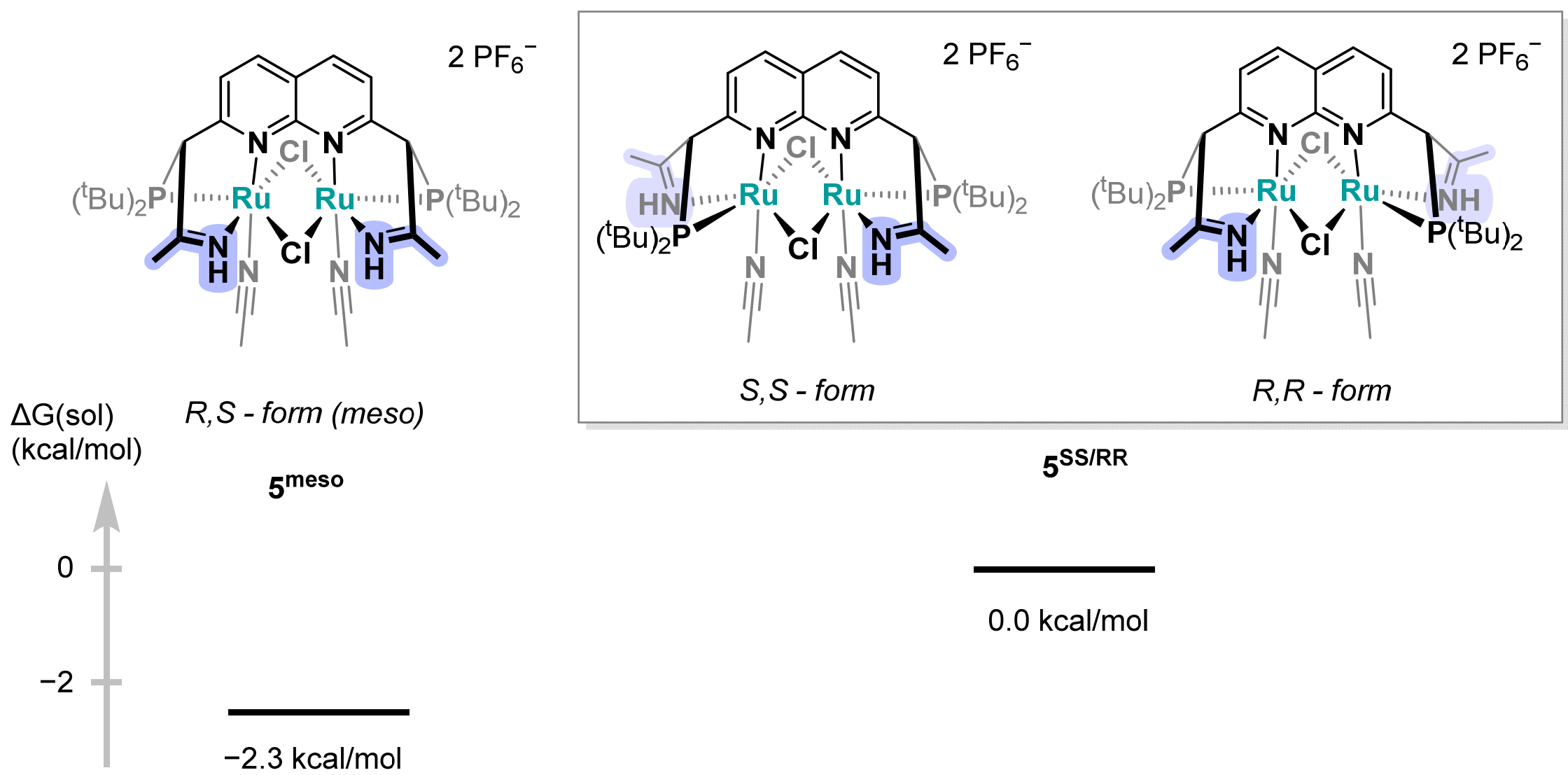

Figure S73. Relative difference in ΔG° between the diastereomers of complex **5**. Computed at the BP86-D3BJ/def2-TZVPP//def2-SVP level with SMD (MeCN) implicit solvation at 298.15 K.

S5. X-ray crystal Structures

5.1 X-ray crystal structure determination of 1-PF_6

$[C_{32}H_{50}ClN_2P_2Ru](PF_6) \cdot 0.5(CH_2Cl_2)$, Fw = 848.63, black block, 0.35 × 0.32 × 0.24 mm^3, triclinic, P $\overline{1}$ (no. 2), a = 14.6741(7), b = 14.8554(6), c = 17.5930(10) Å, α = 110.480(2), β = 98.390(2), γ = 90.815(2) °, V = 3714.4(3) Å^3, Z = 4, D_x = 1.518 g/cm^3, μ = 0.75 mm^{-1}. The diffraction experiment was performed on a Bruker Kappa ApexII diffractometer with sealed tube and Triumph monochromator (λ = 0.71073 Å) at a temperature of 150(2) K up to a resolution of $(\sin \theta/\lambda)_{max}$ = 0.65 Å^{-1}. The crystal was fragmented into several pieces. Four orientation matrices were used for the integration of the major components with the Eval15 software[19] resulting in a HKLF-5 file[20]. A multi-scan absorption correction and scaling was performed with TWINABS[21] (correction range 0.51-0.75). A total of 223369 reflections was measured, 17966 reflections were unique (R_{int} = 0.083), 13980 reflections were observed [I>2σ(I)]. The structure was solved with Patterson superposition methods using SHELXT.[22] Structure refinement was performed with SHELXL-2018[23] on F^2 of all reflections. Non-hydrogen atoms were refined freely with anisotropic displacement parameters. One of the PF_6 anions was refined with a disorder model. Hydrogen atoms were introduced in calculated positions and refined with a riding model. 937 Parameters were refined with 1547 restraints (geometry and displacement parameters of the PF_6 anions and the CH_2Cl_2 solvent). R1/wR2 [I > 2σ(I)]: 0.0723 / 0.1964. R1/wR2 [all refl.]: 0.0935 / 0.2076. S = 1.089. BASF values for the additional crystal fragments: 0.265(4), 0.0971(12), 0.072(2). Residual electron density between -1.14 and 4.66 e/Å^3. Geometry calculations and checking for higher symmetry was performed with the PLATON program.[24]

Table S1. *Key geometric parameters of complex **1-PF₆** derived from the X-ray crystal structure. There are two independent complex molecules in the asymmetric unit, X=1,2.*

Atoms	2, Å	Atoms	2, Å/°
Ru(X)-(C_6H_6)(X)^{Centr}	1.719(3)/1.722(3)	C(5X)-C(7X)	1.394(10)/1.416(10)
Ru(X)-Cl(X)	2.3978(17)/2.3989(18)	C(7X)-C(8X)	1.369(10)/1.342(11)
Ru(X)-N(1X)	2.183(5)/2.199(5)	C(8X)-C(9X)	1.415(9)/1.420(10)
Ru(X)-P(1X)	2.3559(18)/2.3456(18)	C(9X)-N(2X)	1.323(9)/1.342(9)
Ru(X)-C(27X)	2.229(7)/2.225(7)	N(2X)-C(6X)	1.359(9)/1.340(9)
Ru(X)-C(28X)	2.210(7)/2.206(7)	C(9X)-C(10X)	1.490(9)/1.502(10)
Ru(X)-C(29X)	2.254(7)/2.277(7)	C(10X)-P(2X)	1.881(7)/1.875(7)
Ru(X)-C(30X)	2.252(6)/2.252(7)	P(1X)-Ru(X)-Cl(X)	89.55(6)/90.38(6)
Ru(X)-C(31X)	2.178(6)/2.187(7)	P(1X)-Ru(X)-N(1X)	78.28(15)/80.09(16)
Ru(X)-C(32X)	2.187(7)/2.195(7)	P(1X)-Ru(X)-(C_6H_6)(X)^{Centr}	132.02(11)/132.12(12)
P(1X)-C(1X)	1.825(7)/1.813(7)	N(1X)-Ru(X)-(C_6H_6)(X)^{Centr}	135.07(19)/135.87(19)
C(1X)-C(2X)	1.492(10)/1.501(9)	Ru(X)-(C_6H_6)(X)^{Centr}-C(27X)	90.3(3)/89.9(4)
C(2X)-C(3X)	1.414(9)/1.410(9)	Ru(X)-(C_6H_6)(X)^{Centr}-C(28X)	89.5(3)/89.4(3)
C(3X)-C(4X)	1.362(10)/1.355(10)	Ru(X)-(C_6H_6)(X)^{Centr}-C(29X)	91.9(3)/92.6(3)
C(4X)-C(5X)	1.410(10)/1.407(10)	Ru(X)-(C_6H_6)(X)^{Centr}-C(30X)	92.0(3)/91.9(4)
C(5X)-C(6X)	1.417(9)/1.416(9)	Ru(X)-(C_6H_6)(X)^{Centr}-C(31X)	87.9(3)/87.9(4)
C(6X)-N(1X)	1.381(9)/1.407(8)	Ru(X)-(C_6H_6)(X)^{Centr}-C(32X)	88.3(3)/88.3(4)
N(1X)-C(2X)	1.355(9)/1.334(9)		

5.2 X-ray crystal structure determination of 4

$[C_{38}H_{61}N_3P_2Ru]Cl_2 \cdot 1.5(C_2H_3N)$, Fw = 855.39, yellow plate, 0.43 × 0.20 × 0.04 mm^3, monoclinic, $P2_1/n$ (no. 14), a = 9.5513(3), b = 30.9404(11), c = 15.6280(4) Å, β = 105.351(1)°, V = 4453.6(2) $Å^3$, Z = 4, D_x = 1.276 g/cm^3, μ = 0.58 mm^{-1}. The diffraction experiment was performed on a Bruker Kappa ApexII diffractometer with sealed tube and Triumph monochromator (λ = 0.71073 Å) at a temperature of 150(2) K up to a resolution of $(\sin \theta/\lambda)_{max}$ = 0.65 $Å^{-1}$. The Eval15 software[19] was used for the intensity integration. A numerical absorption correction and scaling was performed with SADABS[25] (correction range 0.81-0.99). A total of 85849 reflections was measured, 10239 reflections were unique (R_{int} = 0.033), 9126 reflections were observed [$I>2\sigma(I)$]. The structure was solved with Patterson superposition methods using SHELXT.[22] Structure refinement was performed with SHELXL-2018[23] on F^2 of all reflections. Non-hydrogen atoms were refined freely with anisotropic displacement parameters. Two t-butyl groups were refined with a disorder model. Minor disorder in the acetonitrile molecules was not resolved. The N-H hydrogen atom was located in difference Fourier maps and refined freely with an isotropic displacement parameter. The C-H hydrogen atoms were introduced in calculated positions and refined with a riding model. 526 Parameters were refined with 307 restraints (distances, angles of the t-butyl and acetonitrile moieties; displacement parameters of the t-butyl groups). R1/wR2 [$I > 2\sigma(I)$]: 0.0277 / 0.0685. R1/wR2 [all refl.]: 0.0323 / 0.0706. S = 1.054. Residual electron density between -0.84 and 1.20 e/$Å^3$. Geometry calculations and checking for higher symmetry was performed with the PLATON program.

CCDC 2533038-2533039 contain the supplementary crystallographic data for this paper. These data can be obtained free of charge from The Cambridge Crystallographic Data Centre via www.ccdc.cam.ac.uk/data_request/cif.

Table S2. *Key geometric parameters of complex **4** derived from the X-ray crystal structure.*

Atoms	4, Å	Atoms	4, Å/°
Ru(1)-(*p*-cym)^{Centr}	1.7340(8)	C(5)-C(7)	1.419(2)
Ru(1)-N(1)	2.1543(14)	C(7)-C(8)	1.359(3)
Ru(1)-N(3)	2.0592(14)	C(8)-C(9)	1.423(3)
Ru(1)-P(1)	2.3496(4)	C(9)-N(2)	1.327(2)
Ru(1)-C(30)	2.2680(17)	N(2)-C(6)	1.348(2)
Ru(1)-C(31)	2.2474(17)	C(9)-C(10)	1.500(2)
Ru(1)-C(32)	2.2583(17)	C(10)-P(2)	1.8795(19)
Ru(1)-C(33)	2.2611(17)	P(1)-Ru(1)-(*p*-cym)^{Centr}	137.34(4)
Ru(1)-C(34)	2.1879(17)	N(1)-Ru(1)-(*p*-cym)^{Centr}	134.62(5)
Ru(1)-C(35)	2.2065(17)	N(3)-Ru(1)-(*p*-cym)^{Centr}	125.39(6)
P(1)-C(1)	1.8795(17)	P(1)-Ru(1)-N(1)	77.28(4)
N(3)-C(11)	1.274(2)	P(1)-Ru(1)-N(3)	79.37(4)
C(1)-C(11)	1.510(2)	C(1)-C(11)-N(3)	114.33(15)
C(11)-C(12)	1.493(2)	C(1)-C(11)-C(12)	119.73(15)
C(1)-C(2)	1.506(2)	N(3)-C(11)-C(12)	125.74(16)
C(2)-C(3)	1.409(2)	C(2)-C(1)-C(11)	102.68(13)
C(3)-C(4)	1.369(3)	C(2)-C(1)-P(1)	105.23(11)
C(5)-C(6)	1.418(2)	P(1)-C(1)-C(11)	106.09(11)
C(6)-N(1)	1.386(2)	Ru(1)-(*p*-cym)^{Centr}-C(30–35)	87.88(8)–91.56(8)
N(1)-C(2)	1.341(2)		

5.2 Preliminary X-ray crystal structure analysis of 5^{meso}

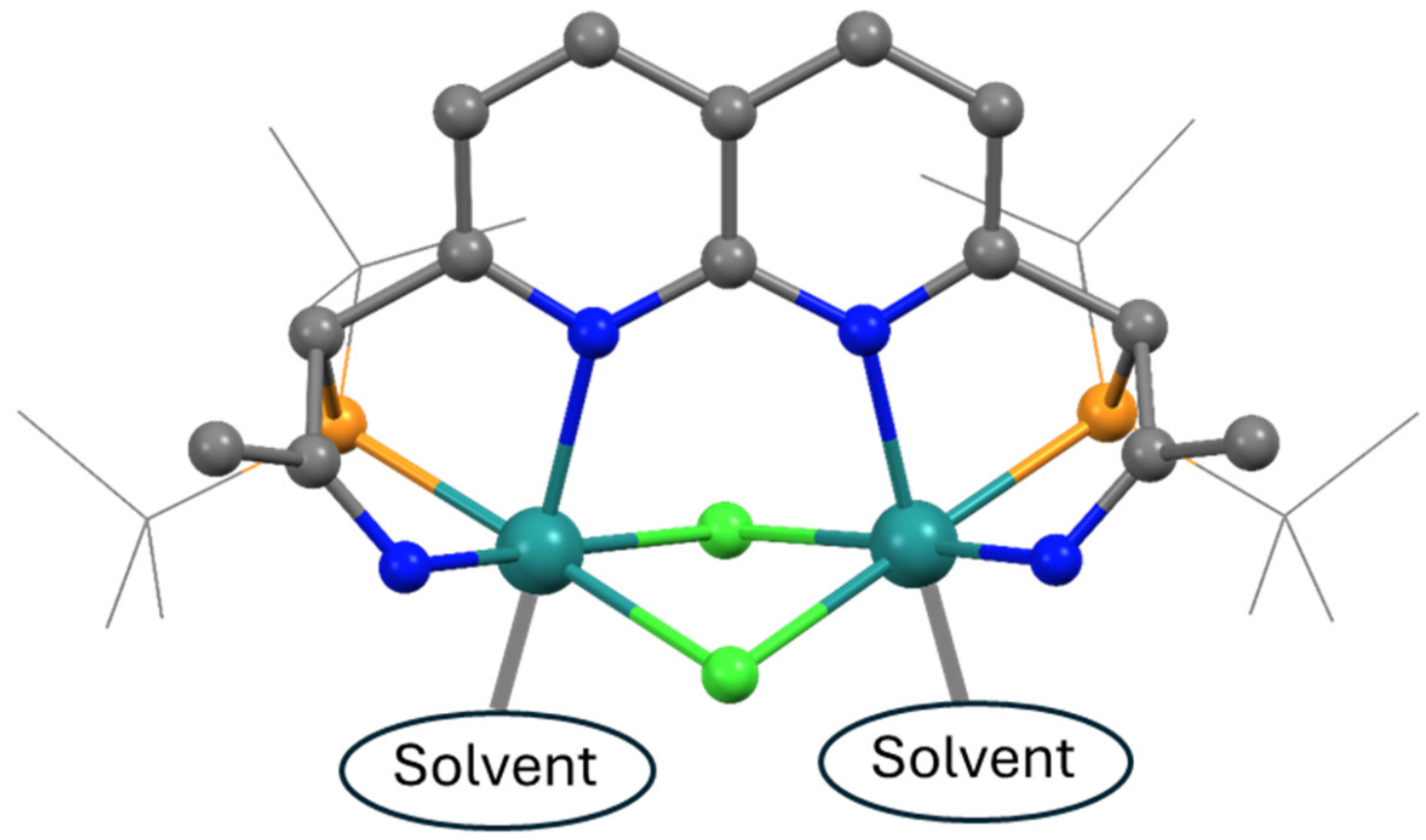

Figure S74. Molecular structure of complex $\mathbf{5^{meso}}$ derived from a preliminary poor-quality X-ray crystal structure. The solvent molecules coordinated to the Ru centers are heavily disordered hampering the determination of their exact nature. Hydrogen atoms, co-crystallized molecules of solvent, and PF_6 anions are omitted for clarity.

S5. References

(1) Kounalis, E.; Lutz, M.; Broere, D. L. J. Cooperative H2 Activation on Dicopper(I) Facilitated by Reversible Dearomatization of an "Expanded PNNP Pincer" Ligand. *Chem. – Eur. J.* **2019**, *25* (58), 13280–13284. https://doi.org/10.1002/chem.201903724.

(2) Kounalis, E.; Lutz, M.; Broere, D. L. J. Tuning the Bonding of a μ-Mesityl Ligand on Dicopper(I) through a Proton-Responsive Expanded PNNP Pincer Ligand. *Organometallics* **2020**, *39* (4), 585–592. https://doi.org/10.1021/acs.organomet.9b00829.

(3) Melnikov, S.; Chugh, V.; Hwang, D.; Baik, M.-H.; Broere, D. L. J. The Role of Quantum Tunneling in Metal–Ligand Proton Tautomerism. *J. Am. Chem. Soc.* **2025**, *147* (50), 46324–46335. https://doi.org/10.1021/jacs.5c15308.

(4) Parr, R. G.; Weitao, Y. *Density-Functional Theory of Atoms and Molecules*; Oxford University Press, 1994.

(5) Neese, F. The ORCA Program System. *WIREs Comput. Mol. Sci.* **2012**, *2* (1), 73–78. https://doi.org/10.1002/wcms.81.

(6) Neese, F. Software Update: The ORCA Program System, Version 4.0. *WIREs Comput. Mol. Sci.* **2018**, *8* (1), e1327. https://doi.org/10.1002/wcms.1327.

(7) Neese, F. Software Update: The ORCA Program System—Version 5.0. *WIREs Comput. Mol. Sci.* **2022**, *12* (5), e1606. https://doi.org/10.1002/wcms.1606.

(8) Neese, F. Software Update: The ORCA Program System—Version 6.0. *WIREs Comput. Mol. Sci.* **2025**, *15* (2), e70019. https://doi.org/10.1002/wcms.70019.

(9) Weigend, F.; Ahlrichs, R. Balanced Basis Sets of Split Valence, Triple Zeta Valence and Quadruple Zeta Valence Quality for H to Rn: Design and Assessment of Accuracy. *Phys. Chem. Chem. Phys.* **2005**, *7* (18), 3297–3305. https://doi.org/10.1039/B508541A.

(10) Andrae, D.; Häußermann, U.; Dolg, M.; Stoll, H.; Preuß, H. Energy-Adjustedab Initio Pseudopotentials for the Second and Third Row Transition Elements. *Theor. Chim. Acta* **1990**, *77* (2), 123–141. https://doi.org/ 10.1007/BF01114537.

(11) Neese, F. An Improvement of the Resolution of the Identity Approximation for the Formation of the Coulomb Matrix. *J. Comput. Chem.* **2003**, *24* (14), 1740–1747. https://doi.org/10.1002/jcc.10318.

(12) Helmich-Paris, B.; De Souza, B.; Neese, F.; Izsák, R. An Improved Chain of Spheres for Exchange Algorithm. *J. Chem. Phys.* **2021**, *155* (10), 104109. https://doi.org/10.1063/5.0058766.

(13) Weigend, F. Accurate Coulomb-Fitting Basis Sets for H to Rn. *Phys. Chem. Chem. Phys.* **2006**, *8* (9), 1057–1065. https://doi.org/10.1039/B515623H.

(14) Marenich, A. V.; Cramer, C. J.; Truhlar, D. G. Universal Solvation Model Based on Solute Electron Density and on a Continuum Model of the Solvent Defined by the Bulk Dielectric Constant and Atomic Surface Tensions. *J. Phys. Chem. B* **2009**, *113* (18), 6378–6396. https://doi.org/10.1021/jp810292n.

(15) Melnikov, S.; Verkooijen, M. W. P.; Verduin, J.; Martin Lutz; Broere, D. L. J. Unlocking Heterobimetallic Architectures in a Symmetric PNNP Ligand Environment. *ChemRxiv 2026* (0123). https://doi.org/10.26434/chemrxiv.10001527/v1.

(16) Ho, J.; Coote, M. L. A Universal Approach for Continuum Solvent pKa Calculations: Are We There Yet? *Theor. Chem. Acc.* **2010**, *125* (1), 3–21. https://doi.org/10.1007/s00214-009-0667-0.

(17) Kaljurand, I.; Kütt, A.; Sooväli, L.; Rodima, T.; Mäemets, V.; Leito, I.; Koppel, I. A. Extension of the Self-Consistent Spectrophotometric Basicity Scale in Acetonitrile to a Full Span of 28 p K_a Units: Unification of Different Basicity Scales. *J. Org. Chem.* **2005**, *70* (3), 1019–1028. https://doi.org/10.1021/jo048252w.

(18) Nerush, A.; Vogt, M.; Gellrich, U.; Leitus, G.; Ben-David, Y.; Milstein, D. Template Catalysis by Metal–Ligand Cooperation. C–C Bond Formation via Conjugate Addition of Non-Activated Nitriles under Mild, Base-Free Conditions Catalyzed by a Manganese Pincer Complex. *J. Am. Chem. Soc.* **2016**, *138* (22), 6985–6997. https://doi.org/10.1021/jacs.5b13208.

(19) Schreurs, A. M. M.; Xian, X.; Kroon-Batenburg, L. M. J. EVAL15: A Diffraction Data Integration Method Based on Ab Initio Predicted Profiles. *J. Appl. Crystallogr.* **2010**, *43* (1), 70–82. https://doi.org/10.1107/S0021889809043234.

(20) Herbst-Irmer, R.; Sheldrick, G. M. Refinement of Twinned Structures with SHELXL97. *Acta Crystallogr. B* **1998**, *54* (4), 443–449. https://doi.org/10.1107/S0108768197018454.

(21) Sevvana, M.; Ruf, M.; Usón, I.; Sheldrick, G. M.; Herbst-Irmer, R. Non-Merohedral Twinning: From Minerals to Proteins. *Acta Crystallogr. Sect. D Struct. Biol.* **2019**, *75* (12), 1040–1050. https://doi.org/10.1107/S2059798319010179.

(22) Sheldrick, G. M. SHELXT – Integrated Space-Group and Crystal-Structure Determination. *Acta Crystallogr. Sect. Found. Adv.* **2015**, *71* (1), 3–8. https://doi.org/10.1107/S2053273314026370.

(23) Sheldrick, G. M. Crystal Structure Refinement with *SHELXL*. *Acta Crystallogr. Sect. C Struct. Chem.* **2015**, *71* (1), 3–8. https://doi.org/10.1107/S2053229614024218.

(24) Spek, A. L. Structure Validation in Chemical Crystallography. *Acta Crystallogr. Sect. D Struct. Biol.* **2009**, *65* (2), 148–155.

(25) Krause, L.; Herbst-Irmer, R.; Sheldrick, G. M.; Stalke, D. Comparison of Silver and Molybdenum Microfocus X-Ray Sources for Single-Crystal Structure Determination. *J. Appl. Crystallogr.* **2015**, *48* (1), 3–10. https://doi.org/10.1107/S1600576714022985.
